# Synthesis and Complexation Behavior of Well-Defined Polyester-Based Polyelectrolytes with Varying Charge Densities and Hydrophobicities

**DOI:** 10.1021/acs.macromol.5c01795

**Published:** 2025-11-21

**Authors:** Julian Engelhardt, Louis C.P.M. de Smet, Evelien Maaskant, Jasper van der Gucht

**Affiliations:** 1 Laboratory of Organic Chemistry, 4508 Wageningen University, Stippeneng 4, Wageningen 6708 WE, The Netherlands; 2 Wageningen Food and Biobased Research, Bornse Weilanden 9, Wageningen 6708 WG, The Netherlands; 3 Laboratory of Physical Chemistry and Soft Matter, 4508 Wageningen University, Stippeneng 4, Wageningen 6708 WE, The Netherlands

## Abstract

In this work, we describe the synthesis of polyester-based polyelectrolytes with varying charge densities and examine the complexation behavior of the corresponding polyelectrolyte complexes (PECs). The polyelectrolytes were prepared via ring-opening polymerization (ROP) of α-bromo-ε-caprolactone and subsequent postmodification with functionalized thiols, yielding oppositely charged polyelectrolytes with different charge densities ranging from 59 to 100%. From this, nine combinations of complexes were produced, and their complexation behavior and critical salt concentration (CSC) were investigated. All polyelectrolyte mixtures show a strong tendency to undergo solid–liquid phase separation even at salt concentrations close to the CSC. Liquid–liquid complex coacervation is only observed, close to the CSC, for the complex containing polyelectrolytes with the highest charge densities. A nonmonotonic dependence of the CSC on the charge density is observed, where the combination of the polyelectrolytes with the lowest charge densities and highest hydrophobic contents yields the highest CSC. Furthermore, complexes containing polyelectrolytes with very different charge densities tend to have a lower CSC. These findings imply that interactions other than electrostatics play a role in complexation. We interpret our results using a mean-field theory for polyelectrolyte complexation, accounting for electrostatic and nonelectrostatic interactions. Our findings pave the way for developing novel polyester-based materials with controllable material properties.

## Graphical abstract

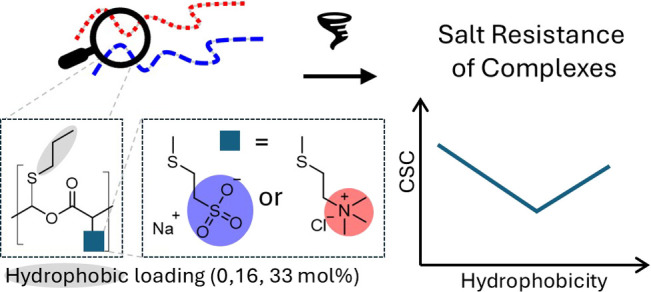

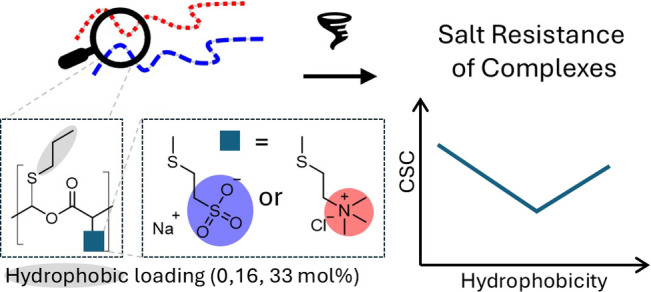

## 1 Introduction

Plastics are widely used because of their performance, low weight and cost efficiency. The durable character of plastics, however, creates diverse pollution hazards. To mitigate this, several collection, reuse, and recycling strategies have been set in place, yet a significant amount of plastic is leaking into the environment and eventually accumulating in the oceans.^1–4^ By designing plastic materials containing an on-demand trigger, which allows the material to degrade under specific environmental conditions, such as in seawater, plastic accumulation could be reduced.^5^

An interesting class of materials that could fulfill this requirement are so-called polyelectrolyte complexes (PECs) made from oppositely charged polyelectrolytes. When aqueous solutions of oppositely charged polyelectrolytes are combined, PECs are formed depending on the salt concentration.^6,7^ At sufficiently low salt concentrations, a solid–liquid (S-L) phase separation can be observed and the solid PEC can be recovered from the solution and processed via centrifugation,^8,9^ extrusion^10–13^ or hot-pressing.^14,15^ The resulting PECs have been used in, among others, membrane technology and cartilage mimics applications.^8,16–19^ At higher salt concentrations, the salt ions screen the ionic cross-links, decreasing their strength resulting in liquid–liquid (L-L) phase separation, also called complex coacervation. These complex coacervates are well-hydrated, loosely associated PECs that can find application in the field of drug delivery and underwater adhesives.^20^ When the so-called critical salt concentration (CSC), i.e. the concentration at which phase separation no longer occurs,^21^ is reached, the ionic cross-links between the oppositely charged polyelectrolytes are broken and the complex dissociates. Hence, ionic strength plays a crucial role in tuning the properties of the PECs by modulating the ionic cross-links. While the rather brittle mechanical properties of dried PECs can be partly overcome by the addition of salt, most PECs are primarily used in hydrated environments.^19^ Other factors that can influence the complexation and also the final properties of the PEC are the charge density^22–25^ of the polyelectrolytes and their hydrophobicity.^26–29^

Currently, a wide range of synthetic polyelectrolyte complexes has been reported, mostly based on polymers with an all-carbon backbone.^19^ In contrast to a C–C backbone, a polyester backbone offers benefits, as the ester bond can (bio)­degrade in natural environments via hydrolysis or enzymatic degradation pathways.^5,30^ This prompted us to design, prepare, and characterize polyester-based saloplastics obtained via melt polycondensation.^15^ However, these first-generation polyester saloplastics suffered from impediments such as a high dispersity (*Đ*) and low molecular weight (*M*_w_) of the polyelectrolyte(s), discoloration, significant moisture sensitivity, and little control on charge density.

To overcome these limitations, we here report on a new synthetic approach to obtain well-defined polyester-based polyelectrolytes. This approach is based on the synthesis of functionalized polyesters via ring-opening polymerization (ROP) of α-bromo-ε-caprolactone (αBrCL), allowing for precise control over *M*_w_ and lower *Đ*. The charge density of the polyelectrolytes was controlled via postmodification of the polymer backbone with functionalized thiols for both the polyanion and polycation and hydrophobic functionalities (Scheme 2). Furthermore, we show the effect of charge density on the complexation behavior, the resistance against salt, and the composition of the individual polyelectrolytes in the complex, aided by mean-field modeling.

## 2 Experimental Section

### 2.1 Chemicals and Materials

Cyclohexanone (≥99.0%), sodium 2-mercaptoethanesulfonate (≥95%), and ethyl acetate (≥99%) were obtained from TCI Europe. Bromine (≥99.6%) and potassium trifluoroacetate (≥98%) were purchased from Fisher Scientific. Dichloromethane (≥99.5%), meta-chloroperbenzoic acid (*m*-CPBA) (≥77%), anhydrous toluene (≥99.8%), tin­(II) 2-ethyl hexanoate (Sn­(Oct)_2_) (92.5–100%), anhydrous benzyl alcohol (≥99.8%), triethylamine (≥99%), anhydrous DMF (≥99.8%), 2-(dimethylamino) ethanethiol hydrochloride (MEDA) (95%), potassium bromide (≥99%), and iodomethane (99%, stabilized) were purchased from Sigma-Aldrich. *n*-Hexane (≥97.0%) was purchased from Honeywell. Anhydrous MeOH (≥99.8%) was obtained from Macron Fine Chemicals. 1,1,1,3,3,3-Hexafluoro-2-propanol (HFIP, 99%) was obtained from Fluorochem. Sodium chloride (NaCl > 99.5%) was obtained from J.T. Baker. All experiments were performed with Milli-Q water (18 mΩ) unless stated differently. All chemicals were used as received.

### 2.2 Synthesis of Poly­(α-bromo-ε-caprolactone)

#### 2.2.1 Synthesis of α-Bromo-ε-caprolactone

The synthesis of α-bromo-ε-caprolactone was adapted from literature (see Scheme 1).^31^ First, α-bromocyclohexanone was prepared by mixing cyclohexanone (78 g, 0.79 mol) and distilled water (500 mL) in a 2 L round-bottom flask. Bromine (41.1 mL, 0.79 mol) was added dropwise over a period of 2 h during which the temperature was maintained between 5 and 10 °C by external cooling in an ice bath. The stirring was continued until the reaction mixture was colorless (about 4 h). The now heavy organic layer was separated from the aqueous layer, dried over anhydrous MgSO_4_, filtered, and then the filter cake was washed with DCM. The organic solvent was then removed in vacuo and the crude product was distilled to obtain α-bromocyclohexanone as a pale-yellow oil (94 g, 67% yield), with spectral data in line with those reported in literature.^31^

**Scheme 1:**
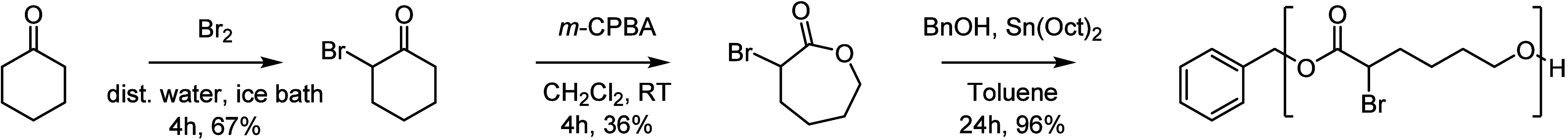
Synthetic Route for the Preparation of P­(αBrCL) Using Cyclohexanone as the Starting Material

^1^H NMR (400 MHz, CDCl_3_): δ 1.64–2.03 (m, 4H, – C*H*_2_C*H*_2_−), 2.13–2.23 (m, 1H, COC*H*_2_), 2.30 (m, 2H, – C*H*_2_−), 2.89–2.93 (m, 1H, COC*H*_2_), 4.40–4.42 (t, 1H, C*H*–Br).

Next, *m*-CPBA (146 g, 0.85 mol) was added to a solution of α-bromocyclohexanone (94 g, 0.53 mol) in 500 mL of DCM. After stirring in an ice bath for 1 h, the reaction was then stirred at room temperature overnight. The suspension was transferred to a freezer for 3 h to precipitate the side product 3-chlorobenzoic acid. The solution was then filtered and washed with sat. Na_2_S_2_O_3_ solution (4 × 100 mL), sat. NaHCO_3_ solution (4 × 100 mL), and finally with distilled water until the solution had a neutral pH. The organic phase was dried with anhydrous MgSO_4_ overnight in the fridge. The MgSO_4_ was removed by filtration, and the solvent was removed in vacuo. The crude product was purified using a silica plug (height 8 cm, ⌀ 10 cm) using *n*-hexane:EtOAc (9:1) as eluent to remove residual α-bromocyclohexanone, after which an increasing eluent polarity (7:3 *n*-hexane:EtOAc) was used for the collection of the second fraction. The solvent was removed under vacuo, and the transparent oil was transferred into the freezer at −20 °C, inducing crystallization. The resulting white crystals of α-bromo-ε-caprolactone were washed with hexane and dried in the vacuum oven at 50 °C overnight (37 g, 36% yield). Spectral data is in line with literature.^31^

^1^H NMR (400 MHz, CDCl_3_): δ 2.11 (m, 6H, – C*H*_2_C*H*_2_C*H*_2_−), 4.28–4.71 (m, 2H, – COOC*H*_2_−), 4.85 (t, 1H, – C*H*(Br)−).

#### 2.2.2 Ring-Opening Polymerization

The ring-opening polymerization of α-bromo-ε-caprolactone was adapted from literature.^32,33^ ROP was performed in a predried 250 mL Schlenk flask in an N_2_ MBRAUN MB10 compact glovebox equipped with a gas purification system providing a dry and inert nitrogen atmosphere (H_2_O and O_2_ level <0.5 ppm). α-Bromo-ε-caprolactone (36.1 g, 0.19 mol) was dissolved in 100 mL anhydrous toluene. A stock solution of Sn­(Oct)_2_ (38.0 mM) and benzyl alcohol (190.0 mM) was prepared in 10 mL of anhydrous toluene, and both solutions were purged by three cycles of freeze–pump–thaws. The reaction vessel and stock solution were transferred into the glovebox. Subsequently, 2 mL of the stock solution was added via a Hamilton syringe to obtain a composition of monomer, initiator, and catalyst (500/1/0.2) targeting a DP of 500.

The reaction was carried out at 130 °C under continuous stirring. The conversion of the reaction was monitored via ^1^H NMR with the vanishing of the C*H*—Br (δ = 4.84 ppm) and —COOC*H*_2_— (δ = 4.28 and 4.71 ppm) signals, and the appearance of a new peak belonging to the overlapping signals of C*H*–Br and —COC*H*_2_— at δ = 4.19 ppm. The heating was stopped at almost full conversion (>95%, 24 h, ^1^H NMR overlay in Figure S4), and the reaction was quenched by exposure to air. The highly viscous solution was diluted with 100 mL DCM and precipitated twice in cold MeOH. The polymer was dried under reduced pressure at 50 °C overnight (34.0 g, 96% yield). The product, poly­(α-bromo-ε-caprolactone) , hereafter, P­(αBrCL), was characterized by ^1^H NMR, ^13^C NMR, DOSY NMR, size exclusion chromatography (SEC), thermogravimetric analysis (TGA) and differential scanning calorimetry (DSC) (SI).

^1^H NMR (400 MHz, CDCl_3_): δ 1.85–1.39 (m, 4H,–C*H*_2_C*H*_2_–_backbone_), 2.22–1.96 (m, 2H, BrC*H*_2_), 3.63–3.69 (m, 2H, – C*H*_2_O_chain end_), 4.30–4.10 (m, 3H, C*H*–Br and COC*H*_2_), 5.20 (s, 2H, C*H*_2_O_benzyl protons_), 7.36–7.39 (m, 5H, Ar–H). ^13^C NMR (101 MHz, CDCl_3_) δ 23.15 (*C*H_2_), 27.13 (*C*H_2_), 33.69 (*C*H_2_), 45.04 (*C*H_2_), 45.07 (*C*H_2_), 64.85 (*C*H–Br), 169.04 (*C*O). SEC (HFIP + 0.02 M potassium trifluoroacetate): *M*_n_ = 93 kDa *M*_w_ = 112 kDa, (*Đ*) = 1.20. TGA: 5% weight loss at 323 °C and DSC: *T*_g_ −25 °C.

### 2.3 Postmodification of P­(αBrCL)

The purified polymer was divided into multiple fractions for further functionalization to yield polyanions and polycations having different charge densities, as described in Scheme 2. The general functionalization reaction was adapted from Dai et al.^32^

**Scheme 2:**
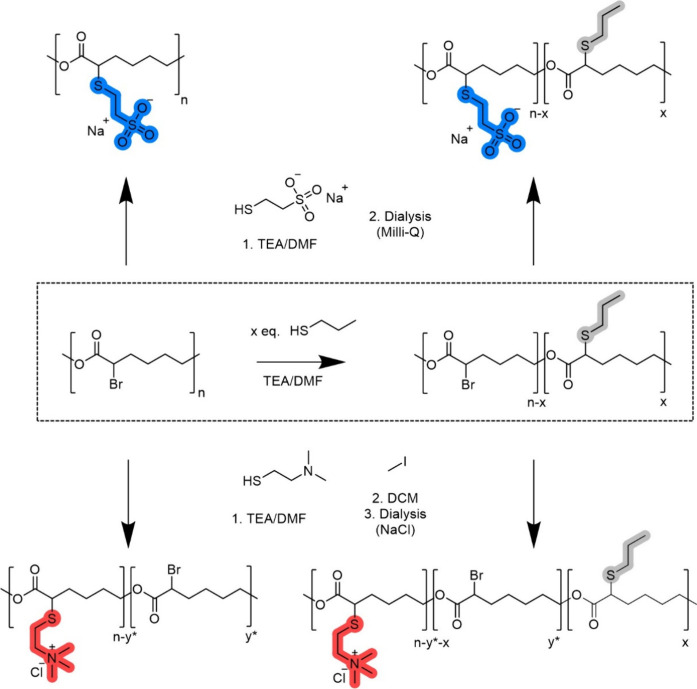
Functionalization Strategy of P­(αBrCL) toward Anionic (Blue, Top) and Cationic (Red, Bottom) Polyelectrolytes with Varying Charge Density^a^

a Polyelectrolytes with lower charge density were obtained by first partly functionalizing the polymer with propane thiol (dashed box, grey), followed by conversion to the corresponding anionic (blue) and cationic (red) polyelectrolytes. The incomplete substitution of the bromine in the polycations is indicated by y*, of which the value differs for each polycation.

#### 2.3.1 Partial Functionalization with Propanethiol

P­(αBrCL) was partly functionalized with propanethiol to reduce the number of available bromine groups in subsequent functionalization reactions. The conversion was calculated via the ratio between the α-proton of the thiol and the polymer backbone (^1^H NMR overlay, Figure S7).

Example procedure for partial substituting (16%) of the bromine: P­(αBrCL) (10.17 g, 52.7 mmol bromine group) was dissolved in 120 mL DMF by stirring with a magnetic stirring bar. Subsequently, propanethiol (711 mg, 9.3 mmol) and triethylamine (944 mg, 9.3 mmol) were added slowly into the mixture. The colorless solution was stirred for 30 min and the polymer solution was precipitated in cold MeOH. The precipitated polymer P­(αBrCL)-(84%) was washed with MeOH and dried in the vacuum oven at 50 °C overnight (9.66 g, 95%). The same conditions were used to prepare P­(αBrCL)-(67%) (8.56 g, 85%).

P­(αBrCL)-(84%): ^1^H NMR (400 MHz, CDCl_3_): δ 4.25–4.00 (m, 18H), 3.14 (t, *J* = 7.5 Hz, 1H), 2.51 (m, *J* = 12.5, 7.2 Hz, 2H), 2.13–1.90 (m, 11H), 1.90–1.77 (m, 1H), 1.75–1.29 (m, 30H), 0.91 (t, *J* = 7.3 Hz, 3H). SEC (HFIP + 0.02 M potassium trifluoroacetate): *M*_n_ = 88 kDa, *M*_w_ = 117 kDa, *Đ* = 1.33.

P­(αBrCL)-(67%): ^1^H NMR (400 MHz, CDCl_3_): δ 4.20–4.02 (m, 9H), 3.14 (t, *J* = 8.8, 7.2, 1.7 Hz, 1H), 2.51 (m, *J* = 12.5, 9.5, 6.7 Hz, 2H), 2.13–1.76 (m, 6H), 1.39 (s, 17H), 0.91 (t, *J* = 7.3 Hz, 3H). SEC (HFIP + 0.02 M potassium trifluoroacetate): *M*_n_ = 88 kDa, *M*_w_ = 124 kDa, *Đ* = 1.41.

#### 2.3.2 Subsequent Functionalization toward Polyelectrolytes

The partly functionalized polymers P­(αBrCL)-(67%) and (84%) were divided into two batches of equal size, and the corresponding polyanions and polycations were obtained according to the described procedures. An overview of their ^1^H NMR and the nonfunctionalized P­(αBrCL) can be found in the SI (Figures S5–S13). The amounts and yields are given in Table S1. DOSY NMR measurements were performed on all purified polyelectrolytes to confirm the successful functionalization of the polymer and to exclude degradation of the polymer backbone during functionalization (SI, Figures S31–S38).

##### 2.3.2.1 Synthesis of Polyanions

An example procedure for P100^⊖^ is given. P­(αBrCL) (5.07g, 25.9 mmol bromine group) was dissolved in 120 mL DMF by stirring with a magnetic stirring bar. After complete dissolution, sodium 2-mercaptoethanesulfonate was added in small portions, turning the colorless solution into a cloudy suspension. Triethylamine was added slowly into the mixture, turning the mixture into a transparent solution again. After 30 min, a highly viscous precipitate formed. The solid precipitate was recovered from the solution, dissolved in ∼ 30 mL of water, and transferred into a dialysis tube (spectra/por6, MWCO 1 kDa), where it was dialyzed against 0.5 M NaCl and further against pure water for 3 days each. The lysate was exchanged twice a day. In addition, the transparent top layer of the functionalization reaction was decanted and precipitated in cold acetone. The obtained precipitate was purified similarly via dialysis. Both dialysis fractions were combined, and the resulting polymer solution was concentrated on the rotary evaporator and further dried in the vacuum oven at 50 °C overnight (6.25g, 22.6 mmol, 87%). An overview of the ^1^H NMR spectra of all polyanions is given in Figure S8–S10.

P100^⊖^: ^1^H NMR (400 MHz, D_2_O): δ 1.39–1.65 (m, 2H), 1.69–1.85 (m, 3H), 1.86–2.01 (m, 1H), 2.91–3.08 (m, 2H), 3.09–3.26 (m, 2H), 3.50–3.64 (m, 1H), 4.17–4.35 (m, 2H).

P84^⊖^: ^1^H NMR (400 MHz, D_2_O): δ 1.02–1.10 (m, 3H), 1.59 (m, 2H), 1.75–1.92 (m, 3H), 1.99 (s, 1H), 2.65–2.75 (m, 2H) 3.06 (m, 2H), 3.21 (m, 2H), 3.42–3.53 and 3.54–3.69 (m, 1H), 4.19–4.40 (m, 2H).

P67^⊖^: ^1^H NMR (400 MHz, D_2_O): δ 0.95–1.10 (m, 3H), 1.43–1.69 (m, 4H), 1.70–1.88 (m, 3H), 1.88–2.03 (m, 1H), 2.56–2.76 (m, 2H), 2.93–3.07 (m, 2H), 3.08–3.28 (m, 2H), 3.29–3.41 and 3.47–3.59 (m, 1H), 4.12–4.37 (m, 2H).

##### 2.3.3.3 Synthesis of Polycations

An example procedure for P87^⊕^ is given, for which a charge density of 100% was targeted. In detail: P­(αBrCL) 4.80 g (24.9 mmol bromine group) was dissolved in 200 mL DMF by stirring with a magnetic stirring bar. Subsequently, MEDA (6.76 g, 49.7 mmol) and triethylamine (6.65 g, 49.7 mmol) were added slowly into the mixture. The colorless solution was stirred for 30 min and the polymer solution was diluted with 100 mL DCM. The polymer solution was washed with brine (5 × 300 mL) and dried over Na_2_SO_4_. DCM was removed under reduced pressure and the polymer was further dried in the vacuum oven at 50 °C overnight (4.25 g, 18.8 mmol, 75%).

The functionalized polymer was dissolved in 120 mL DMF and 10 mL of a 25% (v/v) solution of MeI in DMF was slowly dropped into the solution. Subsequently, the colorless solution turned bright yellow. After 2 h the mixture was precipitated in cold ether. The polymer was washed three times with 200 mL of ether and dried under vacuum. The polymer was dissolved in ∼ 50 mL H_2_O, and the mixture was transferred to a dialysis tube (spectra/por6, MWCO 1 kDa). First, it was dialyzed against 0.5 M NaCl and further against pure water for 3 days each. The lysate was exchanged twice a day. The polymer solution was concentrated on the rotary evaporator and further dried in the vacuum oven at 50 °C overnight. (3.66 g, 16.2 mmol, 86%). An overview of the ^1^H NMR spectra of all polycations is given in Figure S11–S13. Based on the ^1^H NMR spectrum it can be concluded that the substitution of the bromine was incomplete. Thus, the polyelectrolytes were labeled according to their actual charge densities: P87^⊕^, P77^⊕^, and P59^⊕^. For a more elaborate discussion on this, we refer to the Results and Discussion section.

P87^⊕^: ^1^H NMR (400 MHz, D_2_O): δ 1.39–1.67 (m, 2H), 1.70–1.88 (m, 3H), 1.88–2.02 (m, 1H), 3.07–3.15 (m, 2H), 3.16–3.24 (m, 9H), 3.54–3.69 (m, 3H), 4.20–4.34 (m, 2H). Remaining bromine: 13%.

P77^⊕^: ^1^H NMR (400 MHz, D_2_O): δ 0.97–1.06 (m, 3H), 1.41–1.70 (m, 4H), 1.69–1.89 (m, 3H), 1.88–2.06 (m, 1H), 2.61–2.69 (m, 2H), 3.08–3.17 (m, 2H), 3.17–3.30 (m, 9H), 3.35–3.47 and 3.57–3.70 (m, 1H), 4.15–4.39 (m, 2H). Remaining bromine: 7%.

P59^⊕^: ^1^H NMR (400 MHz, D_2_O): δ 0.85–1.00 (m, 3H), 1.29–1.98 (m, 7H), 2.45–2.66 (m, 1H), 2.95–3.10 (m, 1H), 3.08–3.21 (m, 5H), 3.45–3.67 (m, 2H), 4.00–4.29 (m, 2H). Remaining bromine: 8%.

### 2.4 Complexation

The complexation experiments were performed following a previous study.^34^ Stock solutions of each polyelectrolyte (50 mM) according to their average repeating unit molecular weight (displayed in Scheme S1) were obtained by dissolving the oven-dried polyelectrolyte in Milli-Q water (∼13 g/L). By combining all variations of oppositely charged polyelectrolytes, nine different complex combinations have been obtained. The complexation behavior of these nine complexes was investigated at different salt concentrations ranging from no added salt up to 2.0 M KBr. (Precise amounts are given in Table S2).

In detail, 400 μL of polycation solution was pipetted using positive displacement pipettes (MICROMAN E M1000E, 1000 μL, Gilson) into a glass vial (⌀ 1 cm) together with a certain amount of 4 M KBr stock solution and Milli-Q water so that the total volume was 1.6 mL. The mixture was homogenized via vortexing for 5 s. Finally, 400 μL of polyanion stock solution was added to yield a mixture (total volume 2.0 mL) with a polyelectrolyte concentration of 20 mM (according to the average repeating unit molecular weight) and the previously calculated salt concentration. Complexation occurred instantly upon the addition of the polyanion in cases where the salt concentration was low enough. The complex was obtained as a pale white, gel-like substance. The mixture was vortexed for 30 s vigorously and left to equilibrate for 5 days in total at room temperature on a spinning wheel (40 rpm). The supernatant phase was investigated by absorbance measurements at a wavelength of 500 nm, the obtained values are given in Table S3.^23^ After equilibration, the complex mixture was centrifuged, and the supernatant was decanted from the solid PEC. The PEC was dried under vacuum at 50 °C overnight.

### 2.5 Characterization

NMR measurements were conducted on a 400 MHz Bruker Advance III at 298 K, and the resulting data were analyzed using MestReNova software, version 15.0.0–34764. The ^13^C NMR spectra (101 MHz) were measured with 2048 scans, and 1 s relaxation delay. The DOSY NMR spectra were measured with 32 scans, and 3 s relaxation delay. Measurements in CDCl_3_ were calibrated at the CHCl_3_ peak at 7.26 ppm for ^1^H NMR spectra, and 77.16 ppm for ^13^C spectra. Spectra measured in D_2_O were calibrated at the D_2_O peak at 4.79 ppm (^1^H NMR spectra). Abbreviations used in the description of NMR data are as follows: chemical shift (δ in ppm), multiplicity (s = singlet, d = doublet, t = triplet, q = quartet, p= pentet, sext = sextet, h = heptet, dt = doublet of triplets, m = multiplet, br = broadened), coupling constant (*J*, Hz). The composition of the PECs was determined according to the literature.^10^

The supernatant and PEC were analyzed separately. Turbidity measurements were performed on the supernatant phase with a UV–vis spectrophotometer (Shimadzu UV-2600) by measuring the absorbance at 500 nm at 25 °C in 10 × 10 mm^2^ plastic cuvettes.^23^ The PECs were dissolved in 2.5 M KBr D_2_O solution and analyzed with ^1^H NMR and DOSY NMR.

The molecular weights of the noncharged polyesters were determined using an OmniSEC HP-SEC system (model CHR6000 sn. MAL 1202615) equipped with a triple detector array (right-angle light scattering (RALS), low-angle light scattering (LALS), and refractive index (RI) detector and viscometer) on HFIP equipped with two × SEC columns (PSS, PFG, analytical, linear M), and a guard column, molecular range of 250–2.5 × 10^6^ D (PMMA in HFIP). Data were calculated with OmniSEC software (version 11.32) assuming a 100% mass recovery with the d*n*/d*c* values obtained from the individual sample concentrations. Hexafluoroisopropanol (HFIP) containing 0.02 M of potassium trifluoroacetate was used as the eluent with 100 μL injection volume and a flow rate of 0.7 mL/min. Samples of a concentration 2–3 mg/mL were dissolved overnight and filtered over a 0.45 μm PTFE filter before injection. Narrow standard PolyCal PMMA 50 kDa (from Viscotek) was used for absolute calibration of the system (*M*_w_ = 51.550 g/mol, *Đ* = 1.024).

The thermal stability of the polyesters was determined by using a PerkinElmer STA6000 by heating the samples (∼5 mg) from room temperature to 400/700 °C at 10 °C/min under a continuous flow of nitrogen gas or air (20 mL/min).

Differential scanning calorimetry (DSC) measurements were performed using a PerkinElmer DSC 8000 provided with liquid nitrogen cooling and an autosampler. Stainless steel DSC cups with rubber O-rings were used. The following protocol was used: two scans from – 70 to 140 °C with a heating rate of 10 °C/min with 10 min isothermal steps at the limits and cooling rate of 10 °C/min.

Images of the complexation experiments of the PECs were acquired using a ZEISS microscope (Axio Observer 7) equipped with Light Source Colibri 5 (Type RGB-UV), a ZEISS Plan-NEOFLUAR 20 × /0.5 objective, and a 90 HE LED filter set. For bright-field visualization, images were acquired at an exposure of 10 ms with 1.5 V light intensity, using a Prime BSI Express CMOS camera.

## 3 Results and Discussion

### Polymer Synthesis

The procedure of the ring-opening polymerization of αBrCL (Scheme 1) was adapted from literature,^31–33^ and found to be successful at a scale of 35 g with a conversion and yield over 95%. Triple-detection SEC of the purified polymer showed that a high molecular weight polymer was obtained (*DP*∼480, *M*_n_ = 93 kDa, *Đ* = 1.2).

Subsequently, the polymer was functionalized to yield anionic and cationic polyelectrolytes with varying charge densities, as shown in Scheme 2. To avoid the formation of block-like polymer domains in polyelectrolytes with less than 100% charge density, the functionalization was performed as a two-step reaction. First, the polymer was partially functionalized with propanethiol as adapted from literature procedures to obtain a random copolymer.^32^ The degree of the functionalization was calculated via ^1^H NMR by the ratio of the signal of the bridgehead proton of the functionalized polymer at δ = 3.20 ppm and the combined signal of the nonsubstituted bridgehead proton signal (C*H*—Br) and signals of (—COOC*H*_2_—) at δ = 4.20 ppm in Figure S5 and S6. A partial substitution of the bromine atoms by propanethiol was achieved to a degree of 16% and 33%, respectively, as shown by ^1^H NMR in Figure S7. SEC measurements on both partly functionalized polymers showed no significant changes in the molecular weight (*M*_n_ = 88 kDa, *Đ* = 1.33–1.41), indicating that the ester backbone was stable during functionalization in Figure S40.

The synthesis of the polyanions from the nonfunctionalized and the two partly functionalized polymers was adapted from literature.^32^ The obtained polyanions are labeled with their charge density and type of charge, i.e. P100^⊖^, P84^⊖^, and P67^⊖^. All polymers were well-soluble in water at room temperature. ^1^H NMR analysis proved the successful substitution of the bromine by sodium 2-mercaptoethanesulfonate, as illustrated in Figure S8–S10. The ^1^H NMR signal of the substituted bridgehead proton (C*H*—CH_2_CH_2_SO_3_) shifted upfield to δ = 3.57 ppm and separated from the signal (—COOC*H*_2_ —) at δ = 4.25 ppm. For the partly functionalized polyanions P84^⊖^ and P67^⊖^, the bridgehead hydrogen showed two distinct peaks at δ = 3.60 ppm (C*H*—CH_2_CH_2_SO_3_) and δ = 3.49 ppm. (—COOC*H*_2_ —), which reflects the ratio of their functionalization. The modification into the polyanion was further confirmed with ^13^C NMR in Figure S24–S26 and DOSY NMR measurements in Figure S31–S33 and S37. All polyanions were obtained as slightly hygroscopic and slightly opaque colorless materials.

The synthesis of the polycations was adapted from a literature procedure from Dai et al. with the addition of a methylation step yielding a quaternary ammonium salt.^32^ The obtained polymers are labeled analogous to the polyanions. Similar to the polyanion synthesis, the bromine with MEDA, i.e. the amine-functionalized thiol, (C*H*—CH_2_CH_2_NMe_2_) the bridgehead proton and —COOC*H*_2_ — signals shifted upfield to δ = 3.25 ppm and δ = 4.12 ppm, respectively, upon substitution. However, the integration values of the three protons (—SC*H*— and —SC*H*_*2*_—) at δ = 4.25 ppm indicate incomplete substitution, namely ca. 87%, while targeting full substitution of the bromine. Attempts to drive the conversion further resulted in an insoluble, dark brown, rubbery material, and they were therefore not pursued further. We hypothesize that a nucleophilic attack from the amine could result in cross-linking of the polymer. Similar results have been obtained for the partially functionalized polycations of P­(αBrCL)-(67%) and (84%), resulting in final substitution ratios of the bromine atoms of 93% and 92%, respectively, as indicated by ^1^H NMR in Figures S11-13 **.**

Next, the purified polymers functionalized with MEDA were reacted with iodomethane. After precipitation in ice-cold ether, a white rubbery solid was obtained. The singlet corresponding to the protons of the three methyl groups (—NMe_3_) of the quaternary ammonium salt, shifted from δ = 2.23 ppm to δ = 3.19 ppm. The methylene signal (—C*H*_2_NMe_3_) shifted after alkylation downfield and overlaps with the signal of the bridgehead proton (C*H*—CH_2_CH_2_NMe_3_) at δ = 3.60 ppm. The obtained polymers were dialyzed to exchange the iodine against chloride to yield polyelectrolytes, which were labeled according to their actual charge density: P87^⊕^, P77^⊕^_,_ and P57^⊕^. The successful modification into the polycation was further confirmed with ^13^C NMR in Figures S27–S29 and DOSY NMR measurements in Figures S34–S36 and S38. The polycations were obtained as hygroscopic transparent materials.

This synthesis strategy, which includes a postmodification step, enables the gram-scale synthesis of six polyester-based polyelectrolytes with varying charge densities. With a decrease in charge density and an increase in hydrophobicity, the hygroscopic properties of both the polyanion and polycation decreased. Overall, compared to the polycations, the polyanions dissolved faster in water, which could relate to the higher charge density of the polyanions compared to the polycationic counterpart. Next, we investigated the complexation behavior of the different combinations of oppositely charged polyelectrolytes.

### Complexation

The effects of molecular weight and dispersity on the complexation behavior of polyelectrolytes^9,23,26,29,35–39^ can be minimized by synthesizing a homologous polymer series with narrow dispersity. Similar approaches have been reported by the groups of Laaser,^23,29^ Tirrell,^22^ and Perry^40^ for acrylate-based, ether-based, and methacrylate-based polyelectrolytes, respectively. By dividing the polymer batch into six parts and subsequently substituting the bromine with the appropriate thiol, the DP is identical for all polyester-based polyelectrolytes. This approach enables us to study exclusively the effect of charge density and hydrophobicity on the complexation behavior.

The phase behavior of the synthesized polyelectrolytes was studied using equal molar concentrations of their repeating units, as shown in Scheme S1 and eq S1. Note that all complexes are labeled with polycation-polyanion, e.g. by combining P87^⊕^ with P84^⊖^, the complex 87^⊕^-84^⊖^ is obtained. The effect of (im)­balances of hydrophobicity and charge density was investigated as a function of increasing salt concentration for all nine combinations of polyelectrolytes.

Without the addition of salt during the complexation, a cloudy solution was obtained for all PEC combinations. This suggests imperfect pairing of the polyelectrolytes, yielding small aggregates that may be stabilized by an excess of charge. At low salt concentrations, solid–liquid (S-L) phase separation was observed for all combinations of polyelectrolytes, as shown in Figure 1 and Figure 2. The as-obtained solid complexes were found to be white, rubbery, and elastic materials. As the salt concentration increases, a two-phase system was obtained consisting of a solid complex and a transparent supernatant phase, probably because the additional ions screen the ionic interactions between the previously observed colloidal aggregates and allow proper rearrangement of the polyelectrolyte chains.^8,35^ Increasing the salt concentration further screens the electrostatic attraction between the opposite charges^21^ and thereby decreases the formation of solid complexes until finally a homogeneous one-phase system is obtained at the so-called critical salt concentration (CSC).^35^ Turbidity measurements of the mixtures were performed with UV–vis spectroscopy close to the CSC, and the absorption values are given in Table S3.^23^ Mixtures with enhanced turbidity were further analyzed with brightfield microscopy to check for liquid–liquid (L-L) phase separation, also called coacervation. Coacervate droplets were only observed for the 87^⊕^-100^⊖^ mixture at 1.50 M KBr under the optical microscope, as visualized in Figure 1 and Figure S59. For the combinations 77^⊕^-84^⊖^, 59^⊕^-67^⊖^, 87^⊕^-84^⊖^, and 87^⊕^-67^⊖^ at various salt concentrations close to the CSC, no coacervate droplets were detected, and instead, small amounts of solid complex were observed (Figure S60b and c). Potentially, these and other complexes may form coacervates in a small regime of salt concentrations that was not investigated.

**Figure 1:**
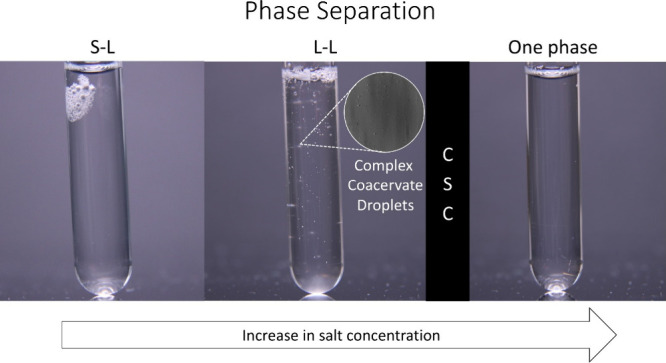
Images of complex (87^⊕^-100^⊖^) at different KBr concentrations showing different types of phase separation. S-L phase separation is obtained from 0 M-1.25 M, L-L (microscopic image of coacervate droplets, Figure S59b) around 1.50 M, and a one-phase system is obtained above 1.75 M.

**Figure 2:**
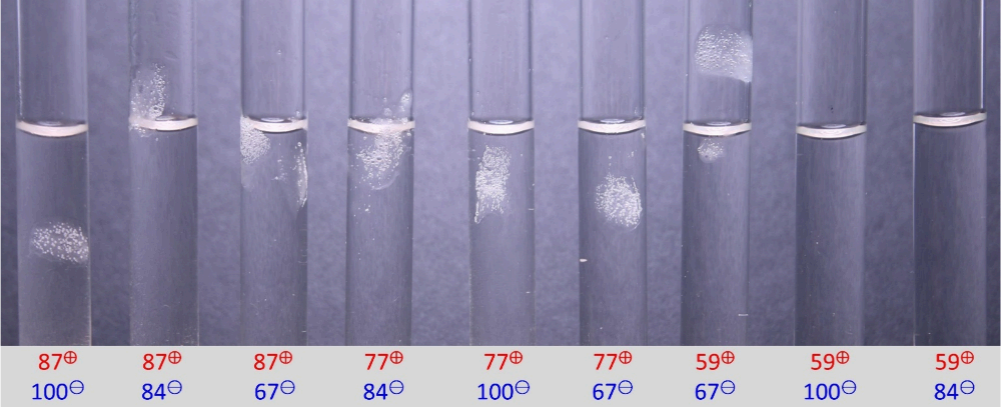
Picture series of complexes at 125 mM KBr of all nine combinations (same series that is used for NMR measurements) after equilibration overnight. Characteristic L-S phase separation is observed for the first seven complexes from 87^⊕^-100^⊖^ to 59^⊕^-67^⊖^. Mixtures 59^⊕^-100^⊖^ and 59^⊕^-84^⊖^ exhibit little complex formation via solid phase separation, as shown in the photograph in Figure S59a. The supernatant is a clear solution for all combinations.

As indicated before, the synthesized polyelectrolytes are strong complex-formers, showing a narrow coacervation window, which is in contrast to, e.g. the well-studied poly­(styrenesulfonate) (PSS) and poly­(diallyldimethylammonium) (PDADMAC) system.^21^ The strong associative phase separation also becomes evident by the rate of phase separation, which occurs instantaneously without the addition of salt, as visualized in Video S1. The combination of PSS and PDADMAC, polyelectrolytes with a rather rigid structure, requires salt as a dopant to achieve macroscopic phase separation,^8,35^ probably because small colloidal aggregates are formed that are prevented from further aggregation into macroscopic precipitates by the presence of excess charge. The polyester-based polyelectrolytes, by contrast, undergo fast and instantaneous precipitation, even at low salt concentrations or in the absence of salt. We speculate that this can be attributed to the flexible aliphatic polymer backbone. Tirrell, de Pablo, and their co-workers investigated the effect of complex coacervation on flexible and hydrophilic polyelectrolytes composed of different charge densities and observed complex coacervation without the addition of salt.^22,41^ Since complex coacervation was rarely observed with our system, we hypothesize that this may be due to the higher hydrophobicity of our polyester-based polyelectrolytes compared to the hydrophilic backbones, which exhibit denser charge packing per repeating unit. Additionally, as the charge density of the polyelectrolytes decreases, the hydrophobicity increases, which likely promotes S-L phase separation rather than complex coacervation. This may explain why complex coacervation was only observed for the PEC with the highest charge density (87^⊕^-100^⊖^). All combined, this comparison suggests that the main factors for fast macroscopic phase separation are related to the flexibility of the backbone and the overall lower charge density compared to the PSS–PDADMAC system.

At 125 mM KBr salt concentration, seven of the nine complexes showed clearly visible L-S phase separation as depicted in Figure 2. Complexes 59^⊕^-100^⊖^ and 59^⊕^-84^⊖^ also showed L-S phase separation, as determined by eye, but the yield was too low to be accurately quantified, as can be seen in Figure S56a. Interestingly, complexes 87^⊕^-67^⊖^ and 77^⊕^-67^⊖^, in which the polycation was more strongly charged, both showed high amounts of solid complex and higher salt resistance, as will be discussed below.

The composition of the complexes obtained at 125 mM KBr (Figure 2) was determined by collecting the solid complex, dissolving it in 2.5 M KBr, and then measuring the relative concentrations of the two polyelectrolytes with ^1^H NMR, according to literature procedures.^10,15,42,43^ The similarity of the polymer backbones resulted in numerous overlapping peaks of the polyanion and polycation. Selected characteristic peaks of the polyanion (*a*: corresponding to 1 proton) in blue at δ = 3.80 ppm and the polycation (*b,c*: corresponding to 3 protons) in red δ = 3.98 ppm, were used to calculate the ratio between the two polymers in the complex (Figure 3a). Overall, a close to 1:1 molar ratio of the polyelectrolytes was measured for all complexes (Figure 3b). This implies that nonstochiometric (asymmetric) complexes require the incorporation of additional counterions to compensate for the imbalance of the charges.

**Figure 3:**
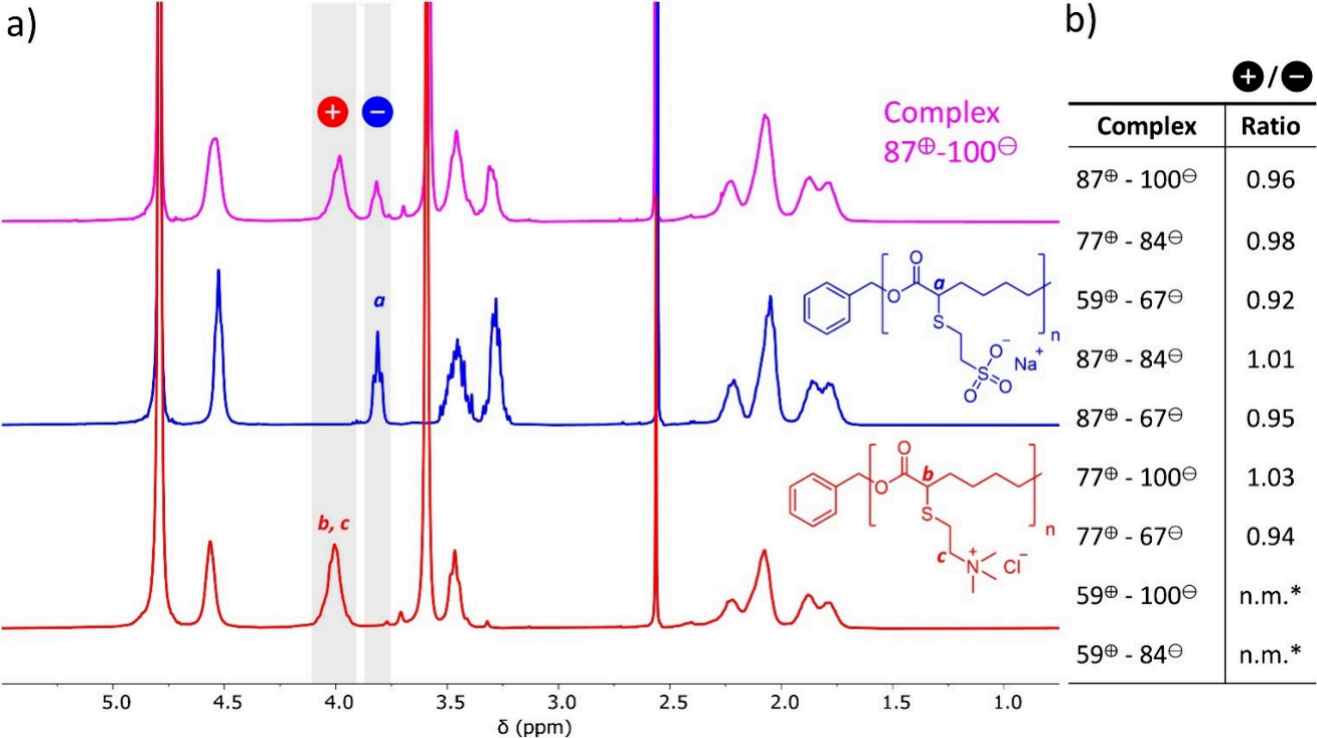
a) Overlay of ^1^H NMR spectra of P87^⊕^ (bottom, red, simplified without remaining bromine groups), P100^⊖^ (middle, blue), and complex 87^⊕^-100^⊖^ prepared at 125 mM KBr (top, pink) measured in 2.5 M KBr in D_2_O. The signals corresponding to the individual polyelectrolytes (*a* for the polyanion and *b* + *c* for the polycation) are also reflected in the ^1^H NMR spectrum of the complex 87^⊕^-100^⊖^ and labeled with ⊕ and ⊖, respectively. The integrals of ⊕ and ⊖ are used to calculate the composition of the complex. b) Summary of the molar ratio of polycation to polyanion determined by ^1^H NMR (see for calculation details the SI). *n.m (not measured), these complexes were obtained in insufficient yield to allow for characterization.

It can be concluded that the yield of complexation varied depending on the individual polyelectrolyte charge density and the salt concentration. Furthermore, even at high salt concentrations, provided they were still below the CSC, L-S phase separation was observed rather than the L-L phase separation for traditional PECs made of PSS–PDADMAC.^21^ Moreover, stoichiometric incorporation ratios based on the individual repeating units have been obtained for all nine complexes despite the differences in charge density of the individual polyelectrolytes. Existing literature presents mixed findings on the balanced inclusion of polymeric charges in the complexes. Some authors have reported a strong preference for an equal number of charges of polycation and polyanion in the complex, irrespective of the mixing ratio,^43,44^ while others have found that the charge ratio in the complex may differ substantially from unity.^42,45^ As suggested previously,^42^ these differences may be related to the nature of the complex, where liquid coacervates have a preference for a 1:1 charge ratio, while solid complexes more easily achieve an unequal charge ratio, and therefore the incorporation of additional small ions. Our findings are in agreement with this.

To further explore the sensitivity of the complexes toward ionic strength during complexation, we prepared mixtures for each of the nine combinations with varying salt concentrations. By gradually increasing the salt concentration, the complexes shifted from a phase-separated system to a one-phase system. The transition area, the CSC range, is given in Figure 4a for each complex. The lower limit indicates the highest concentration at which phase separation was still observed, and the upper limit refers to the lowest concentration at which a one-phase system was obtained. The interval steps of the CSC were limited to a range of 250 mM for complexes with a CSC higher than 1 M KBr. For combinations with lower CSC, the range was narrowed down further to 150 or 100 mM.

**Figure 4:**
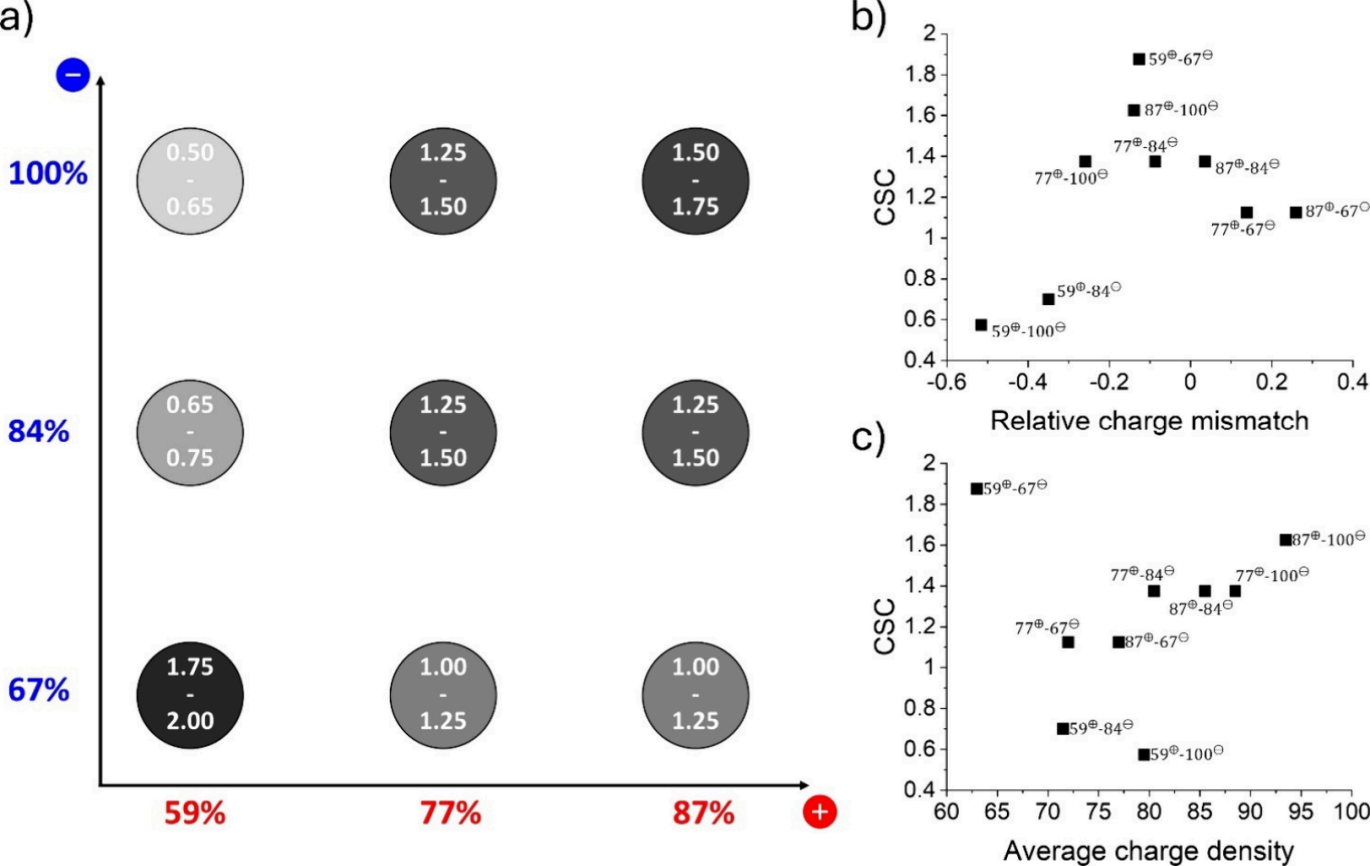
a) Overview matrix of the CSC ranges (in M KBr) of the nine different complex combinations. b) The CSCs are displayed as a function of to the relative charge mismatch, and c) average charge density of the oppositely charged polyelectrolytes. Further details can be found in Table S6.

The CSC of the nine combinations ranges from 0.5 to 2.0 M, with 59^⊕^-100^⊖^ and 59^⊕^-67^⊖^ the complexes for which the minimum and maximum value of the CSC was obtained, respectively. Overall, complexes of polyelectrolytes with similar charge densities (along a diagonal in Figure 4a) tend to have a higher CSC than complexes with a stronger charge mismatch. To further analyze this, we plot in Figure 4b the CSC as a function of the relative mismatch between the two charge densities, defined as (*f*_+_ – *f*_–_)/⟨*f*⟩, with *f*_+_ and *f*_–_ the charge density of the polycation and polyanion, respectively, and ⟨*f*⟩ = (*f*_+_ + *f*_–_)/2 the average charge density. This plot indeed shows that complexes formed by polyelectrolytes with a similar charge density are more stable than those of a larger charge mismatch. We also plot the CSC versus the average charge density in Figure 4c.

Surprisingly, the CSC is highest for the complex with the lowest charge density, 59^⊕^-67^⊖^, and varies nonmonotonically with charge density. In case the complexation would be driven by electrostatic interactions alone, a monotonically increasing trend between CSC and charge density would be expected. That this is not found indicates that other interactions, such as van der Waals or hydrophobic interactions, contribute to the complex formation as well. The lowest CSC was found for the complexes between P59^⊕^ and the highly charged polyanions (84^⊖^ and 100^⊖^). These complexes also had a very low yield, as shown in Figure 2, further underscoring the weak complexation in these mixtures. Similar trends have been reported by the groups of Laaser^29^ and Perry^40^ on aliphatic polyelectrolyte systems with varying charge densities and hydrophilicities. This nonmonotonic behavior was attributed to a balance between charge-dominated and hydrophobic regimes in the individual complexes. Despite the slight differences between the architecture of the reported polymers^29,40^ and those examined in our study, these findings confirm that the incorporation of hydrophobic groups has a strong influence on the complexation, and can lead to nonmonotonic variations with charge density. To gain a deeper insight into how the interplay between electrostatic and hydrophobic interactions determines the phase behavior, we model the binodal compositions using a simple theoretical model.

### Theoretical Considerations

Based on the experimental results, two general trends could be observed: (1) a nonmonotonic dependence of the CSC on the average charge density, with the lowest charge density corresponding to the highest CSC, and (2) a higher CSC for complexes with similar charge densities than for complexes with a large mismatch in charge density. To gain further insight into the microscopic mechanisms that could be responsible for these observations, we consider a theoretical approach, based on the classical model of Voorn and Overbeek, which has been used previously to calculate binodal lines.^34^ In this model, the free energy of a mixture of oppositely charged polymers is written as the sum of an electrostatic contribution, described in the Debye–Hückel approximation, and the Flory–Huggins expression for the mixing entropy. To account for additional nonelectrostatic interactions, we include Flory–Huggins interaction terms characterized by interaction parameters χ_ij_, leading to

$$\frac{F}{k_{B}T}=-\alpha {\left(\underset{i}{\sum }f_{i}\phi_{i}\right)}^{3/2}+\underset{i}{\sum }\frac{\phi_{i}}{N_{i}}\ln\;\phi_{i}+\underset{j<i}{\sum }\chi_{\mathrm{ij}}\phi_{i}\phi_{j}$$ 1

Equation 1 describes the free energy per unit volume in units of the thermal energy *k*_B_ *T*. The first term is the electrostatic contribution, with *f*_*i*_ and ϕ_*i*_ the charge density and volume fraction of species *i*, respectively, and α a dimensionless parameter of order unity, which characterizes the strength of the electrostatic interaction. Here, we have used α = 1.5, as previously discussed by Spruijt et al.^34^ The second term is the mixing entropy, with *N*_i_ the degree of polymerization of species *i*, and the last term accounts for the nonelectrostatic interactions between species *i* and *j*. The mixture consists of five components: the polycation *P* and polyanion *Q*, with charge densities *f*_*P*_ (0.59, 0.77, 0.87) and *f*_*Q*_ (0.67, 0.84, 1) and with *N*_*P*_ = *N*_*Q*_ = 480 (since both polymers have the same DP in our case), monovalent salt ions K^+^ and Br^–^ (*f* = 1 and *N* = 1), and water as solvent (with *f* = 0 and *N* = 1). Compressibility and electroneutrality constraints reduce the number of independent variables to three to describe the volume fraction of the five components (ϕ_*P*_, ϕ_*Q*_, ϕ_*K*_ *),* while the other two follow from the constraints. Moreover, in our experiments, we have used compositions where the polymer concentrations are equal, so ϕ_*P*_ = ϕ_*Q*_. A two-phase equilibrium is found when the total free energy of two coexisting phases differing in composition is lower than that of a single homogeneous mixture:

$$\Delta F=V_{a}F_{a}+V_{b}F_{b}$$ 2

with *V*_*a*_ and *V*_*b*_ being the volume of the two phases and *F*_*a*_ and *F*_*b*_ corresponding to their free energy densities, respectively.^34^ We use simulated annealing to numerically obtain the binodal compositions for a given initial composition.

The nonelectrostatic interactions, accounted for by the χ-parameters, which depend on the chemical nature of the different species. Here, we only consider interactions between the polymer segments and the solvent, χ_PW_ and χ_QW_; all other χ-parameters are taken equal to zero. The polymers with reduced charge density are copolymers, containing charged and uncharged monomers, which will, in general, interact differently with the solvent. Within the mean-field approximation used here, it is reasonable to assume that the overall χ-parameter is the weighted average of the charged and uncharged monomers, *i.e.:*

$$\chi_{\mathrm{PW}}=\chi_{\mathrm{PW}}^{0}\left(1-f_{P}\right)+\chi_{\mathrm{PW}}^{+}f_{P}$$ 3

$$\chi_{\mathrm{QW}}=\chi_{\mathrm{QW}}^{0}\left(1-f_{Q}\right)+\chi_{\mathrm{QW}}^{-}f_{Q}$$ 4

where χ_PW_^0^ and χ_QW_^0^ represent the interactions between the solvent and the uncharged monomers, and χ_PW_^+^ and χ_QW_^_^ those with the charged monomers. The uncharged monomers in the polyanion have propane thiol side groups, while in the polycation, there are also unreacted brominated side groups, which could make χ_PW_^0^ and χ_QW_^0^ different. Here, we ignore this difference to reduce the number of parameters and take χ_PW_^0^ = χ_QW_^0^ = 1.4. This positive value indicates nonfavorable interactions between the relatively hydrophobic monomers and water.^46^ The relatively high value is in line with our observation that the polymers become less soluble in water as the charge density is reduced.^36^ The charged groups interact favorably with water, and we therefore use negative values for χ_PW_^+^ and χ_QW_^–^. Also, here we assume that the positive and negative charges interact similarly with water and take χ_PW_^+^ = χ_QW_^–^ = – 0.6. These values imply that the solubility of both polyelectrolytes decreases upon decreasing charge density, as also observed experimentally.

We show calculated binodal lines for complexes with *f*_+_ = 0.59 in Figure 5a and for complexes with *f*_+_ = 0.87 in Figure 5b. Binodal lines for the complexes with *f*_+_ = 0.77 are shown in Figure S54. The critical salt concentration can be obtained from this as the maxima of the curves. We find that for P59^⊕^, the critical salt concentration is highest for complexes with the polyanion with the lowest charge density, while for P87^⊕^ the most stable complexes are formed with the polyanion with the highest charge density.

**Figure 5:**
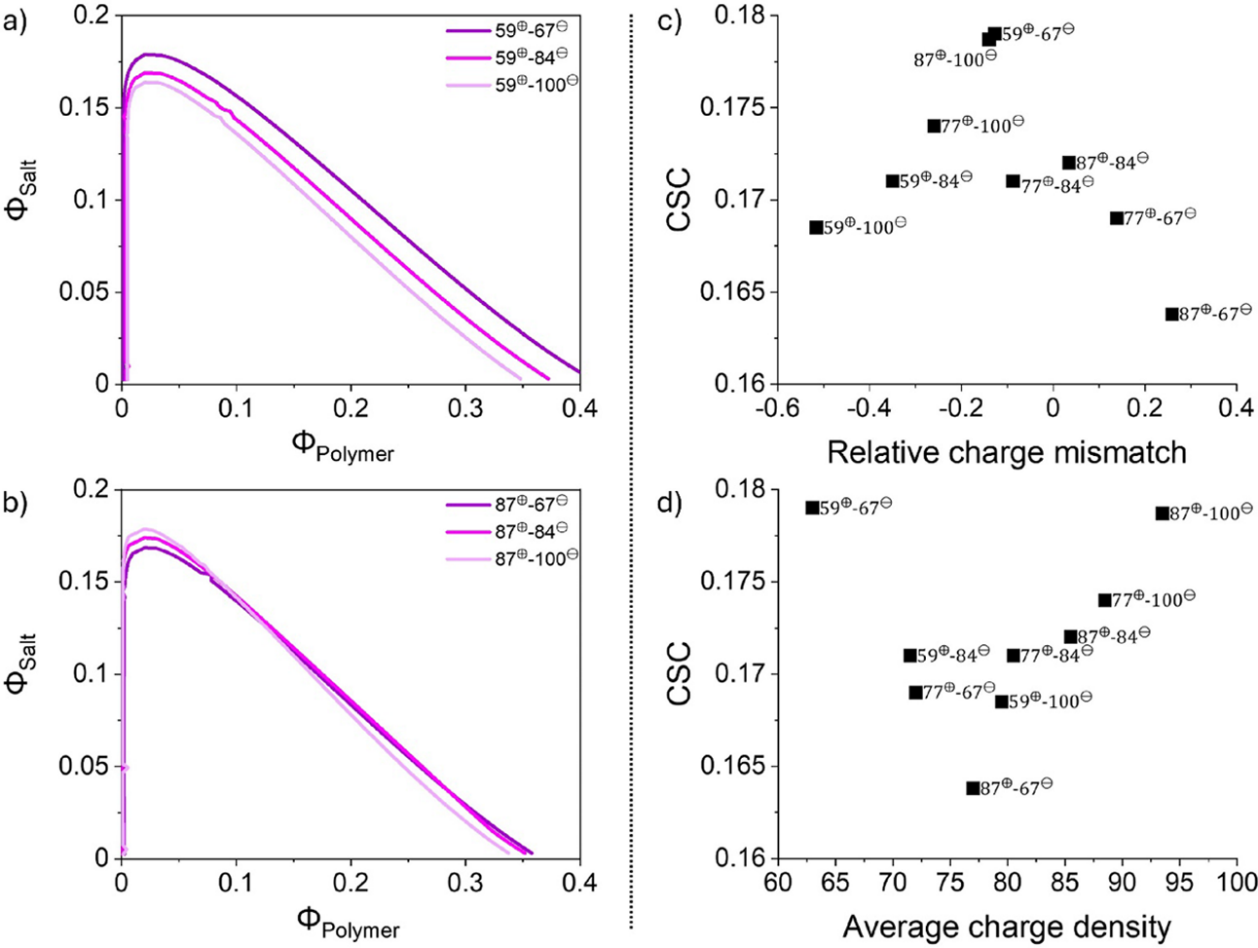
Mean-field, theoretical phase diagrams of the polyester-based polyelectrolyte complexes displaying the derived binodal lines. The mixtures of P59^⊕^ are shown in panel a), and P87^⊕^ in b). The CSCs are displayed as a function of the relative charge mismatch of the oppositely charged polyelectrolytes in c) and to the average charge density in d).

This agrees qualitatively with our experimental findings. To make a more detailed comparison, we analyze our numerical results in the same way as the experimental findings in Figure 4, and plot the CSC versus the relative charge mismatch in Figure 5c and versus the average charge density in Figure 5d. We find very similar trends as in the experimental data, notably an optimum in the stability for more stoichiometric charge ratios, and a nonmonotonic dependence as a function of the average charge density. For comparison, we have also carried out calculations in which the χ-terms are neglected, illustrated in Figure S55, so that electrostatic interactions are the only driving force. As shown in Figure S56, this leads to very different trends, where the CSC increases almost linearly with increasing charge density and is not strongly correlated with the charge mismatch. These calculations thus highlight the importance of nonelectrostatic interactions to the stability of the complexes. Indeed, the polyelectrolytes with the lowest charge density also have the most unfavorable interaction with the solvent, and this contributes significantly to the tendency to phase separate.

We note that, while the trends in the calculations and the experiments are similar, the differences in CSC between the different mixtures in the calculations are much smaller than those found experimentally. This could indicate that either additional intrinsic properties of the polyelectrolytes or external factors are not completely accounted for. The theoretical model based on the Voorn-Overbeek theory is not exact because the Debye–Hückel approximation does not account for the connectivity of the charges and for the double layers around the dissolved polyelectrolytes.^47^ More advanced models might give a more accurate description of the phase.^20,41^

A nonmonotonic trend of the CSC as a function of charge density and hydrophobicity was intensively discussed by Huang and Laaser.^23,29^ They observed similar behavior for stoichiometric mixtures of polyelectrolytes consisting of an alkyl side chain with a length of either C_2_ or C_4_ as a function of charge density. For longer alkyl chains, this nonmonotonic behavior was not observed. Furthermore, they observed that solubility is not a precise indicator of the CSC. They hypothesized that this behavior is mainly governed by entropic forces that arise from the release of the counterions upon the formation of ionic bonds as well as the release of water due to hydrophobic aggregation. Depending on the charge density of the polyelectrolytes, the phase behavior is dominated by one of these factors, namely, at high charge density by the release of the counterions and at low charge density by hydrophobic interactions.

## 4 Conclusions

We describe the complexation behavior of polyester-based polycations and polyanions with a controlled charge density obtained via the controlled nucleophilic substitution of bromine atoms on a brominated poly-ε-caprolactone backbone with charged moieties. This approach allowed the control of the molecular architecture of both complex components and tuning of the resulting material properties. Charge density and hydrophobicity were found to play a crucial role in both the complexation behavior and the CSC of the complex. In particular, the high CSC of the complex with the lowest charge density shows that charge is not the only key parameter and suggests that hydrophobic interactions play an important role. Despite the differences in charge density, all complexes have been obtained in a nearly 1:1 molar ratio, suggesting a preferred composition within the complex. Inclusion of nonelectrostatic interactions as a function of the polyelectrolyte charge density in mean-field modeling was found to be useful in the interpretation of some of the complexation results, especially on the nonmonotonic dependence of the CSC on the average charge density and the higher stability of complexes with similar charge densities. The experimental findings and theoretical support are believed to support the direction of expansion of the library of synthetic polyelectrolytes toward the design of highly functional materials. While designed within the context of recyclable and degradable plastics, further analysis of the mechanical and thermal properties of the new PECs is expected to aid in exploring potential alternative applications for this class of materials.

## Supplementary Material





## Notes

Polymer synthesis; complexationoverview and turbidity measurements; ^1^H NMR data; ^13^C NMR data; DOSY NMR data; size exclusionchromatography (SEC) data; TGA data; DSC data; theoretical considerations; and photographs (PDF)
Video showing the rate of phase separation (MP4)

## Notes

DEMCON QL Polymers, Institutenweg 40, 7521 PK Enschede, The Netherlands
