# Supplementary material for: Synthesis and Complexation Behavior of Well-Defined Polyester-Based Polyelectrolytes with Varying Charge Densities and Hydrophobicities
Source: Macromolecules. 2025 Nov 21;58(23):12825–35. doi: 10.1021/acs.macromol.5c01795 (PMC12874641; doi:10.1021/acs.macromol.5c01795)
Supplement: Supplementary file 1 [file ma5c01795_si_001.pdf]

Supporting Information

Synthesis and Complexation Behavior of well-defined Polyester-based Polyelectrolytes with varying Charge Densities and Hydrophobicities

Julian Engelhardt[1,2,3], Louis C.P.M. de Smet[1], Evelien Maaskant[2], and Jasper van der Gucht[3*]*

[1] Laboratory of Organic Chemistry, Wageningen University, Stippeneng 4, 6708 WE Wageningen, The Netherlands

[2] Wageningen Food and Biobased Research, Bornse Weilanden 9, 6708 WG, Wageningen, The Netherlands

[3] Laboratory of Physical Chemistry and Soft Matter, Wageningen University, Stippeneng 4, 6708 WE Wageningen, The Netherlands

* To whom correspondence should be addressed:

E-Mail:jasper.vandergucht@wur.nl, louis.desmet@wur.nl

Contents

1. Polymer synthesis 3

2. Complexation overview and turbidity measurements 6

3. ^{1}H-NMR data 8

3.1. Monomer synthesis 8

3.2. Polymer synthesis 10

3.3. Post functionalization 12

3.4. Polyanion 15

3.5. Polycation 18

3.6. Polyelectrolyte complexes 21

4. ^{13}C-NMR data 27

5. DOSY NMR data 32

6. Size Exclusion Chromatography (SEC) data 38

7. TGA data 39

8. DSC data 43

9. Theoretical considerations 47

10. Photographs 49

1. Polymer synthesis

Table S 1: Overview of the amounts of reagents used and yields of the synthesis of the polyelectrolytes with reduced charge density from the partly functionalized neutral polymers P(αBrCL)-(84%) and P(αBrCL)-(67%).

**Polyelectrolyte**	**Neutral polymer**	**Thiol***	**Triethylamine**	**Yield**
P77⊕	4.5 g, 23.4 mmol	7.5 g, 45.7 mmol	6.4 mL, 45.7 mmol	4.0 g (65 %)
P84⊖	4.5 g, 23.4 mmol	6.6 g, 46.6 mmol	6.4 mL, 46.6 mmol	4.6 g (74 %)
P59⊕	4.2 g, 21.8 mmol	5.5 g, 39.1 mmol	5.5 mL, 39.1 mmol	3.7 g (68 %)
P67⊖	4.2 g, 21.8 mmol	5.5 g, 39.1 mmol	5.5 mL, 39.1 mmol	4.2 g (77 %)

*The thiol used for the polycation synthesis was 2-(dimethylamino) ethanethiol hydrochloride, and for the polyanion it was sodium 2-mercaptoethanesulfonate.

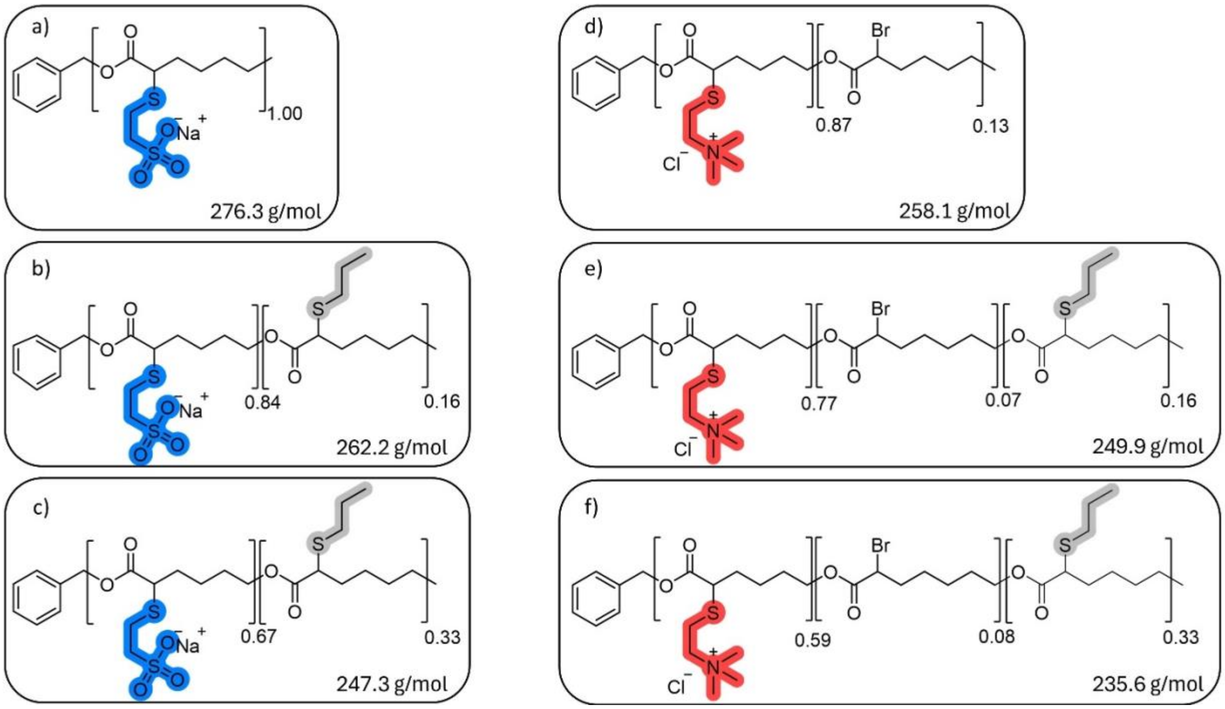

Scheme S 1: Overview of six polyelectrolytes with their corresponding charge densities (a: 100%, b: 84%, c: 67%) as polyanion (blue) and (d: 87%, e: 77%, f: 59%) polycation (red). For each polymer, the average molecular weight of the repeating units, based on the ratio of charged and neutral repeating units, is given.

The average molecular weight of the polymers containing reduced charge density (16% and 33% alkyl chain content) was calculated by the sum of the ratios of the molecular weight of the different repeating units. The anionic repeating unit and the cationic repeating unit have molecular weight of 276.3 g/mol and 267.8 g/mol, respectively. The molecular weight of the non-charged repeating unit is 188.3 g/mol, and the molecular weight of the non-functionalized bromine unit is 193.0 g/mol. The average molecular weight of the repeating units for the polyelectrolytes with reduced charge density is calculated by this formula:

$$M_{\bar{x}} = Mw^{+}_{-} \times f^{+}_{-} + Mw_{\emptyset} \times f_{\emptyset} + Mw_{Br} \times f_{\mathrm{Br}} \qquad S1$$

where $M_{\bar{x}}$ represents the calculated average molecular weight of the polyelectrolyte, Mw^{+}_{-} is the molecular weight of either the anionic or cationic repeating unit, and f^{+}_{-} the corresponding fraction

of this repeating unit in the polymer backbone. The molecular weight of the non-charged group is $M_{w_\emptyset}$ and its fraction $f_\emptyset$ represents the congruous functionalization degree (33% or 16%). For the polycations, the substitution of the bromine was incomplete. Thus, the molecular weight is composed of a third fraction: the molecular weight of the bromine repeating unit by Mw_{Br} and the fraction of is given by f_{Br}. (e.g. M_w of P77⊕: 267.8 g/mol × 0.84 + 188.3 g/mol × 0.16 + 193.04 × 0.07 = 249.9 g/mol).

2. Complexation overview and turbidity measurements

Table S 2: Overview of the formulations for altering the salt concentration of the complexation experiments. The amounts of KBr, Milli-Q water, and polyelectrolyte stock solutions are given in mL.

**Target salt concentration (× 10 mM)**	2	12.5	25	50	65	75	100	125	150	175	200
**KBr stock solution (4 M) (mL)**	0	0.053	0.115	0.240	0.315	0.365	0.490	0.615	0.740	0.865	0.990
**Milli-Q water (mL)**	1.200	1.150	1.090	0.960	0.890	0.840	0.710	0.590	0.460	0.340	0.210
**Polyelectrolytes stock solution (50 mM) (mL)**	0.800	0.800	0.800	0.800	0.800	0.800	0.800	0.800	0.800	0.800	0.800

Table S 3: Overview of the phase separation of the nine complexes by increasing salt concentration. The observed phase separation was indicated: blue (solid-liquid), purple (liquid-liquid), and light blue (one phase). The salt concentration is displayed in mM. The values are the measured absorbance of the supernatant phase.

**Entry**	**Absorbance ($*10^{-2}$) at λ: 500nm**										
KBr concentration (mM)	**20**	**125**	**250**	**500**	**650**	**750**	**1000**	**1250**	**1500**	**1750**	**2000**
87⊕-100⊖	4.9	0.4	1.2	1.2		0.3	0.1	0.4	0.5	0.7	1.2
77⊕-84⊖	1.1	0.3	0.6	0.6	-	0.5	1.0	1.6	0.5	-	0.4
59⊕-67⊖	3.4	0.4	0.5	0.5	-	0.5	0.6	0.7	0.5	0.6	0.4
87⊕-84⊖	15.1	0.8	0.4	0.4	-	0.5	0.7	0.4	0.7	1.5	1.7
87⊕-67⊖	2.3	0.8	0.6	0.6	-	1.0	0.7	13.0	2.1	-	1.5
77⊕-100⊖	10.6	0.6	0.6	0.6	-	1.1	1.6	3.0	0.6	-	0.5
77⊕-67⊖	2.0	0.5	0.9	0.9	-	0.9	0.7	2.3	0.6	-	0.4
59⊕-100⊖	1.4	0.6	0.6	0.6	1.3	0.8	0.8	-	0.8	-	0.5
59⊕-84⊖	0.9	0.4	1.2	0.8	1.1	0.7	0.6	-	0.6	-	0.4

3. ^{1}H-NMR data

3.1.Monomer synthesis

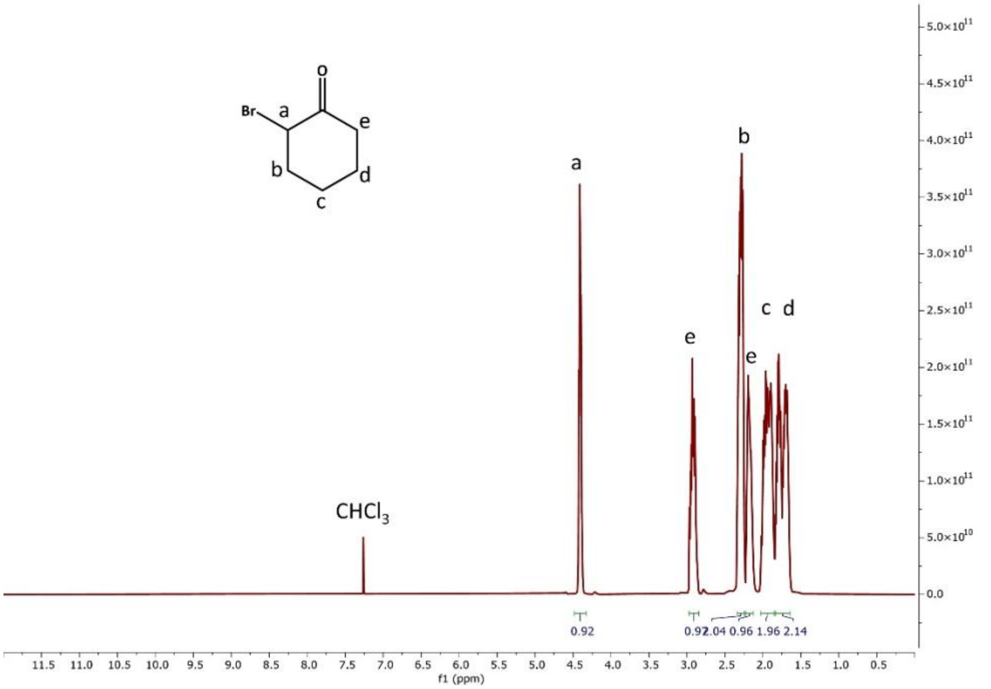

Figure S 1: ^{1}H-NMR spectrum (400 MHz, $CDCl_3$) of α-bromo-cyclohexanone: δ 1.64 – 2.03 (m, 4H, $–CH_2CH_2–$), 2.13 – 2.23 (m, 1H, $COCH_2$) , 2.30 (m, 2H, $–CH_2–$), 2.89-2.93 (m, 1H, $COCH_2$), 4.40-4.42 (t, 1H, CH–Br).

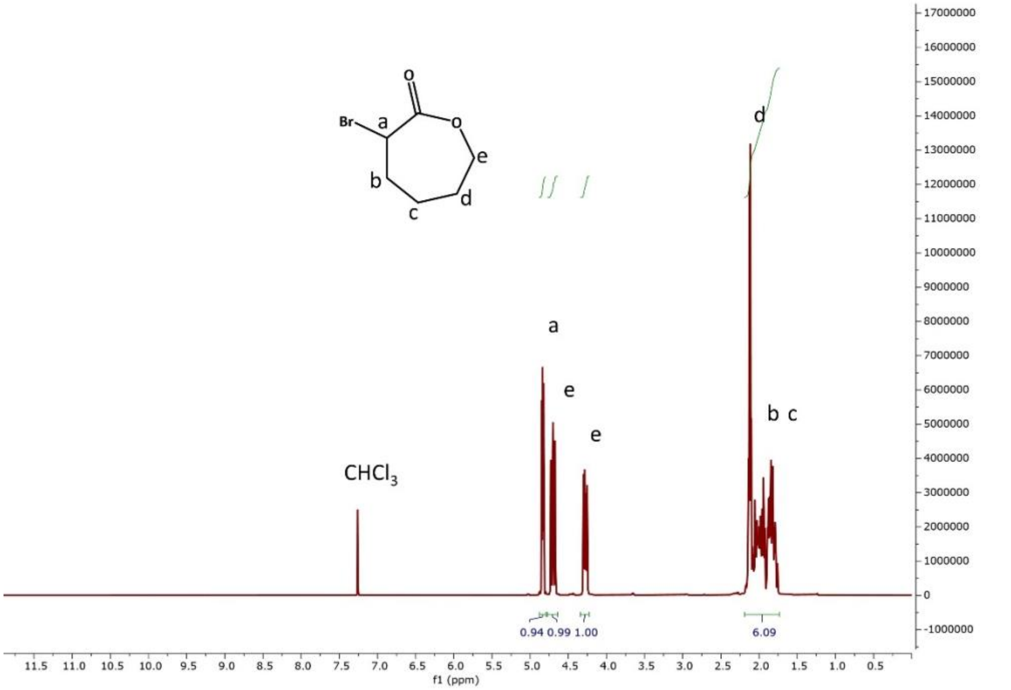

Figure S 2:^{1}H-NMR spectrum (400 MHz, $CDCl_3$) of α-bromo-ε-caprolactone: δ 2.11 (m, 6H, –CH_2CH_2CH_2–), 4.28-4.71 (m, 2H, –COOCH_2–), 4.85 (t, 1H, –CH(Br)–).

3.2.Polymer synthesis

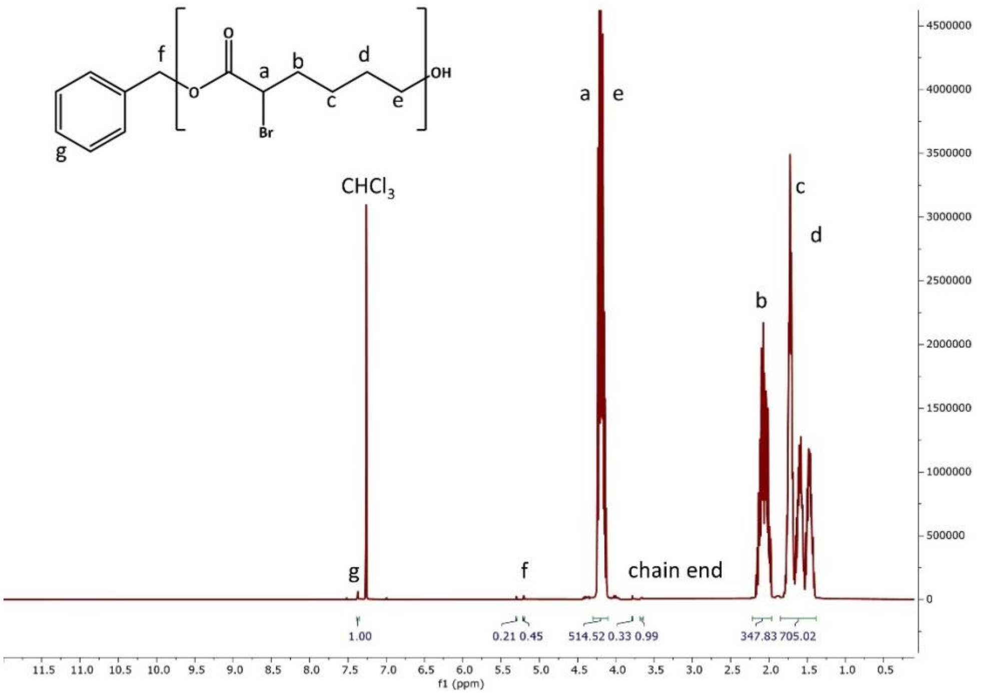

Figure S 3: ^{1}H-NMR spectrum (400 MHz, $CDCl_3$) of P(αBrCL): δ 1.85 – 1.39 (m, 4H,–C*H*2C*H*2–backbone), 2.22 – 1.96 (m, 2H, BrC*H*2), 3.63 – 3.69(m, 2H, –C*H*2Ochain end), 4.30 – 4.10 (m, 3H, C*H*–Br and COC*H*2), 5.20 (s, 2H, C*H*2Obenzyl protons), 7.36–7.39 (m, 5H, Ar–H).

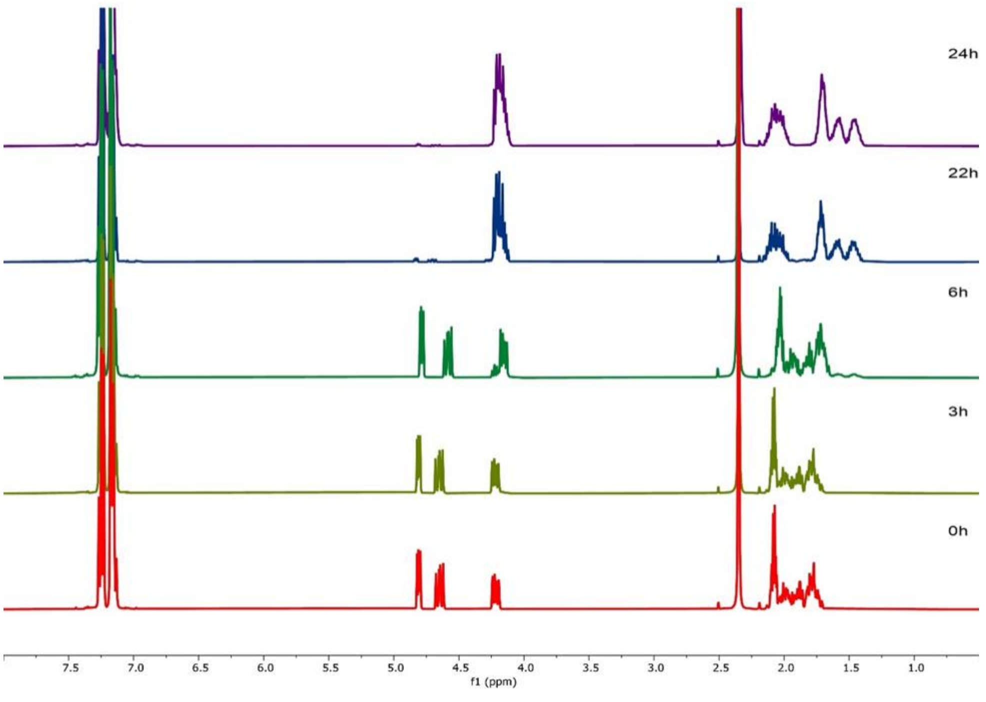

Figure S 4: Stacked ^{1}H-NMR spectra for the time-dependent ring-opening polymerization of αBrCL in $CDCl_3$.

3.3.Post functionalization

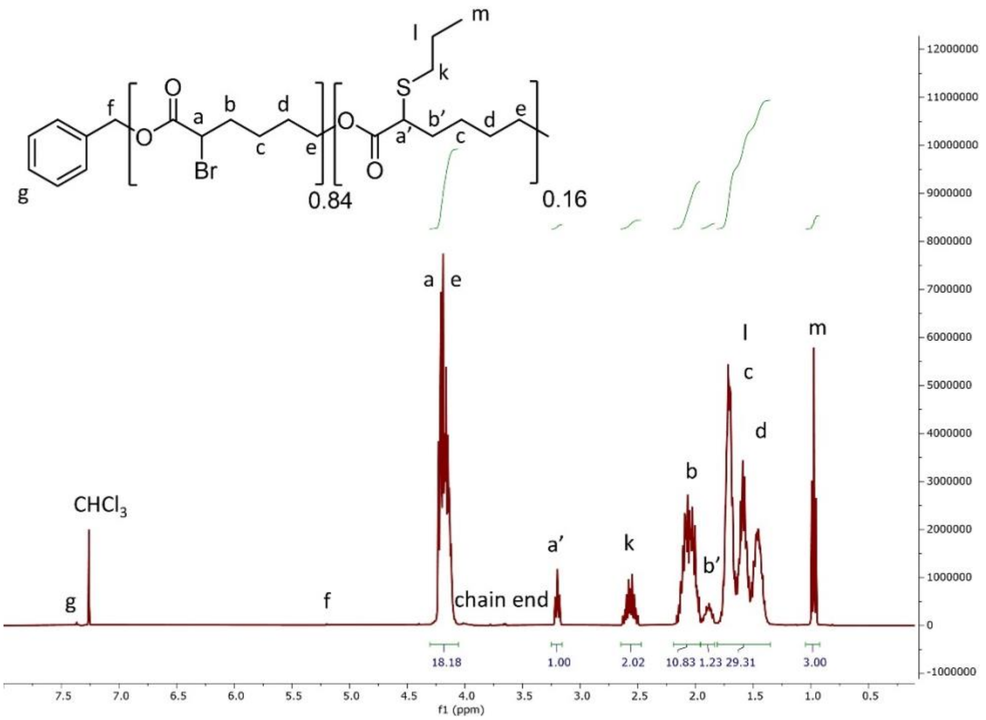

Figure S 5: ^{1}H-NMR spectrum (400 MHz, $CDCl_3$) of P(αBrCL)(84%): *δ* 4.25 – 4.00 (m, 18H), 3.14 (t, 1H), 2.51 (m, 2H), 2.13 – 1.90 (m, 11H), 1.90 – 1.77 (m, 1H), 1.75 – 1.29 (m, 30H), 0.91 (t, 3H).

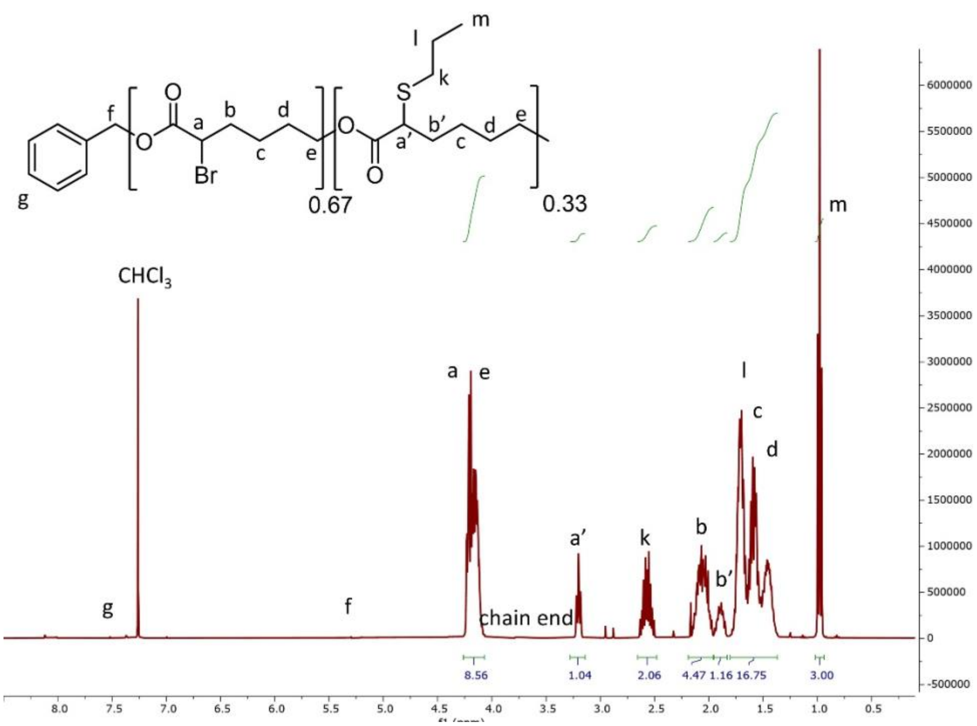

Figure S 6: ^{1}H-NMR spectrum (400 MHz, $CDCl_3$) of P(αBrCL)(67%): δ 4.20 – 4.02 (m, 9H), 3.14 (t, 1H), 2.51 (m, 2H), 2.13 – 1.76 (m, 6H), 1.39 (s, 17H), 0.91 (t, 3H).

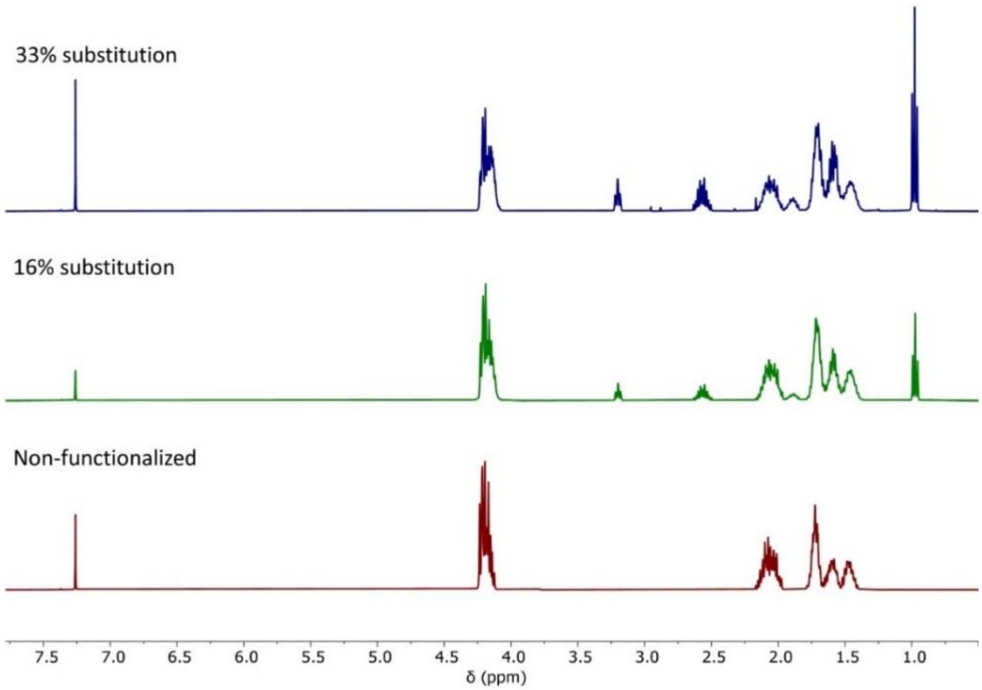

Figure S 7: Stacked ^{1}H-NMR spectra of non-charged P(αBrCL), and the partly functionalized P(αBrCL)-(84%) and (67%) in $CDCl_3$.

The degree of functionalization was calculated by the ratio of the signal at 3.1 and 4.2 ppm, which corresponds to the α-proton (bridgehead; 1H) and the ε-protons (2H). The non-functionalized P(αBrCL) shows no signal at 3.1 ppm. This signal only appears after the partial substitution with the propane thiol. Consequently, the change in the integral value of the signal at 4.2 ppm can be used to calculate the degree of functionalization. For example, P(αBrCL)-(84%): the integral value of 4.2 ppm is 18.10 and that of 3.1 ppm is 1.00. The sum of both integrals (19.10) corresponds to three protons. Therefore, the integral value of one proton corresponds to 19.10/3 = 6.36. The value of the integral at 3.10 indicates the degree of functionalization compared to the non-functionalized α-proton at 4.2. Hence the ratio of 1.00/6.36 = 0.16 indicates, that a functionalization of 16% was achieved. Thus, 84% of the bromine remains unsubstituted, and can be functionalized into a charged moiety. In a similar manner, the degree of functionalization of P(αBrCL)-(67%) was calculated.

3.4.Polyanion

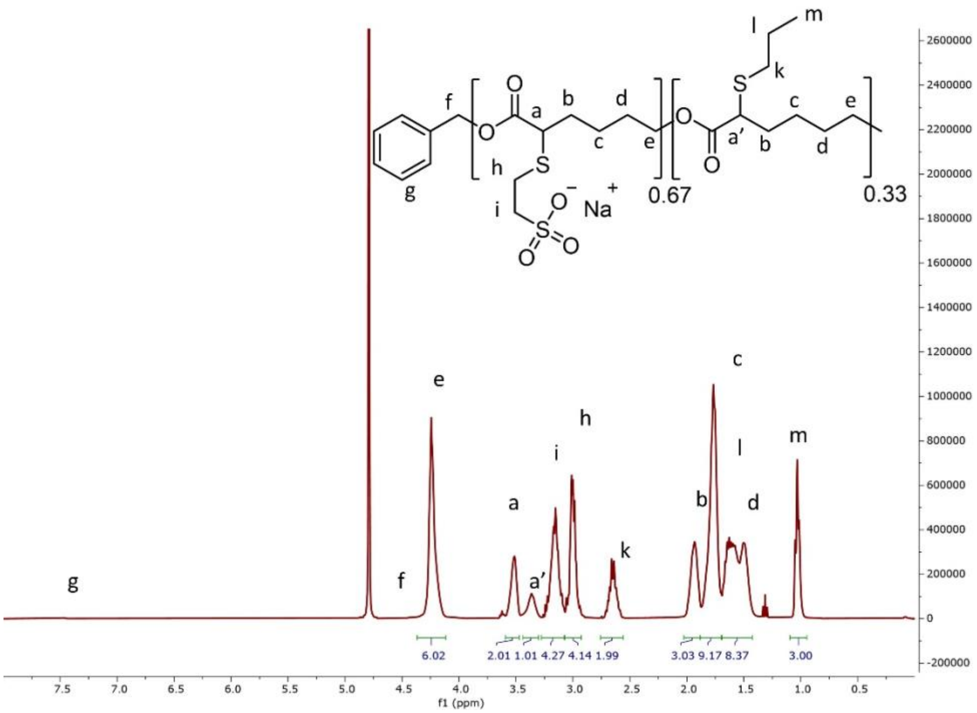

Figure S 8: ^{1}H-NMR spectrum of P67⊖ (400 MHz, D_2O): δ 0.95 – 1.10 (m, 3H), 1.43 – 1.69 (m, 4H), 1.70 – 1.88 (m, 3H), 1.88 – 2.03 (m, 1H), 2.56 – 2.76 (m, 2H), 2.93 – 3.07 (m, 2H), 3.08 – 3.28 (m, 2H), 3.29 – 3.41 and 3.47 – 3.59 (m, 1H), 4.12 – 4.37 (m, 2H).

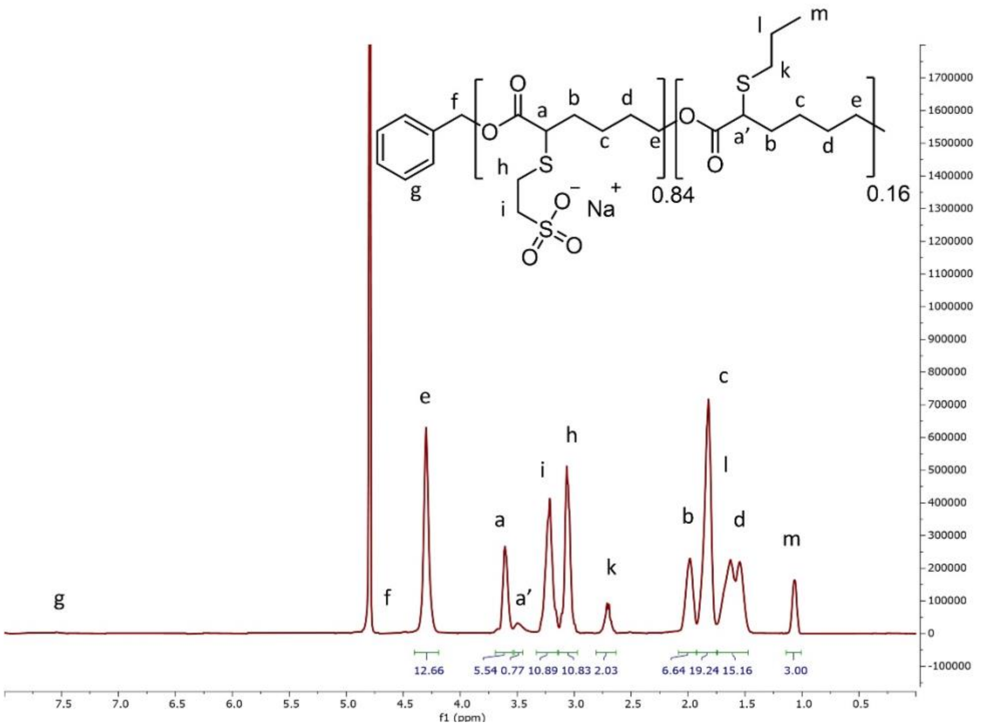

Figure S 9: ^{1}H-NMR spectrum of P84⊖(400 MHz, D_2O): δ 1.02 – 1.10 (m, 3H), 1.59 (m, 2H), 1.75 – 1.92 (m, 3H), 1.99 (s, 1H), 2.65-2.75 (m, 2H) 3.06 (m, 2H), 3.21 (m, 2H), 3.42-3.53 and 3.54 – 3.69 (m, 1H), 4.19 – 4.40 (m, 2H).

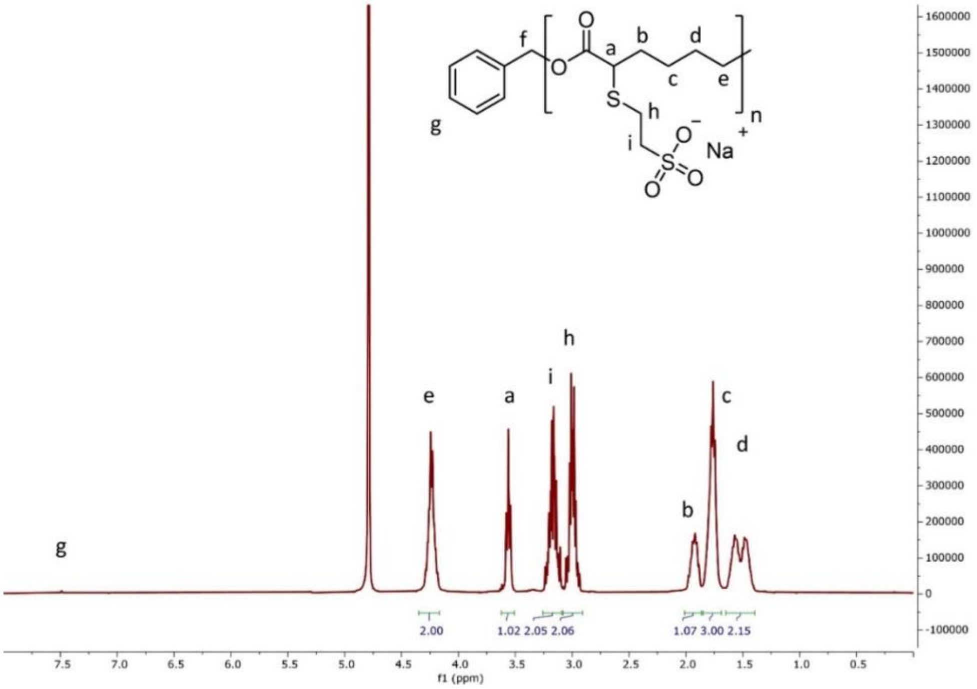

Figure S 10: ^{1}H-NMR spectrum of P100⊖ (400 MHz, D_2O): δ 1.39 – 1.65 (m, 2H), 1.69 – 1.85 (m, 3H), 1.86 – 2.01 (m, 1H), 2.91 – 3.08 (m, 2H), 3.09 – 3.26 (m, 2H), 3.50 – 3.64 (m, 1H), 4.17 – 4.35 (m, 2H).

3.5.Polycation

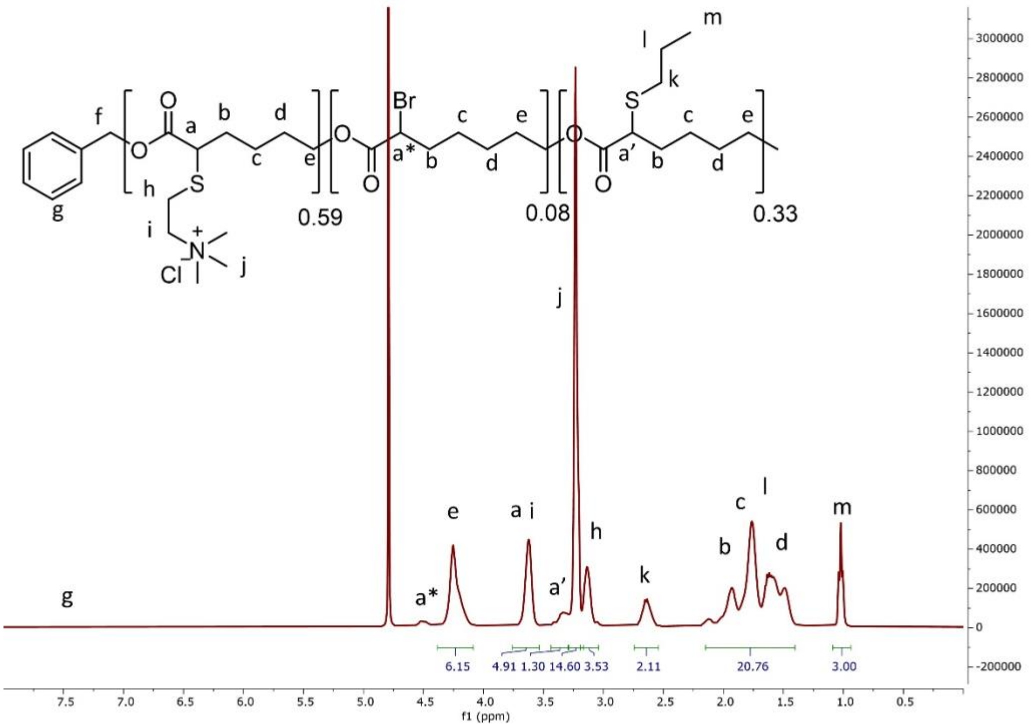

Figure S 11: ^{1}H-NMR spectrum of P59⊕ (400 MHz, D_2O): δ 0.85 – 1.00 (m, 3H), 1.29 – 1.98 (m, 7H), 2.45 – 2.66 (m, 1H), 2.95 – 3.10 (m, 1H), 3.08 – 3.21 (m, 5H), 3.45 – 3.67 (m, 2H), 4.00 – 4.29 (m, 2H).

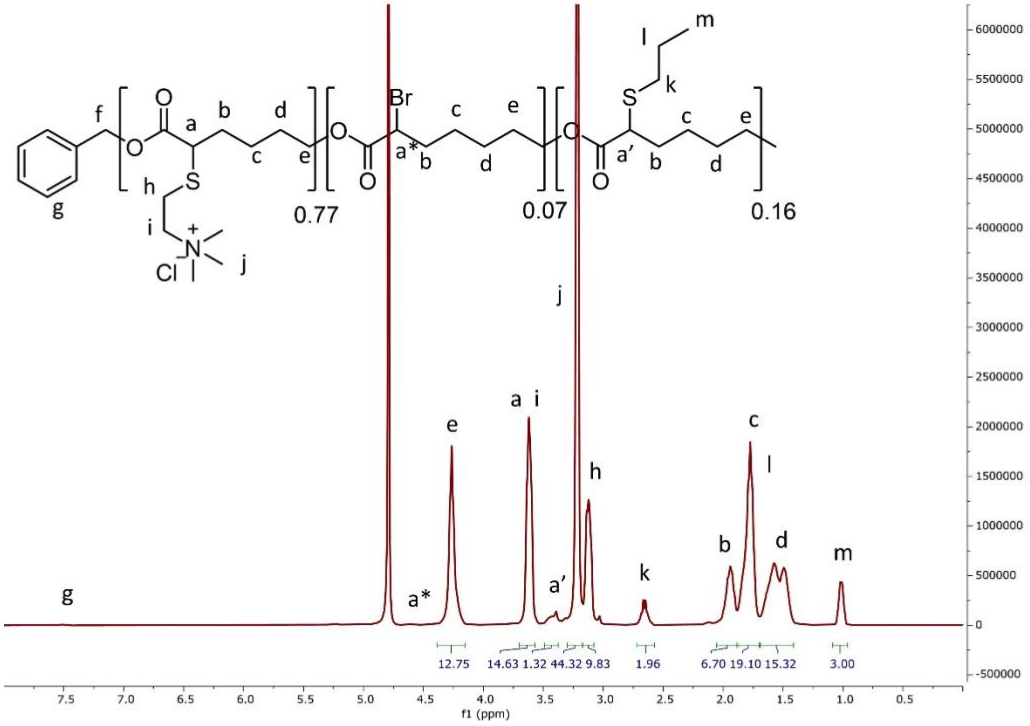

Figure S 12: ^{1}H-NMR spectrum of P77⊕ (400 MHz, D_2O): δ 0.97 – 1.06 (m, 3H), 1.41 – 1.70 (m, 4H), 1.69 – 1.89 (m, 3H), 1.88 – 2.06 (m, 1H), 2.61 – 2.69 (m, 2H), 3.08 – 3.17 (m, 2H), 3.17 – 3.30 (m, 9H), 3.35 – 3.47 and 3.57 – 3.70 (m, 1H), 4.15 – 4.39 (m, 2H).

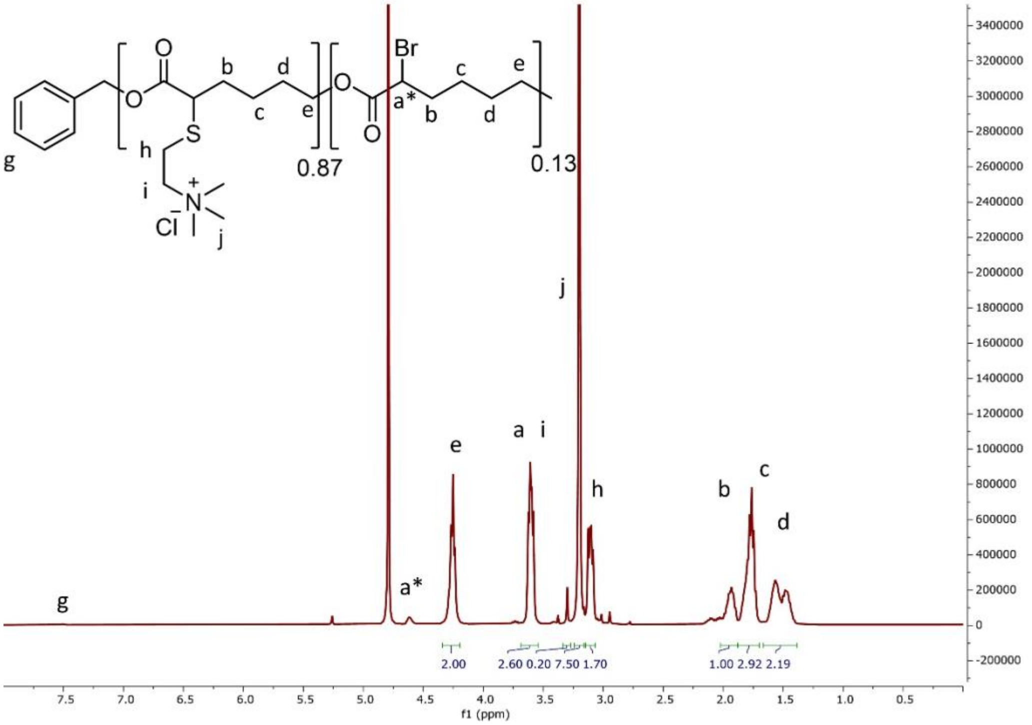

Figure S 13: ^{1}H-NMR spectrum of P87⊕ (400 MHz, D_2O): δ 1.39 – 1.67 (m, 2H), 1.70 – 1.88 (m, 3H), 1.88 – 2.02 (m, 1H), 3.07 – 3.15 (m, 2H), 3.16 – 3.24 (m, 9H), 3.54 – 3.69 (m, 3H), 4.20 – 4.34 (m, 2H).

3.6.Polyelectrolyte complexes

The composition of the complexes was calculated by ^{1}H-NMR. Characteristic and isolated signals of the polyanion and polycation were used to calculate the ratio of the integrals which can be correlated to the molar ratio of the two polyelectrolytes in the complex. For the polyanions, the signal at ~3.55 ppm corresponding to the bridgehead proton of the anion-bearing group was used (***a***, in **Figure S 14**). For the polycation, the signal at ~3.65 ppm corresponds to three protons, namely the bridgehead proton of the cation-bearing group and two protons of the methylene group adjacent to the quaternary ammonium salt (***b*** and ***c*** in **Figure S 14**), was used.

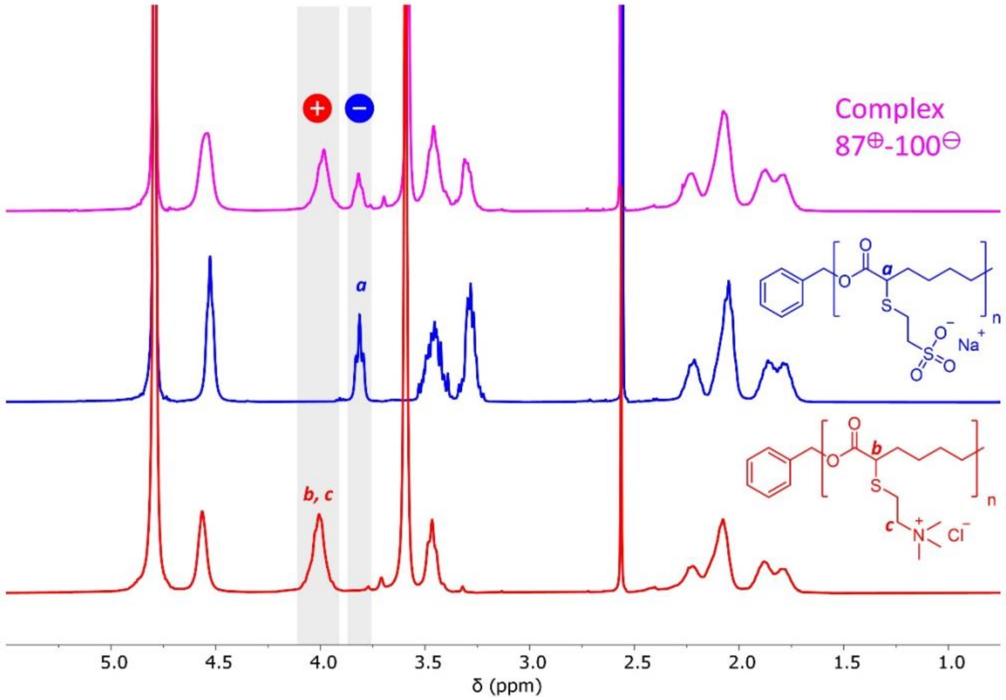

Figure S 14: Overlay of ^{1}H-NMR spectra of P87⊕ (bottom, red, simplified without remaining bromine groups), P100⊖ (middle, blue), and complex 87⊕-100⊖ prepared at 125 mM KBr (top, pink) measured in 2.5 M KBr in D_2O. The signals corresponding to the individual polyelectrolytes (***a*** for the polyanion and ***b*** + ***c*** for the polycation) are also reflected in the ^{1}H-NMR spectrum of the complex 87⊕-100⊖ and labelled with ⊕ and ⊖, respectively. The integrals of the peaks labelled with ⊕ and ⊖ are used to calculate the composition of the complex (see **Figure 4**).

For each polyelectrolyte, the theoretical number of protons (n_{theo}) for signal ***a*** or ***b+c*** was calculated based on the targeted charge density and the actual conversion of the substitution of the bromine with the charged thiol. The results are shown in **Table S 4.**

Table S 4: Overview of the molar fraction of charged groups and the corresponding theoretical amount of protons for signals ***a*** or ***b+c*** as defined in **Figure S 14** for all polyelectrolytes.

Polyelectrolyte	Molar fraction of charged groups (%)	n_{theo}
P100⊖	100	1
P84⊖	84	0.84
P67⊖	67	0.67
P87⊕	87	2.61
P77⊕	77	2.31
P59⊕	59	1.77

Based on n_{theo} and the measured value of the integral I, the normalized integral I_{norm} was calculated for both the polyanion and polycation in the polyelectrolyte complex using:

$$I_{\text{anion,norm}} = \frac{I_{\text{anion,a}}}{n_{\text{theo,a}}}, I_{\text{cation,norm}} = \frac{I_{\text{cation,b+c}}}{n_{\text{theo,b+c}}}$$

From these normalized integrals, the molar ratio of the polycation to polyanion R_{CA} in the polyelectrolyte complex was calculated using:

$$R_{\text{CA}} = \frac{I_{\text{cation,norm}}}{I_{\text{anion,norm}}}$$

A value above 1 indicates an excess of polycation in the complex and vice-versa. **Table S 5** shows, for all polyelectrolyte complexes, the value of the integrals, the normalized integrals and the molar ratio of polycation to polyanion.

Table S 5: Overview of the measured integrals (I), the normalized integrals (I_{norm}) and ratio of polycation to polyanion (R_{CA}) in the complex.

Complex	I_{anion}	I_{cation}	$I_{anion,norm}$	$I_{cation,norm}$	R_{CA}
$87^{\oplus}$ / $100^{\ominus}$	0.40	1	0.40	0.38	0.96
$77^{\oplus}$ / $84^{\ominus}$	0.37	1	0.44	0.43	0.98
$59^{\oplus}$ / $67^{\ominus}$	0.41	1	0.61	0.56	0.92
$87^{\oplus}$ / $84^{\ominus}$	0.32	1	0.38	0.38	1.01
$87^{\oplus}$ / $67^{\ominus}$	0.27	1	0.40	0.38	0.95
$77^{\oplus}$ / $100^{\ominus}$	0.42	1	0.42	0.43	1.03
$77^{\oplus}$ / $67^{\ominus}$	0.31	1	0.46	0.43	0.94

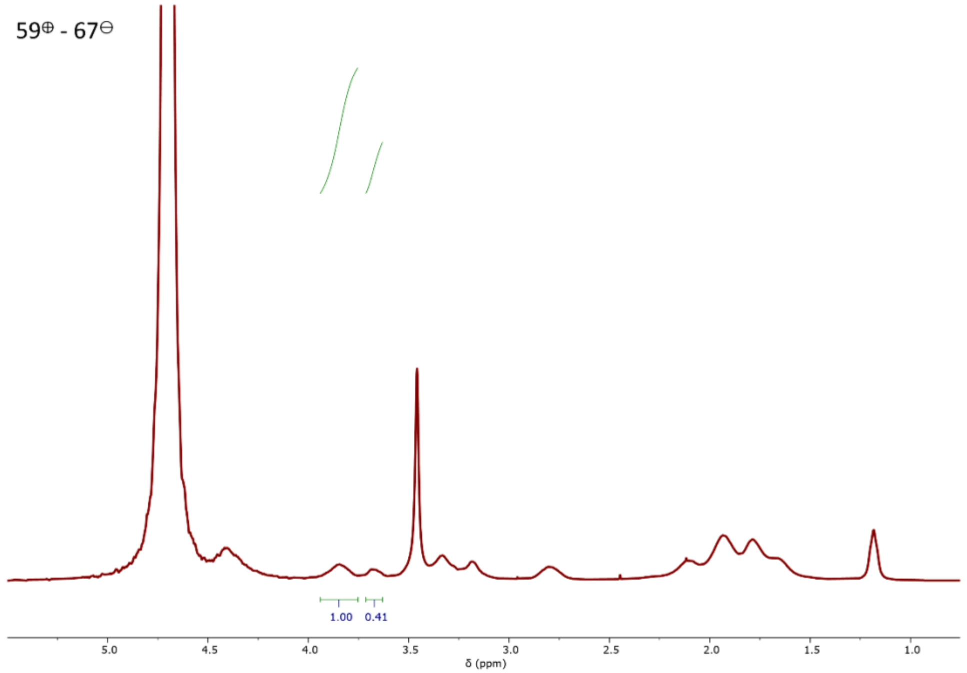

Figure S 15: ^{1}H-NMR spectrum (400 MHz, 2.5 M KBr in D_2O) of $59^{\oplus}$-$67^{\ominus}$.

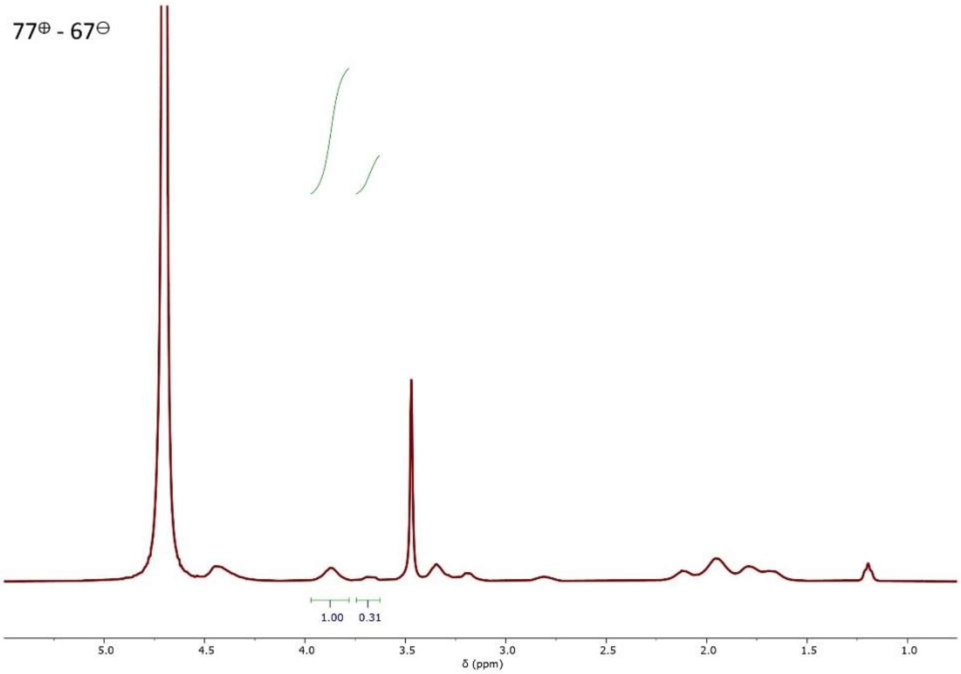

Figure S 16: ^{1}H-NMR spectrum (400 MHz, 2.5 M KBr D_2O) of 77⊕-67⊖.

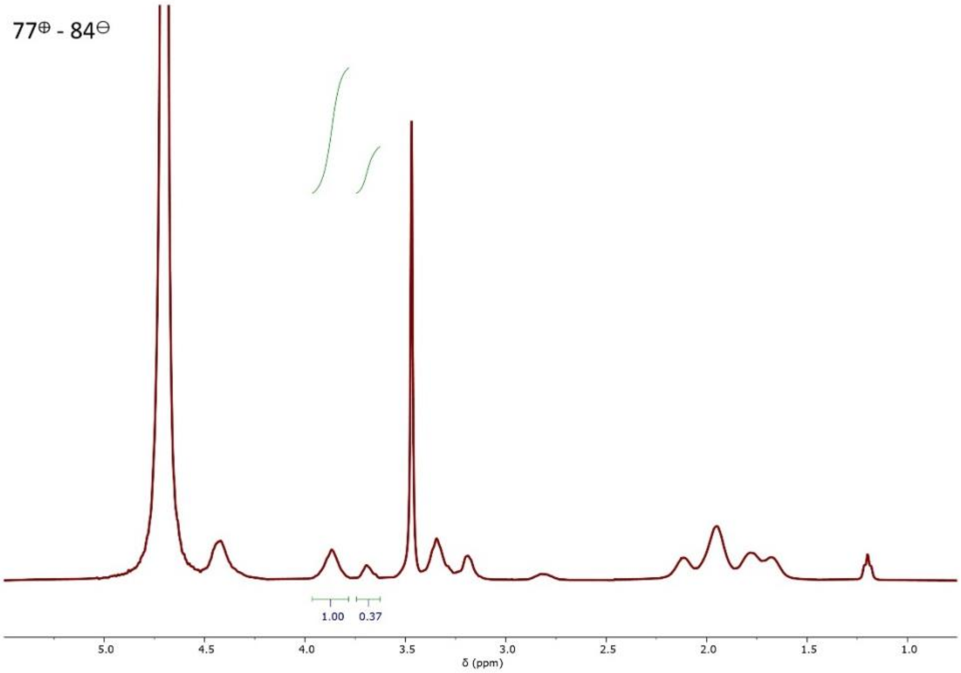

Figure S 17: ^{1}H-NMR spectrum (400 MHz, 2.5 M KBr D_2O) of 77⊕-84⊖.

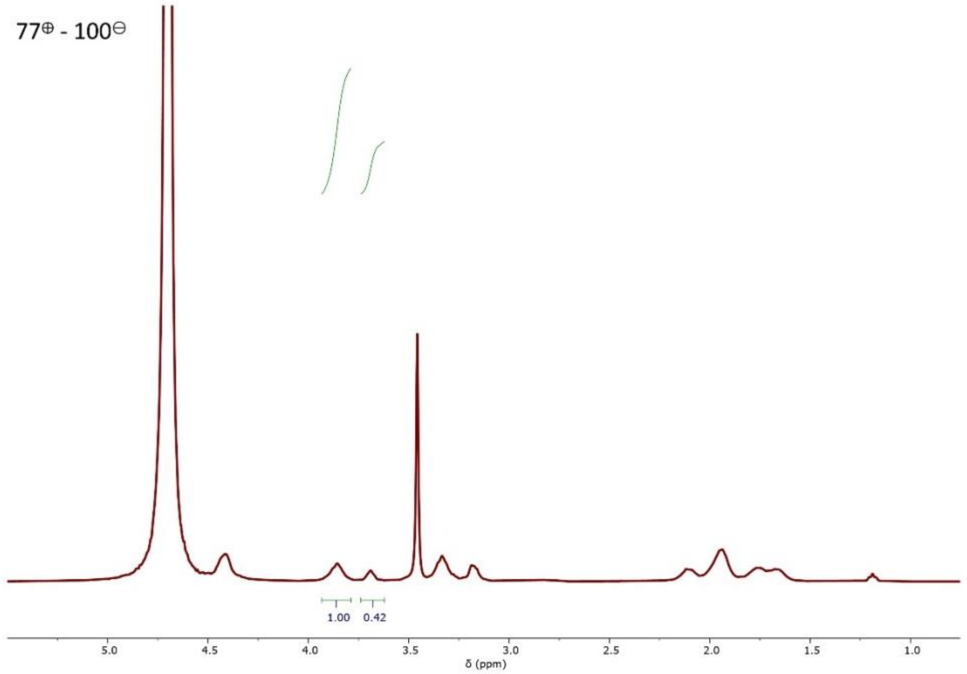

Figure S 18: ^{1}H-NMR spectrum (400 MHz, 2.5 M KBr D_2O) of 77⊕-100⊖.

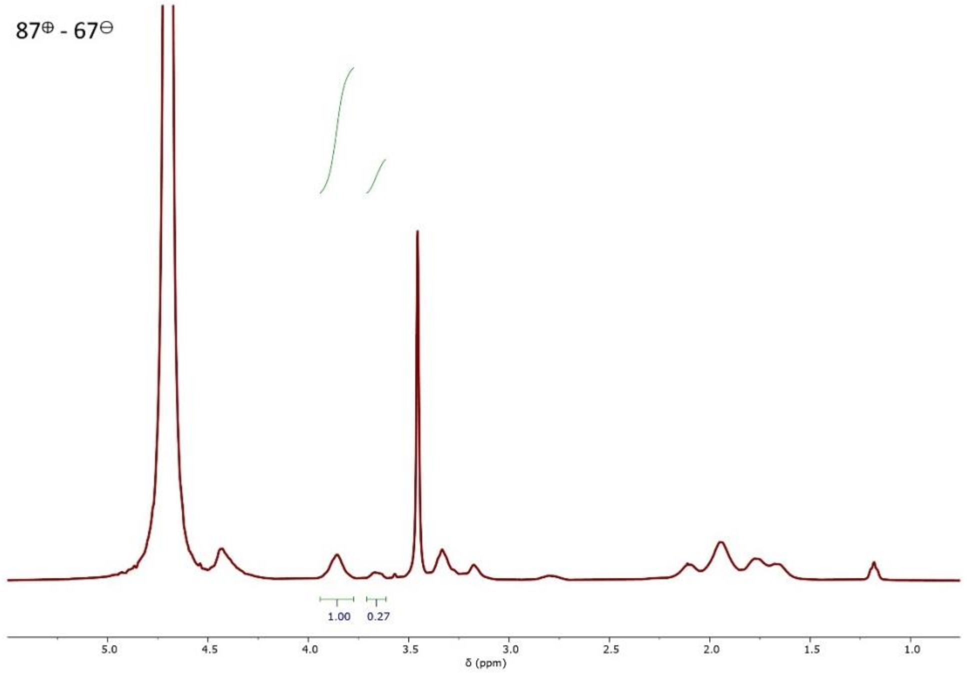

Figure S 19: ^{1}H-NMR spectrum (400 MHz, 2.5 M KBr D_2O) of 87⊕-67⊖.

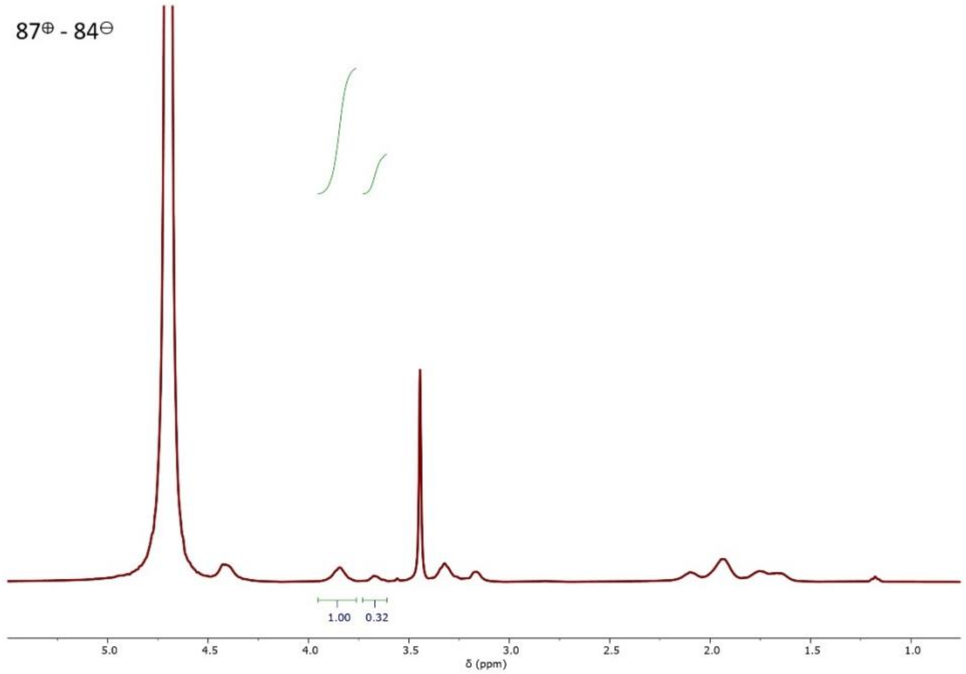

Figure S 20 :^{1}H-NMR spectrum (400 MHz, 2.5 M KBr D_2O) of 87⊕-84⊖.

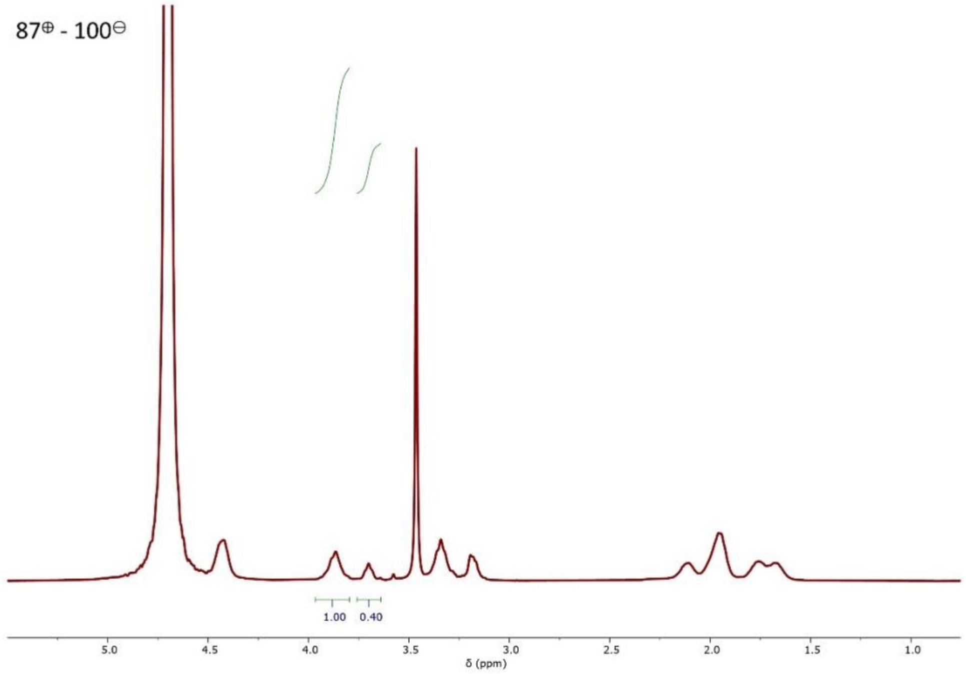

Figure S 21: ^{1}H-NMR spectrum (400 MHz, 2.5 M KBr D_2O) of 87⊕-100⊖.

4. ^{13}C-NMR data

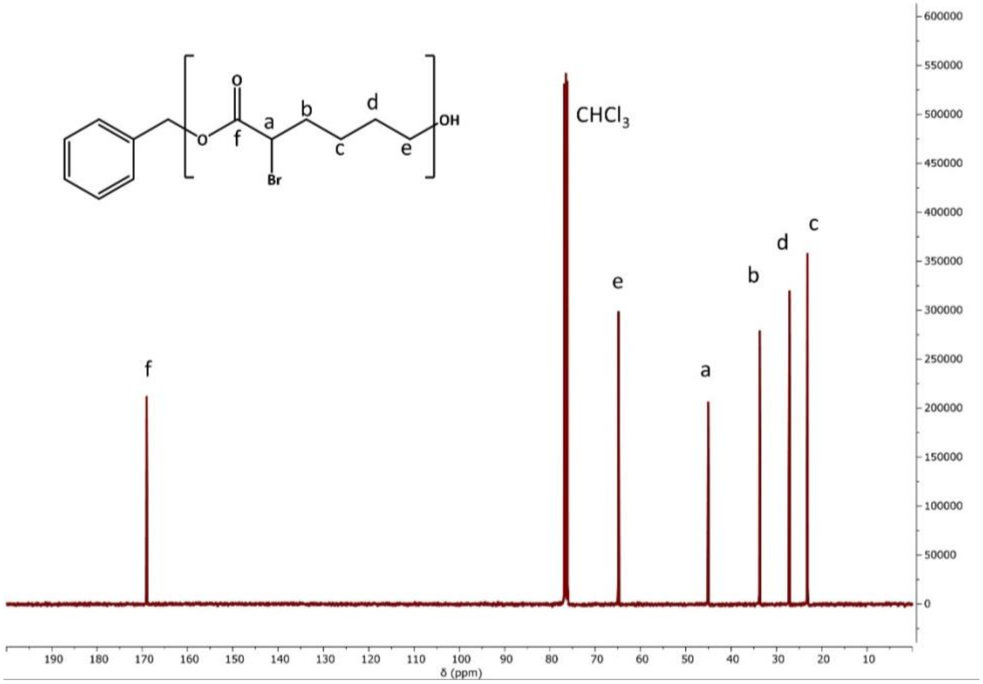

Figure S 22: ^{13}C-NMR spectrum of P(αBrCL) (101 MHz, $CDCl_3$): δ 23.15 (–CH_2–), 27.13 (–CH_2–), 33.69 (–CH_2–), 45.04 (CH–Br), 64.85 (–COOCH_2–), 169.04(–COO–).

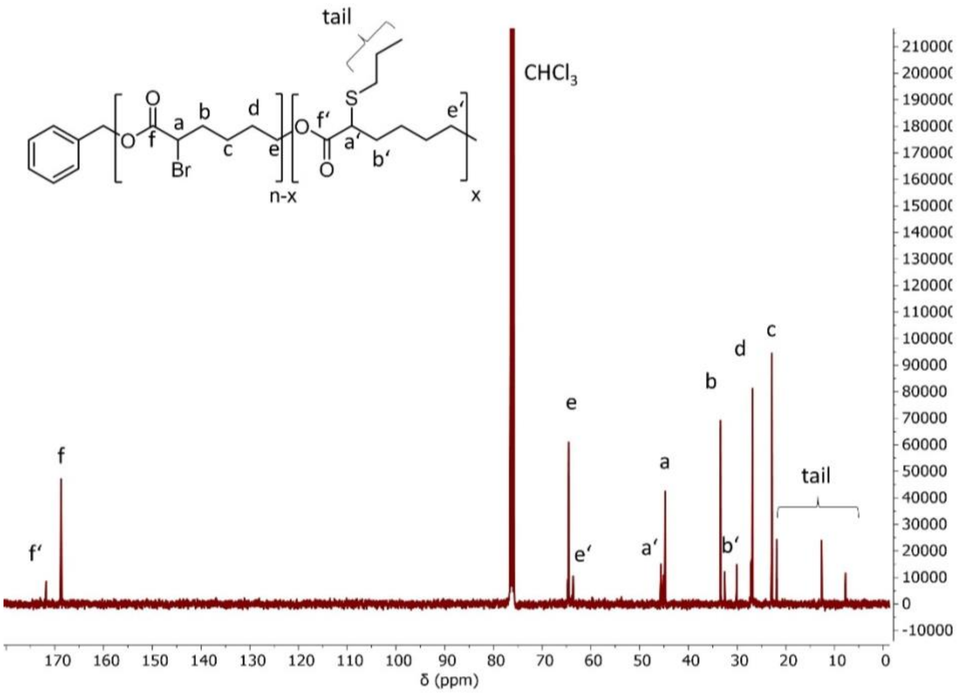

Figure S 23: ^{13}C-NMR spectrum of P(αBrCL)-(84%) (101 MHz, $CDCl_3$): δ 7.75, 12.65, 21.88 (alkyl tail), 22.84, 22.95, 26.83, 27.06, 27.20, 30.10 ("grease"), 32.53, 33.39, 33.42 (polymer backbone), 44.76 (–*C*H–S), 45.60(*C*H–Br), 63.65, , 64.75 (–*C*H_2O–), 168.74, 171.83 (–*C*OO–).

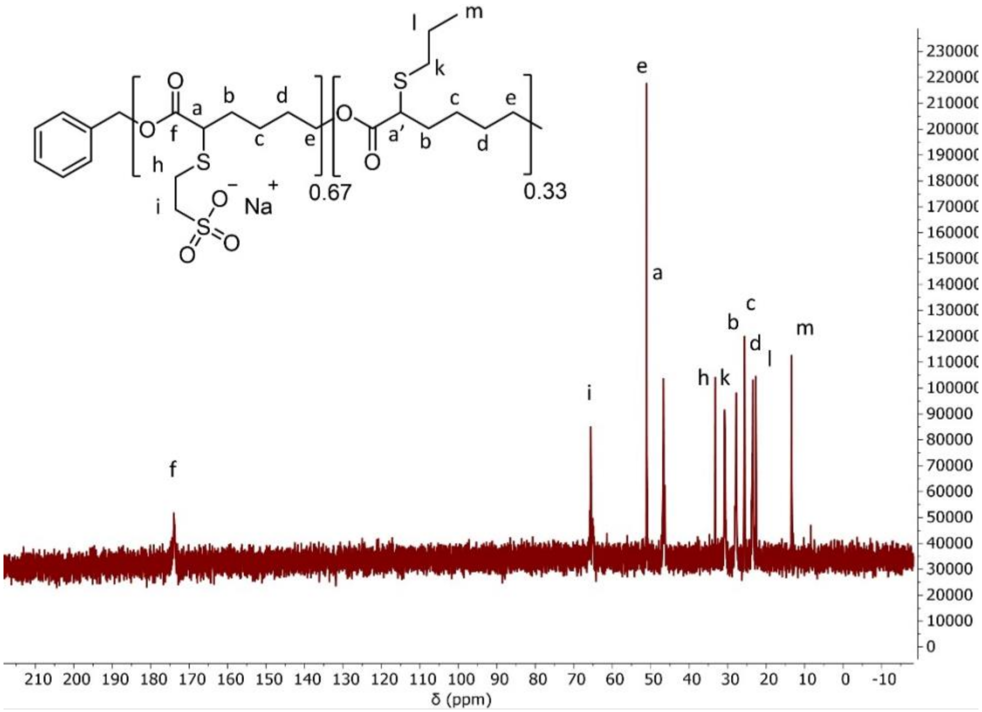

Figure S 24:^{13}C-NMR spectrum of P67⊖ (101 MHz, 2.5 M KBr D_2O) δ 13.40 (–CH_2*C*H_3–), 22.69 (–*C*H_2CH_3–), 23.49, 25.61, 27.81 (–*C*H_2*C*H_2*C*H_2–), 30.66 (–*C*H_2S(CH_2CH_3)–), 33.26 (–*C*H_2S–), 46.37 (–*C*HS–), 51.09 (–*C*H_2O–), 65.62 (–*C*H_2SO_3–), 174.00 (–*C*OO–).

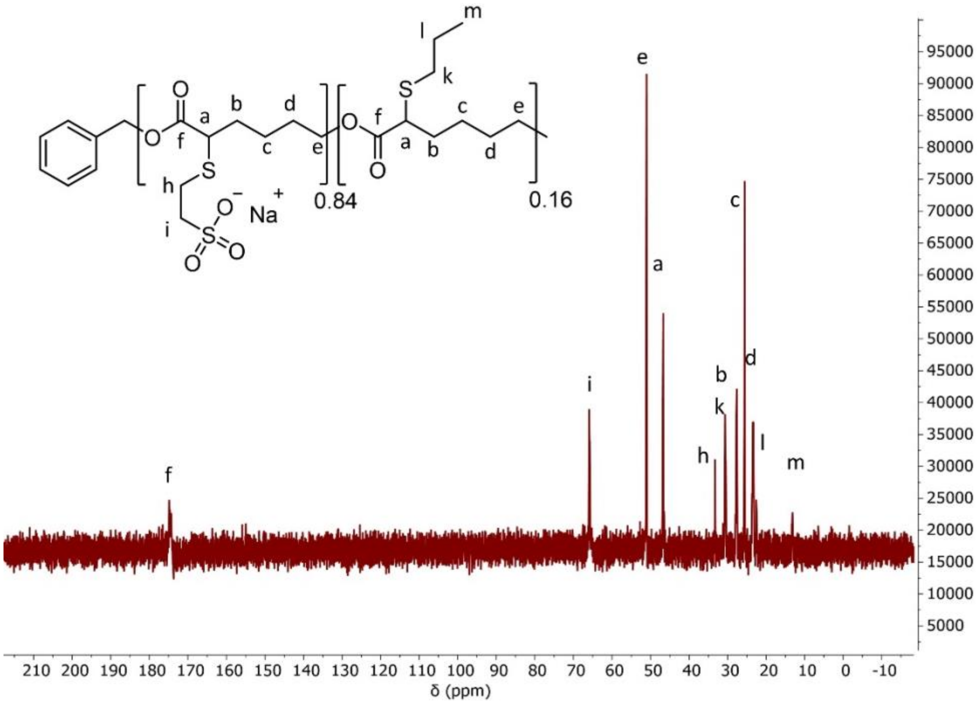

Figure S 25: ^{13}C-NMR spectrum of P84⊖ (101 MHz, 2.5 M KBr D_2O) *δ* 13.17 (–CH_2*C*H_3–), 22.56 (–*C*H_2CH_3–), 23.31, 25.58, 27.66 (–*C*H_2*C*H_2*C*H_2–), 30.62(–*C*$H_2S(CH_2CH_3)$–), 33.27 (–*C*H_2S–), 46.72 (–*C*HS–), 51.04 (–*C*H_2O–), 65.83 (–*C*H_2SO_3–), 174.86 (–*C*OO–).

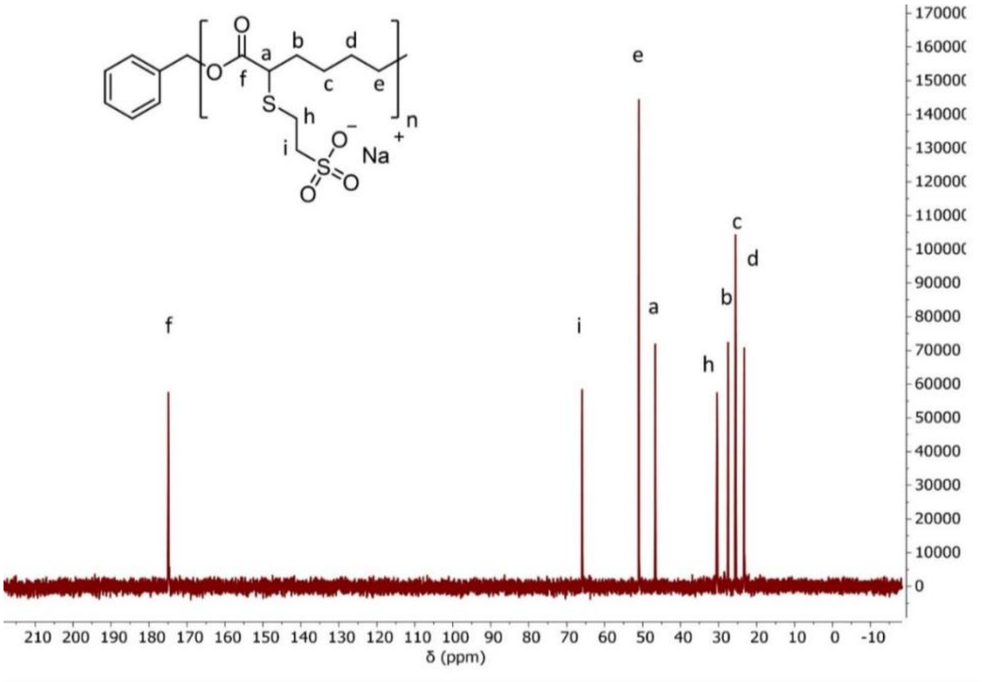

Figure S 26: ^{13}C-NMR spectrum of P100⊖ (101 MHz, 2.5 M KBr D_2O) *δ* 23.24 (–*C*H_2–), 25.53 (–*C*H_2–), 27.52 (–*C*H_2–), 30.41(–*C*H_2S–), 46.69(–*CH(*S)–), 50.99 (–*C*H_2O–), 65.94 (–*C*H_2SO_3–), 174.90 (–*COO*–).

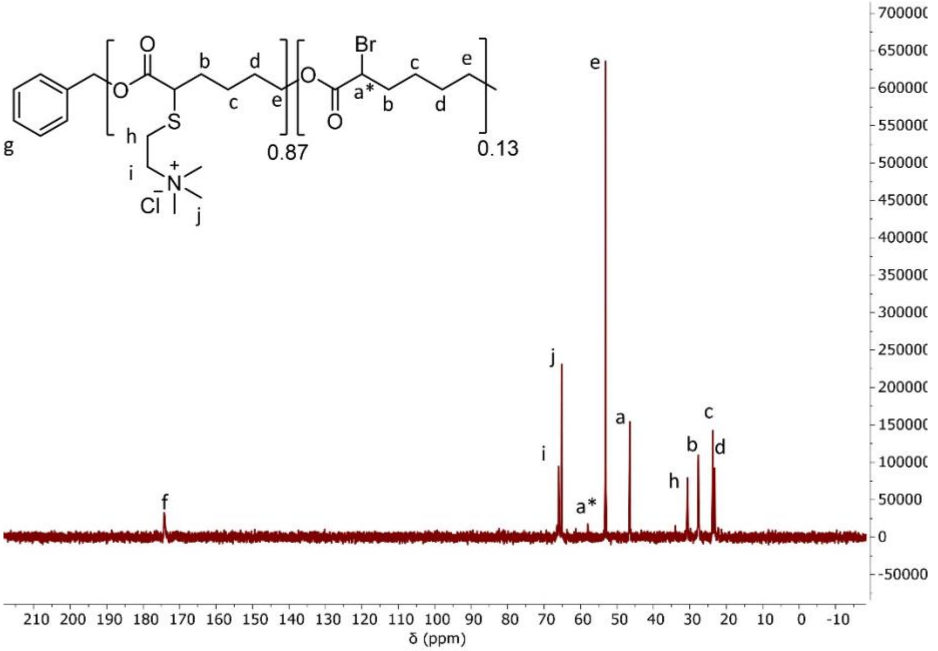

Figure S 27: ^{13}C-NMR spectrum of P87⊕ (101 MHz, 2.5 M KBr D_2O) *δ* 23.25 (–*CH_2*–), 23.70 (–*CH_2*–), 27.66 (–*CH_2*–), 30.61 (–*CH_2*S–), 46.47 (–*CH(S)*–), 53.10 (–*CH_2*O–), 65.16(–*CH_3*N–), , 65.95(–*CH_2*N–), 174.21 (–*COO*–).

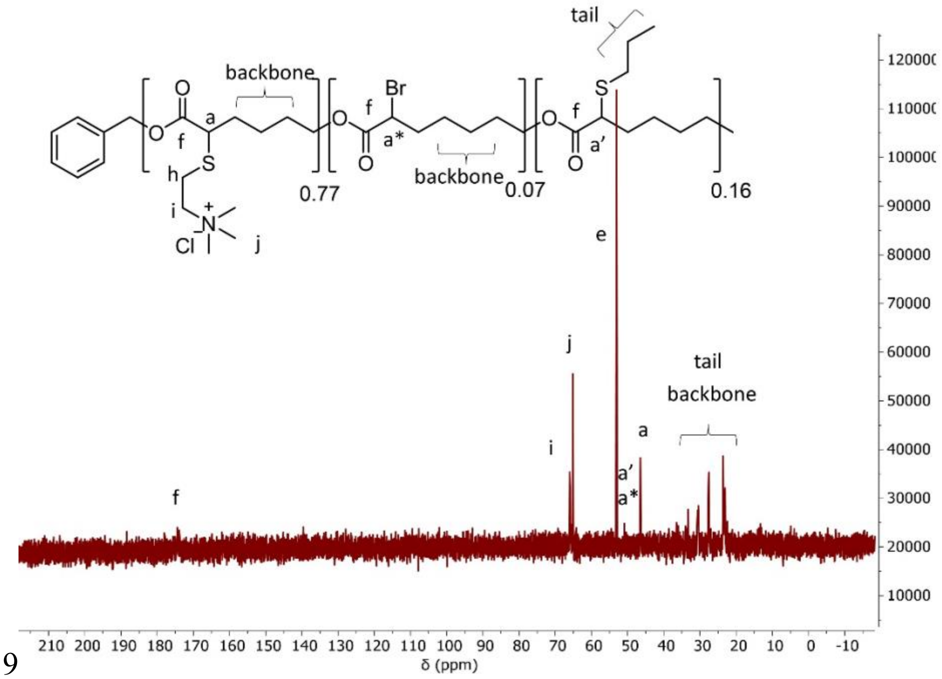

9

Figure S 28: ^{13}C-NMR spectrum of P77⊕ (101 MHz, 2.5 M KBr D_2O) *δ* 13.41, 13.45, 22.70, 23.41, 23.77, 27.89, 30.89, 33.26 (backbone & tail), 46.38 (–*C*H–S), 53.13 (–*C*H_2O–), 65.14 (–*C*H_3N–), 65.66 (–CH_2*C*H_2S–), 173.54 (–COO–).

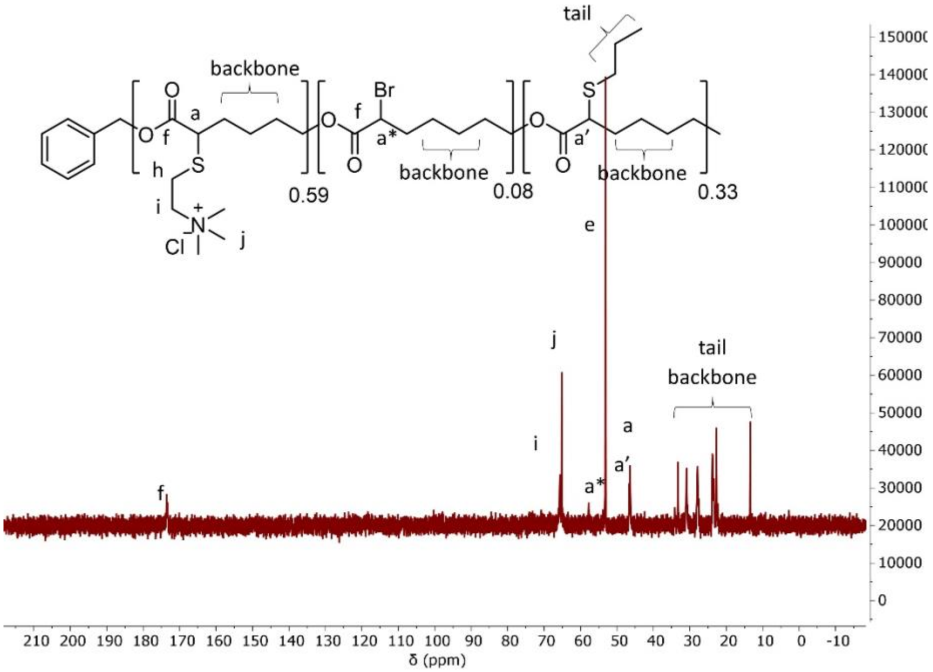

Figure S 29: ^{13}C-NMR spectrum of P59⊕ (101 MHz, 2.5 M KBr D_2O) *δ* 23.05, 23.60, 27.58 (backbone & tail), 46.46 (–*C*H–S), 53.05 (–*C*H_2O–), 65.15 (–*C*H_3N–), 65.98 (–CH_2*C*H_2S–), 175.01 (–COO–).

5. DOSY NMR data

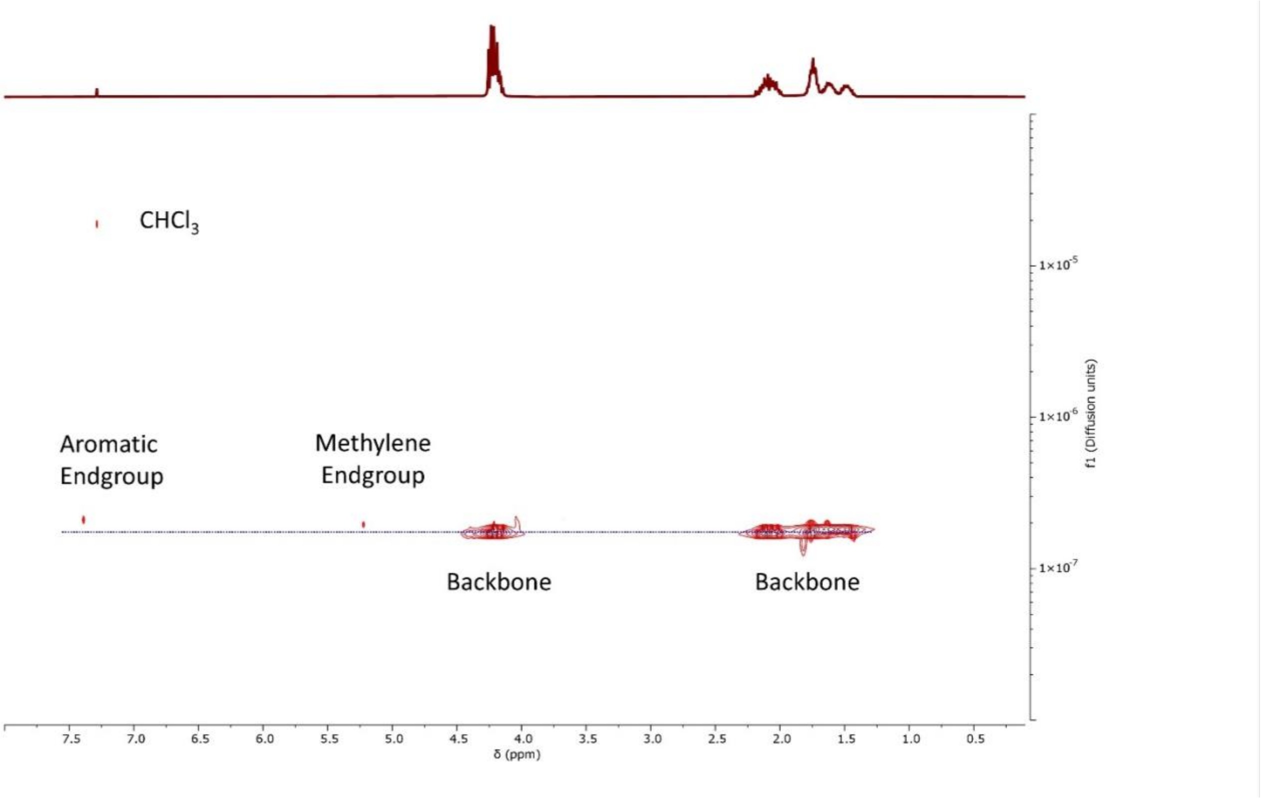

Figure S 30: DOSY NMR spectrum of P(αBrCL) at 400 mHz in $CDCl_3$. The proton signals corresponding to the polymer backbone have similar diffusion units (i.e. they are aligned horizontally). The end groups are highlighted at slightly higher diffusion units (due to their larger mobility compared to the internal chain segments). The solvent signal ($CHCl_3$) can be found at higher diffusion values (1×10^{-5}).

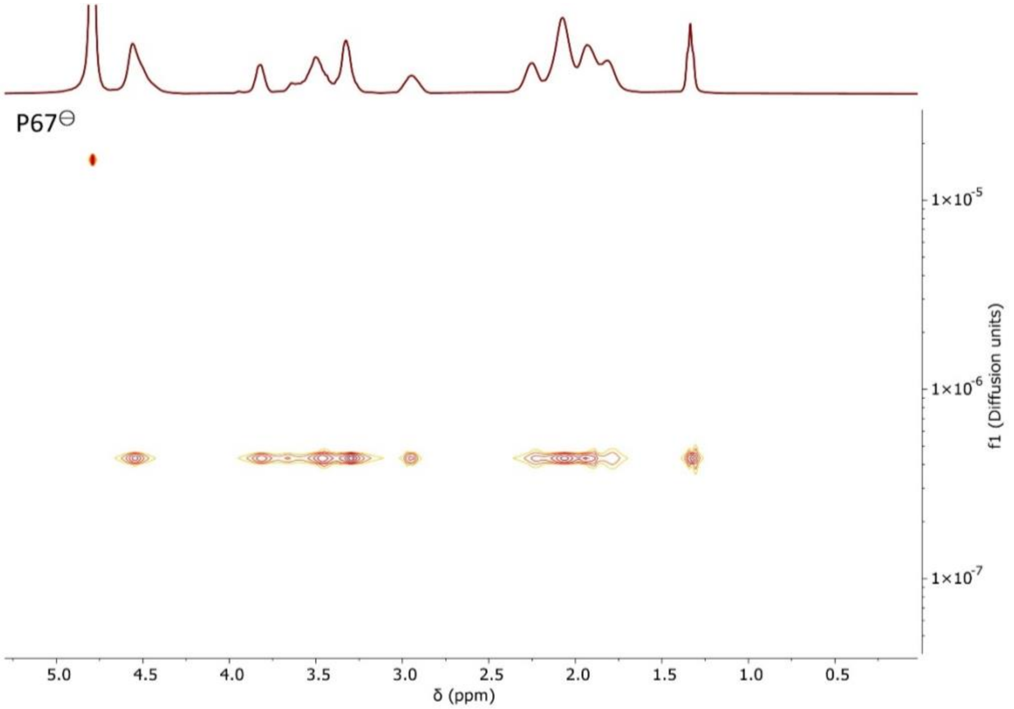

Figure S 31: DOSY NMR spectrum of P67⊖ at 400 mHz in D_2O. The proton signals corresponding to the polymer backbone and the attached groups are aligned horizontally. The solvent signal (H_2O) can be found at higher diffusion values (1×10^{-5}).

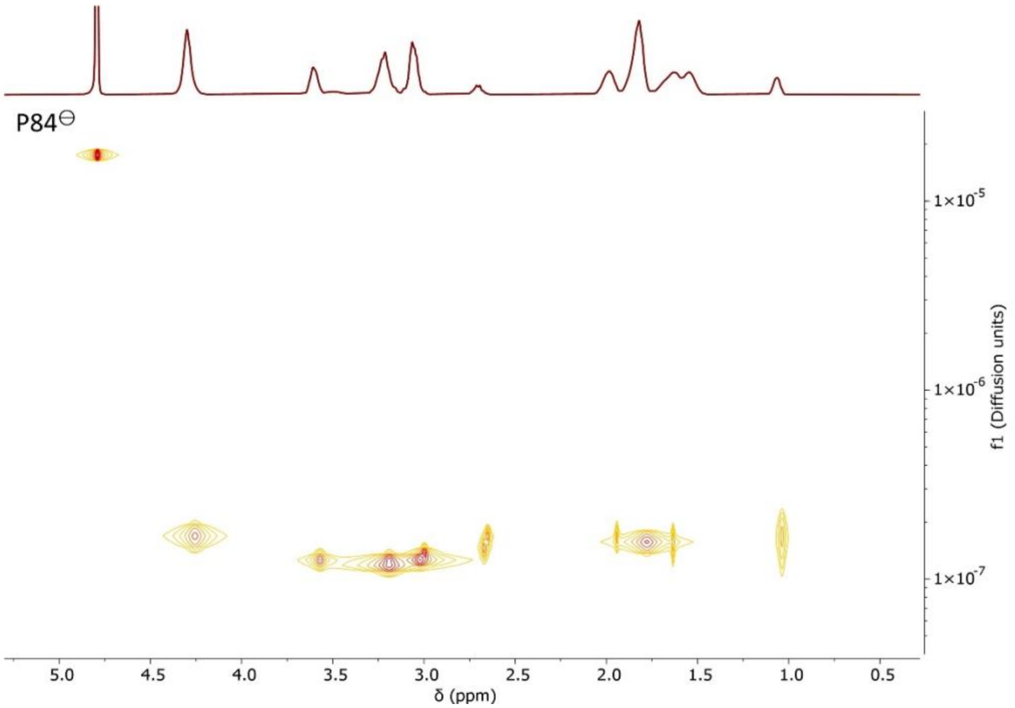

Figure S 32: DOSY NMR spectrum of P84⊖ at 400 mHz in D_2O. The proton signals corresponding to the polymer backbone and the attached groups are aligned horizontally. The solvent signal (H_2O) can be found at higher diffusion values (1×10^{-5}).

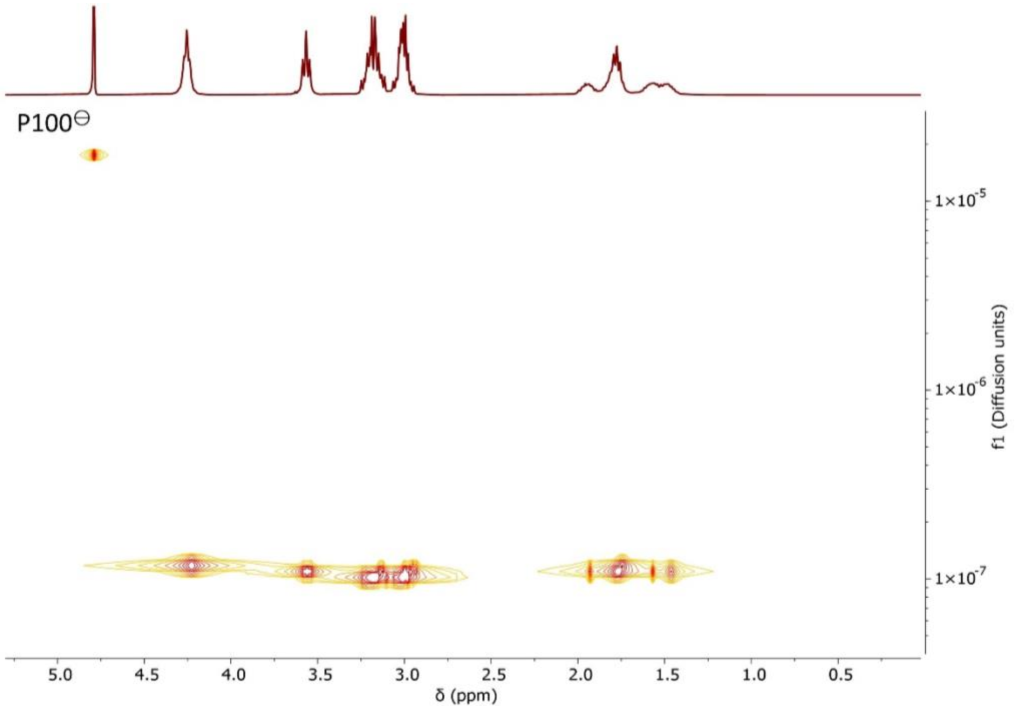

Figure S 33: DOSY NMR spectrum of P100 at 400 mHz in D_2O. The proton signals corresponding to the polymer backbone and the attached groups are aligned horizontally. The solvent signal (H_2O) can be found at higher diffusion values (1×10^{-5}).

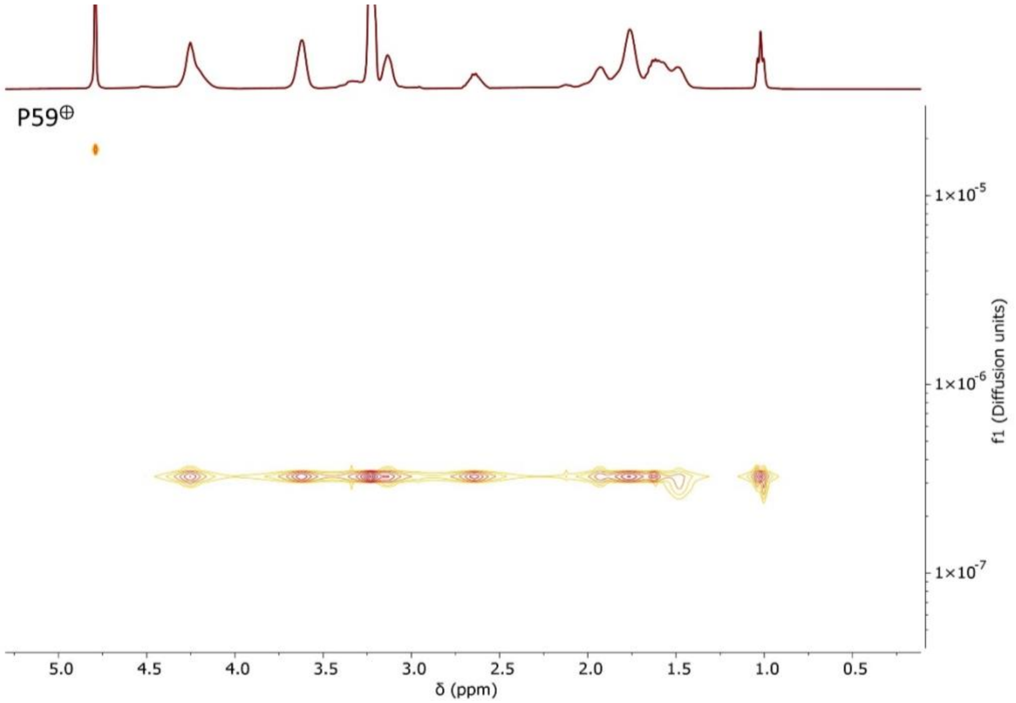

Figure S 34: DOSY NMR spectrum of P59⊕ at 400 mHz in D_2O. The proton signals corresponding to the polymer backbone and the attached groups are aligned horizontally. The solvent signal (H_2O) can be found at higher diffusion values (1×10^{-5}).

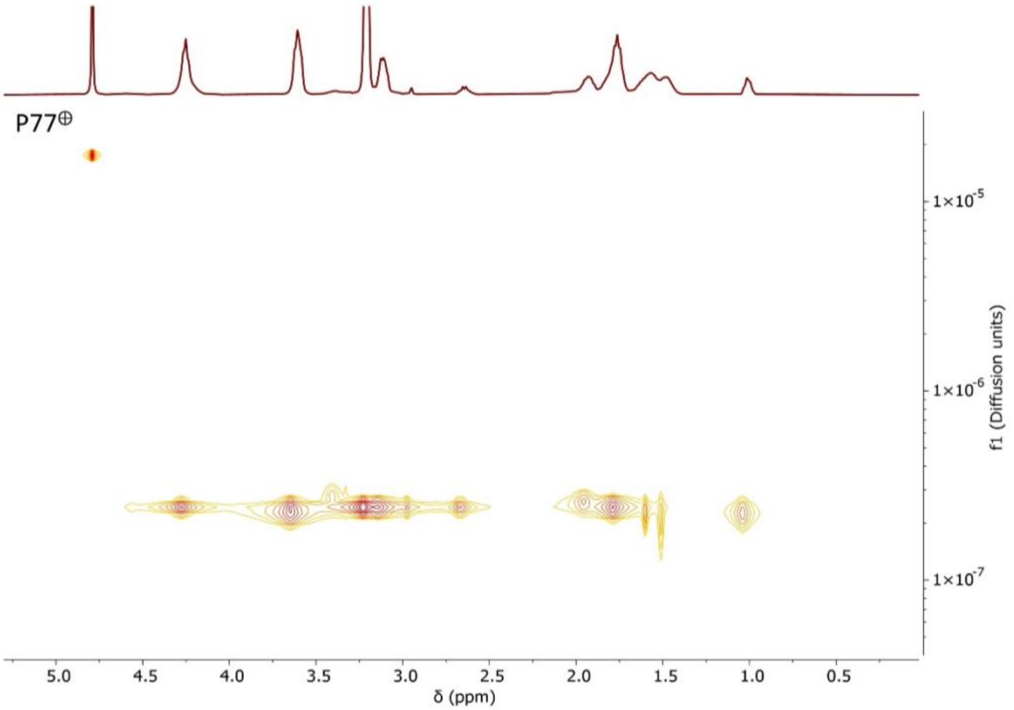

Figure S 35: DOSY NMR spectrum of P77⊕ at 400 mHz in D_2O. The proton signals corresponding to the polymer backbone and the attached groups are aligned horizontally. The solvent signal (H_2O) can be found at higher diffusion values (1×10^{-5}).

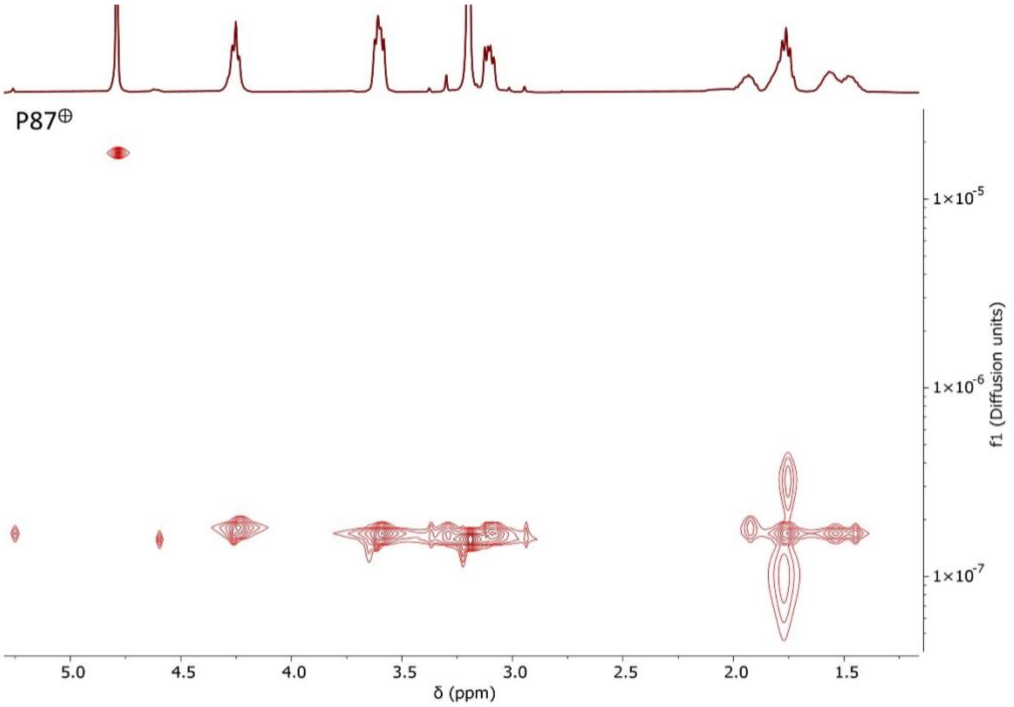

Figure S 36: DOSY NMR spectrum of P87⊕ at 400 mHz in D_2O. The proton signals corresponding to the polymer backbone and the attached groups are aligned horizontally. The solvent signal (H_2O) can be found at higher diffusion values (1×10^{-5}).

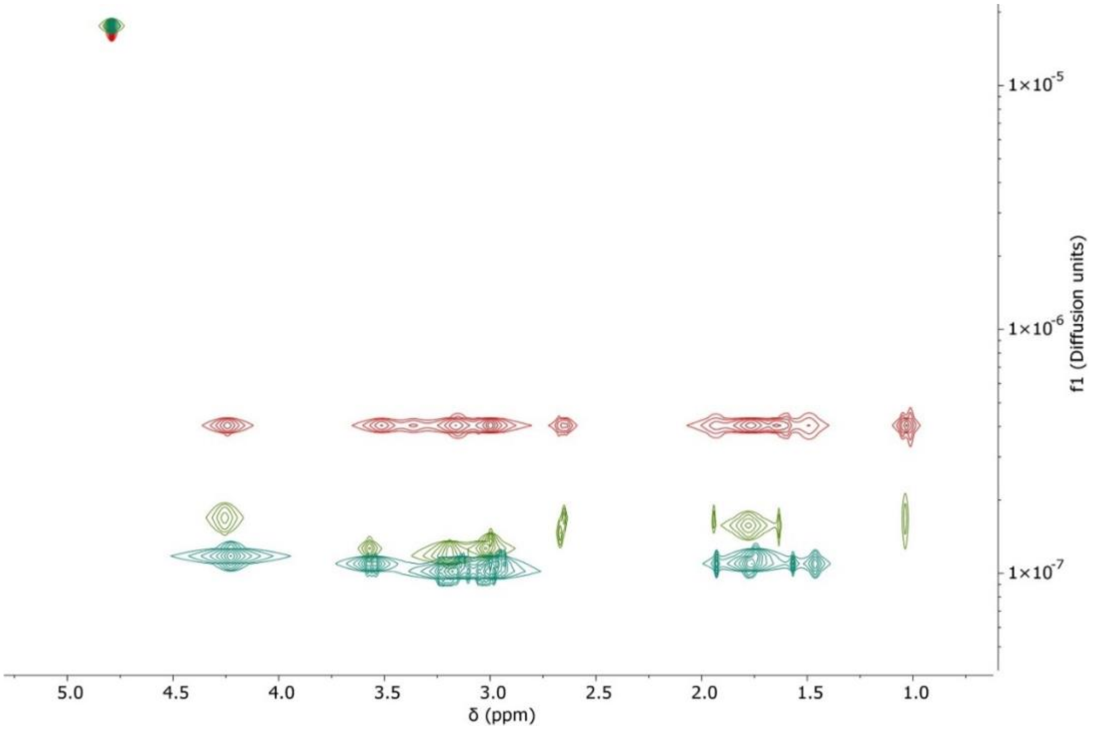

Figure S 37: Stacked DOSY NMR spectrum of the polyanions P67⊖ (top, red), P84⊖ (middle, light green), and P100⊖ (bottom, dark green) at 400 mHz in D_2O. The polyelectrolytes with lower charge densities are clearly distinguishable from 100⊖ due to the signals at 1.0 ppm. The solvent signal (H_2O) can be found at higher diffusion values (1×10^{-5}).

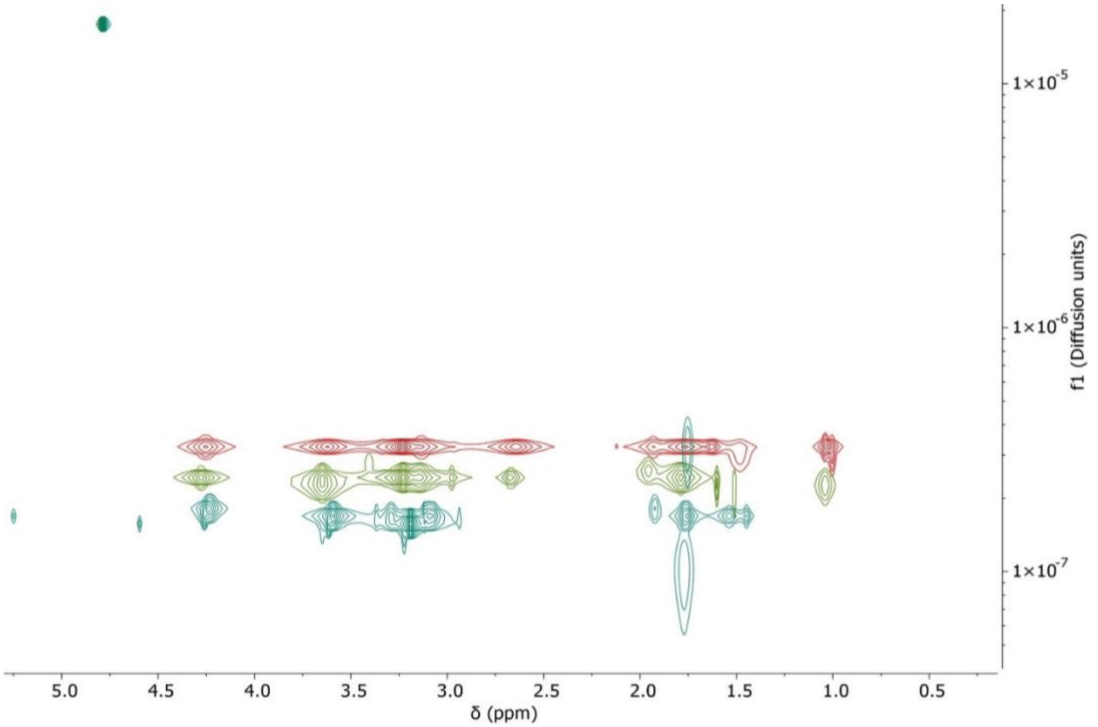

Figure S 38: Stacked DOSY NMR spectrum of the polyanions P59⊕ (top, red), P77⊕ (middle, light green), and P87⊕ (bottom, dark green) at 400mHz in D_2O. The polyelectrolytes with lower charge densities are clearly distinguishable from 87⊕ due to the signals at 1.0 ppm. The solvent signal (H_2O) can be found at higher diffusion values (1×10^{-5}).

6. Size Exclusion Chromatography (SEC) data

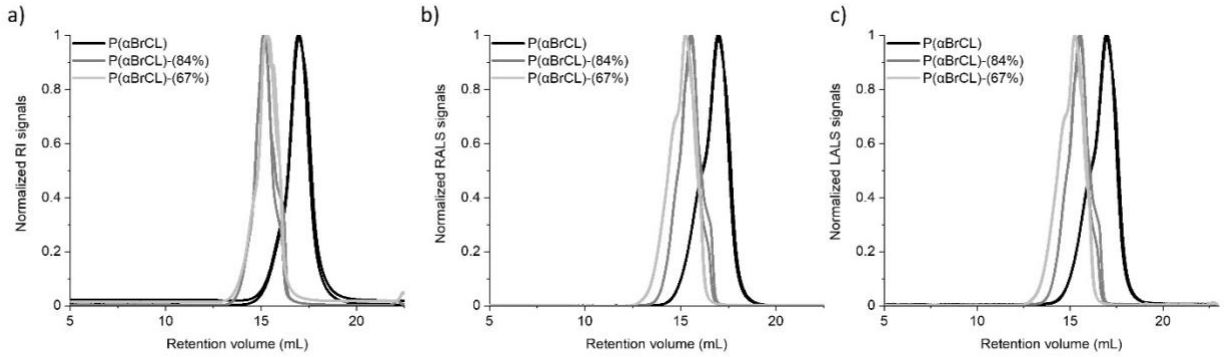

Figure S 39: Detector signals of the a) refractive index (RI), b) the right-angle light scattering (RALS), and c) the low-angle light scattering (LALS) of the SEC measurements of P(αBrCL) with different degrees of substitution of the bromine by propane thiol, recorded in HFIP with 0.02 M KTFA.

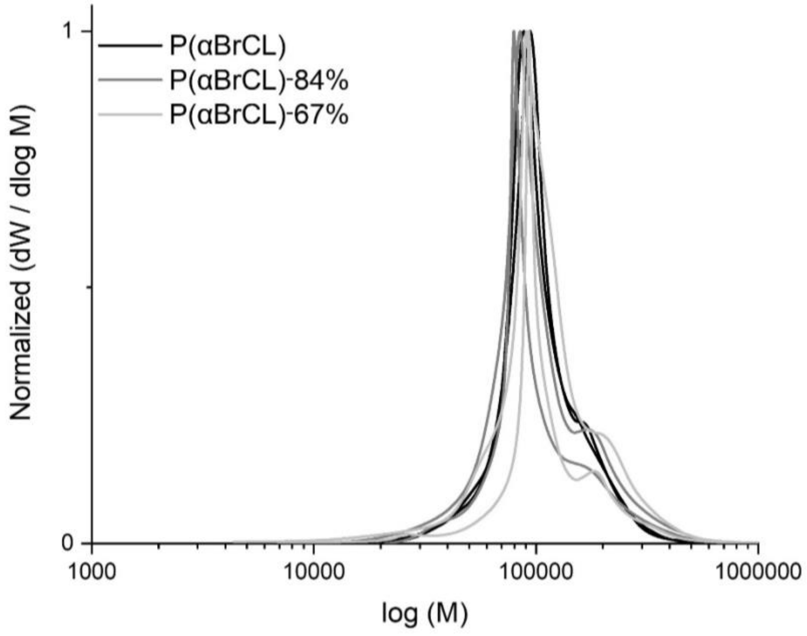

Figure S 40: Molecular weight distribution of P(αBrCL) with different degrees of substitution of the bromine by propane thiol, calculated from the SEC measurements (see Figure S39).

7. TGA data

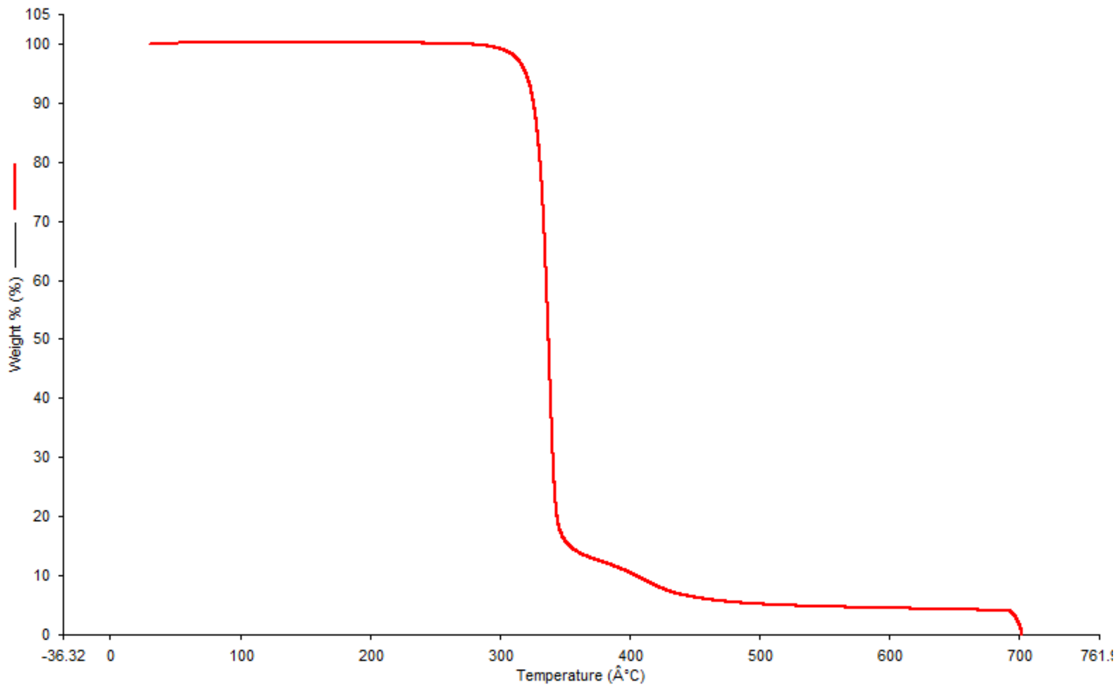

Figure S 41: TGA plot of P(αBrCL), dried in the vacuum oven, under N_2 atmosphere. After reaching 700 °C, an isothermal step was applied for 30 min under air atmosphere.

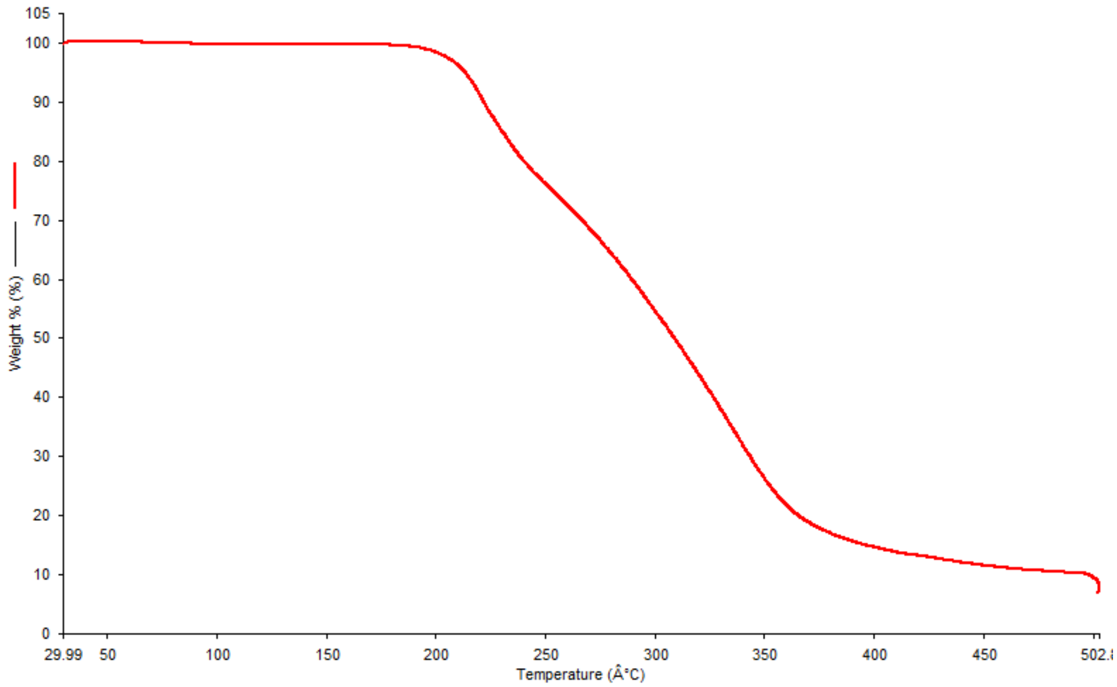

Figure S 42: TGA plot of P59⊕, dried in the vacuum oven, under N_2 atmosphere. After reaching 500 °C, an isothermal step was applied for 30 min under air atmosphere.

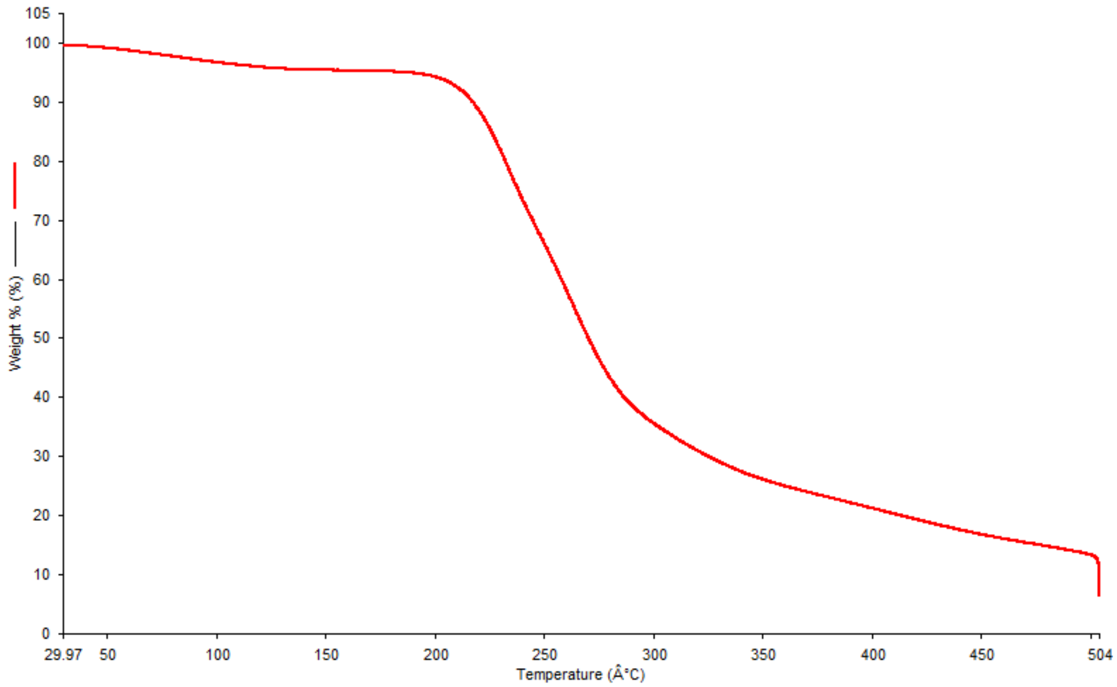

Figure S 43: TGA plot of P77⊕, dried in the vacuum oven, under N_2 atmosphere. After reaching 500 °C, an isothermal step was applied for 30 min under air atmosphere.

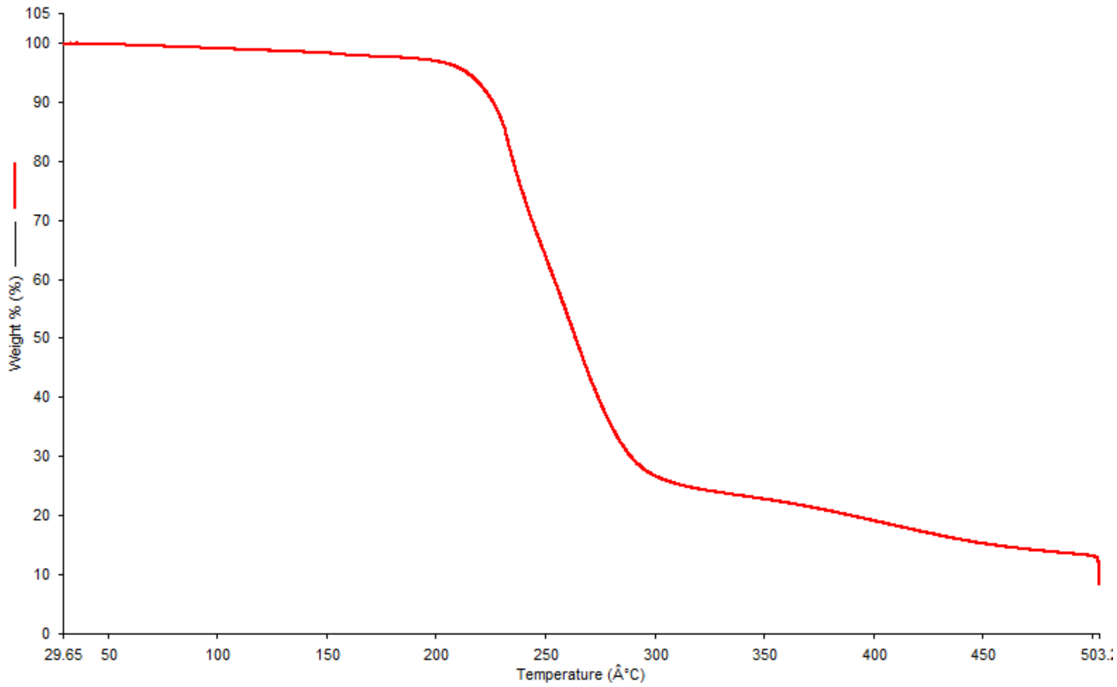

Figure S 44: TGA plot of P87⊕, dried in the vacuum oven, under N_2 atmosphere. After reaching 500 °C, an isothermal step was applied for 30 min under air atmosphere.

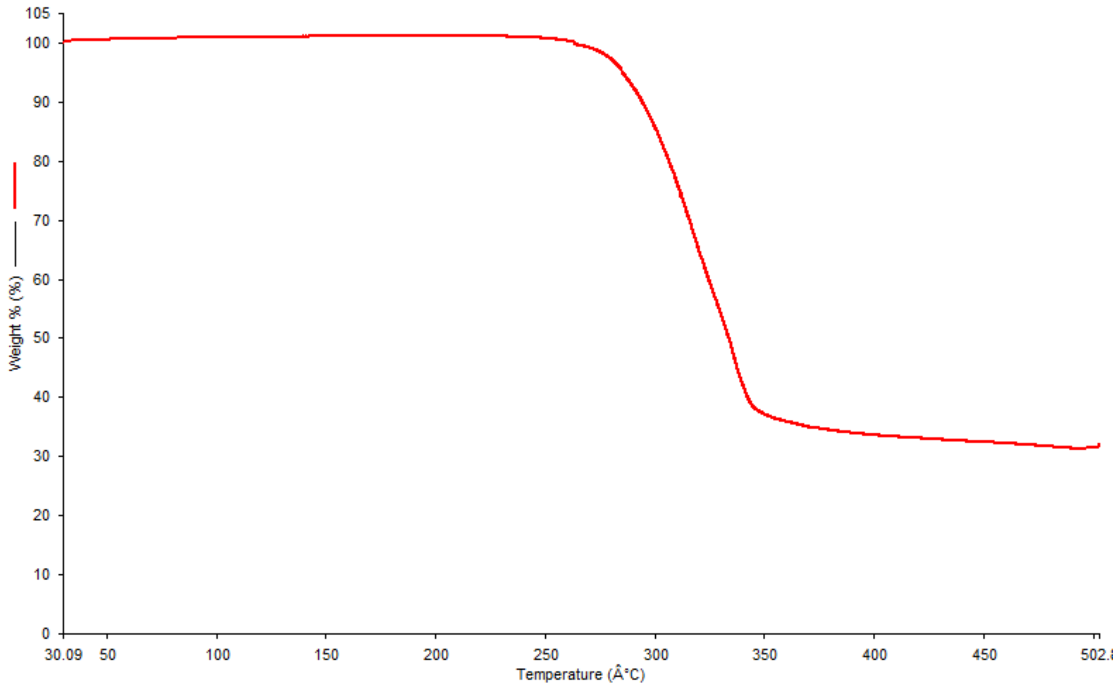

Figure S 45: TGA plot of P67⊖, dried in the vacuum oven, under N_2 atmosphere.

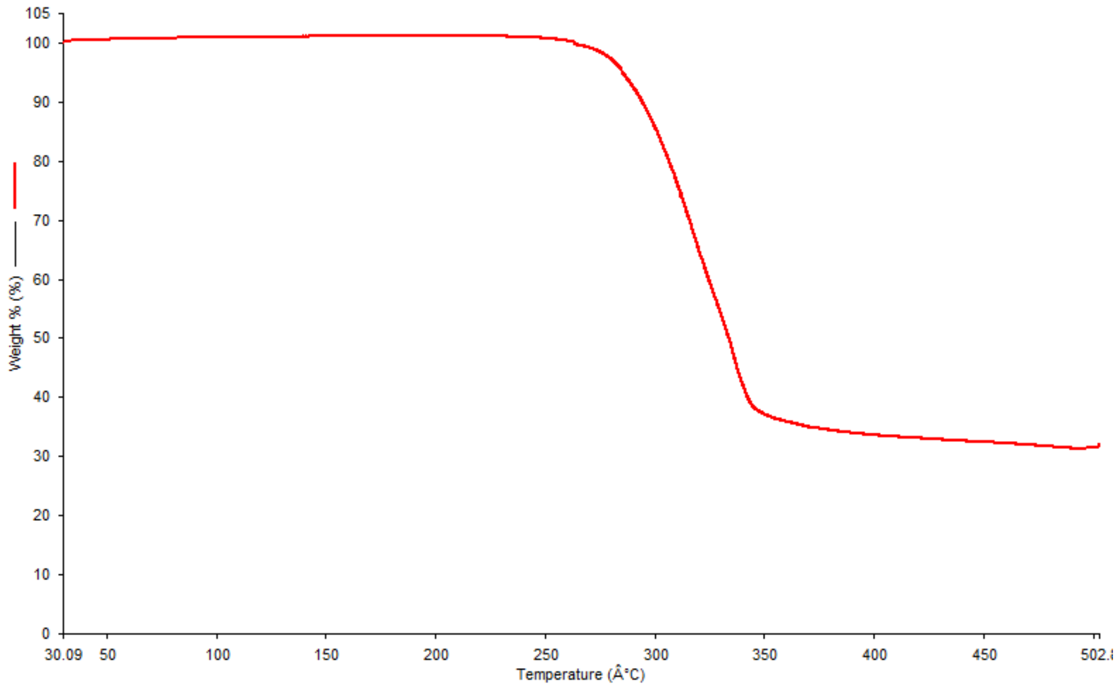

Figure S 46: TGA plot of P84⊖, dried in the vacuum oven, under N_2 atmosphere.

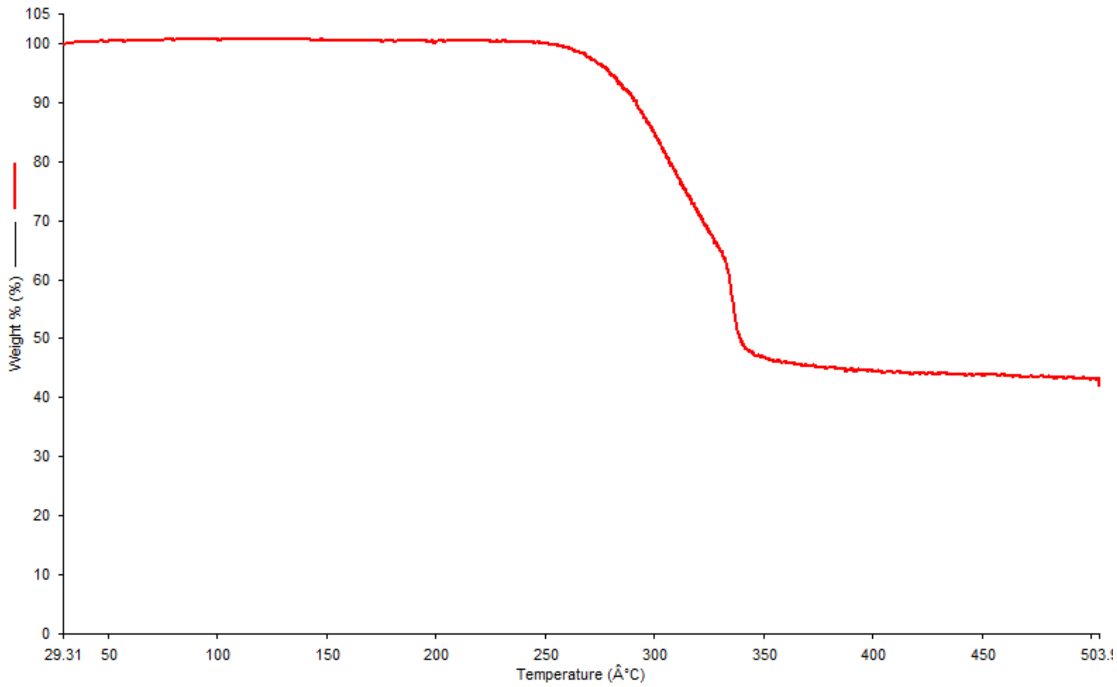

Figure S 47: TGA plot of P100⊖, dried in the vacuum oven, under N_2 atmosphere.

8. DSC data

The T_g values were derived by determining the inflection point of the 2nd heating curve within a defined analysis limit.

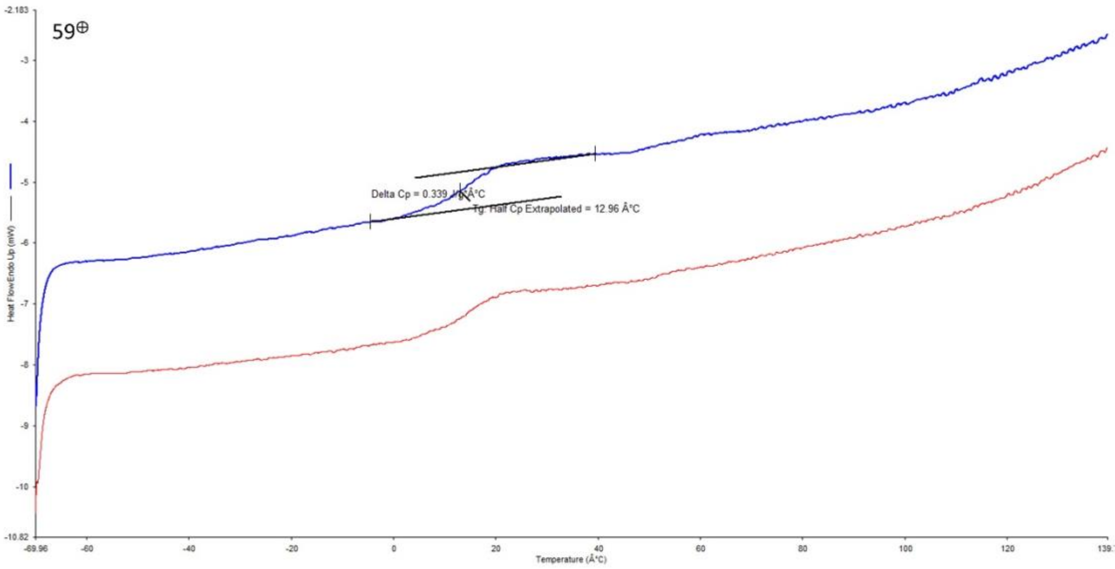

Figure S 48: DSC run of P57⊕ dried in the vacuum oven. The 1st heating is displayed in red and the 2nd heating in blue.

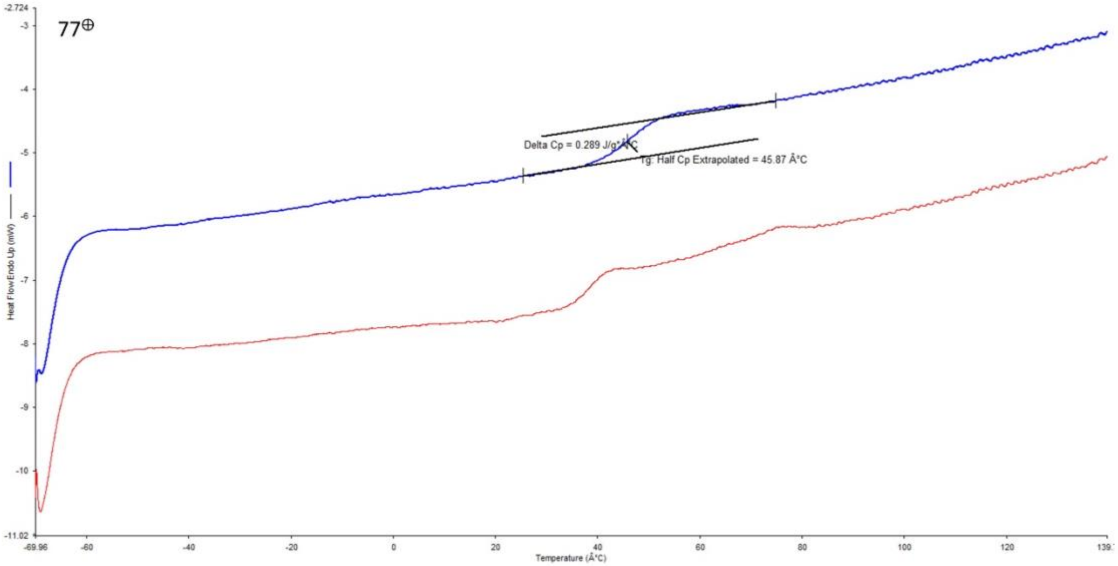

Figure S 49: DSC run of P77⊕ dried in the vacuum oven. The 1st heating is displayed in red and the 2nd heating in blue.

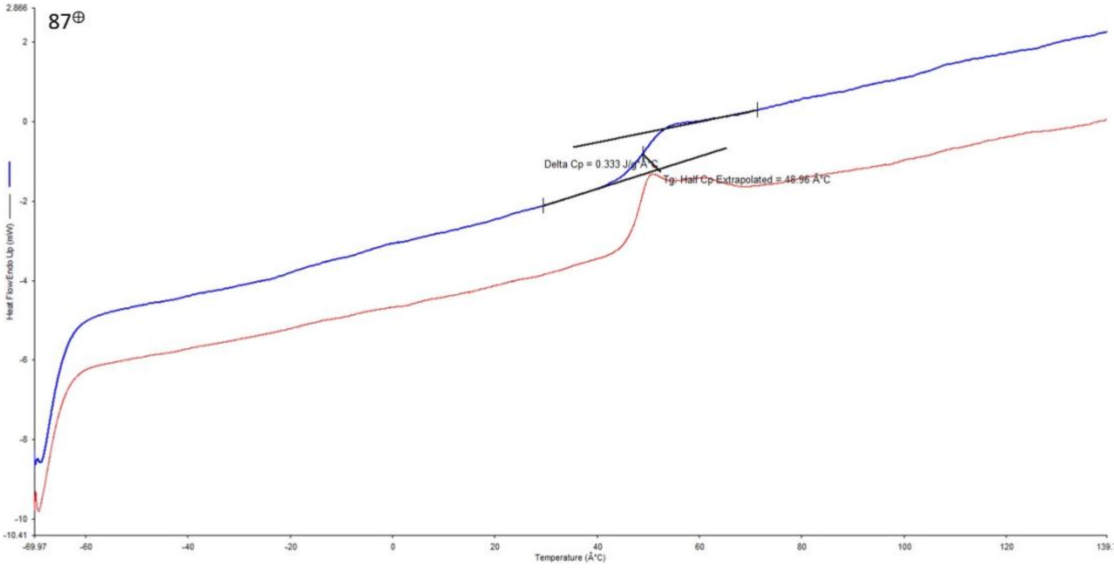

Figure S 50: DSC run of P87 dried in the vacuum oven. The 1st heating is displayed in red and the 2nd heating in blue.

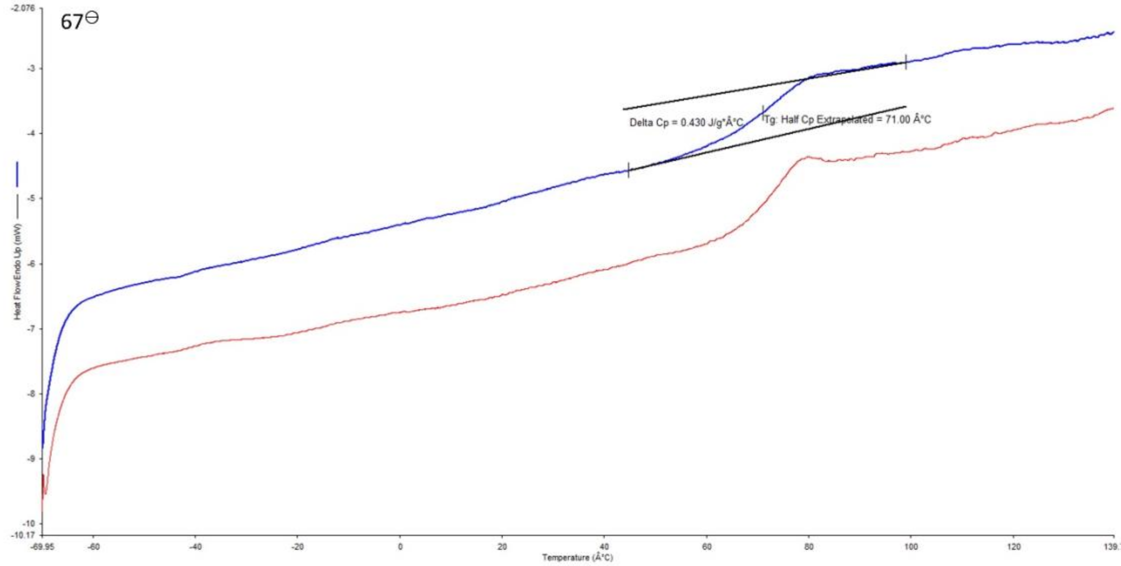

Figure S 51: DSC run of P67⊖ dried in the vacuum oven. The 1st heating is displayed in red and the 2nd heating in blue.

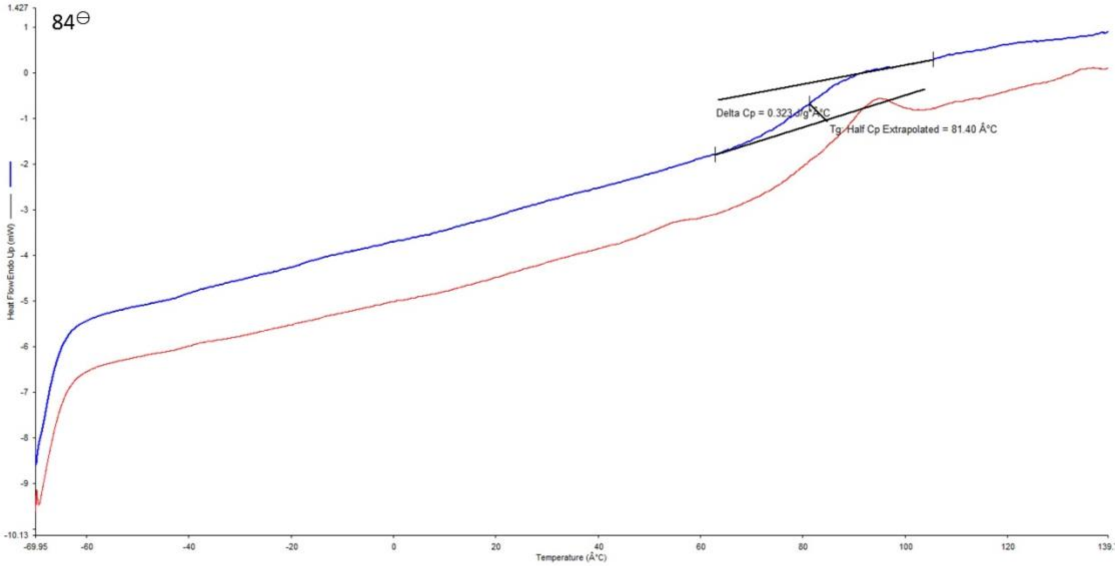

Figure S 52: DSC run of P84⊖ dried in the vacuum oven. The 1st heating is displayed in red and the 2nd heating in blue.

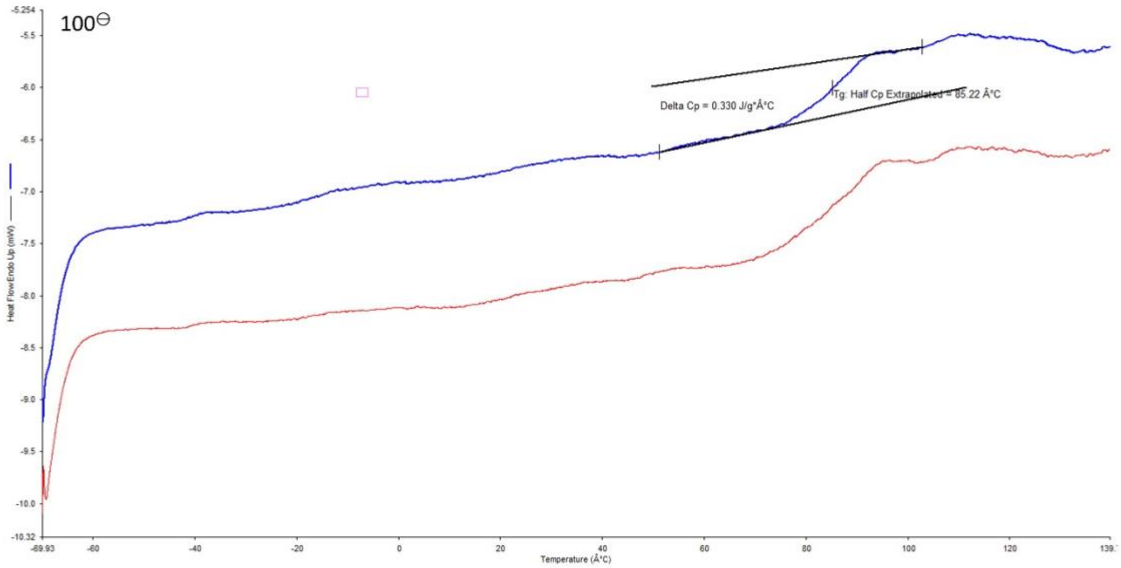

Figure S 53: DSC run of P100⊖ dried in the vacuum oven. The 1st heating is displayed in red and the 2nd heating in blue.

9. Theoretical considerations

The calculations of average charge density and relative charge mismatch values of each complex combination displayed in Figure 4 are shown in Table S 6.

Complex	P⊕	P⊖	P_{av}	dP	dP/P_{av}	CSC_{min}	CSC_{max}	CSC_{av}
59⊕-67⊖	59	67	63	-8	-0.127	1.75	2.00	1.875
77⊕-67⊖	77	67	72	10	0.139	1.00	1.25	1.125
87⊕-67⊖	87	67	77	20	0.260	1.00	1.25	1.125
59⊕-84⊖	59	84	71.5	-25	-0.350	0.650	0.75	0.700
77⊕-84⊖	77	84	80.5	-7	-0.087	1.25	1.50	1.375
87⊕-84⊖	87	84	85.5	3	0.035	1.25	1.50	1.375
59⊕-100⊖	59	100	79.5	-41	-0.516	0.50	0.65	0.575
77⊕-100⊖	77	100	88.5	-23	-0.260	1.25	1.50	1.375
87⊕-100⊖	87	100	93.5	-13	-0.139	1.50	1.75	1.625

Table S 6: P⊕ and P⊖ refer to the charge density of polycation and polyanion respectively. The average charge density is given by P_{av}. The charge mismatch is dP, and the relative charge mismatch dP/$P_{av.}$ The CSC_{av} refers to the average CSC, which is obtained from the range of CSC_{min} and CSC_{max}.

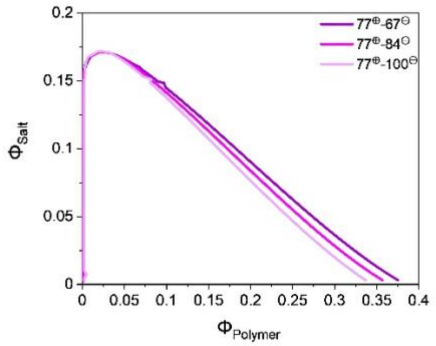

Figure S 54: Mean-field, theoretical phase diagrams of the polyester polyelectrolyte complexes of P77⊕ displaying the derived binodal lines. The χ-parameters for non-electrostatic interactions were included for this approach, similar to Figure 5.

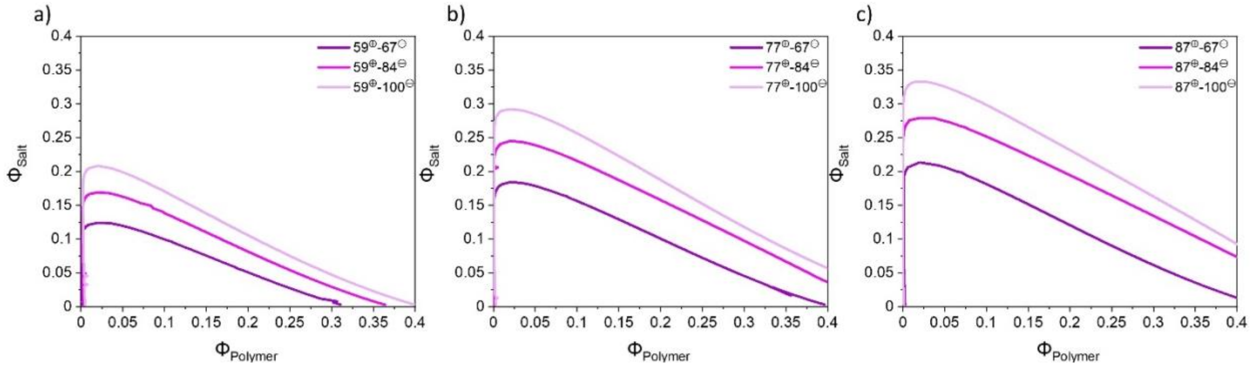

Figure S 55: Mean-field, theoretical phase diagrams of the polyester polyelectrolyte complexes displaying the derived binodal lines. The mixtures of P59⊕ are shown in panel a), of P77⊕ in b), and of P87⊕ in c). The χ-parameters for non-electrostatic interactions were excluded for this approach.

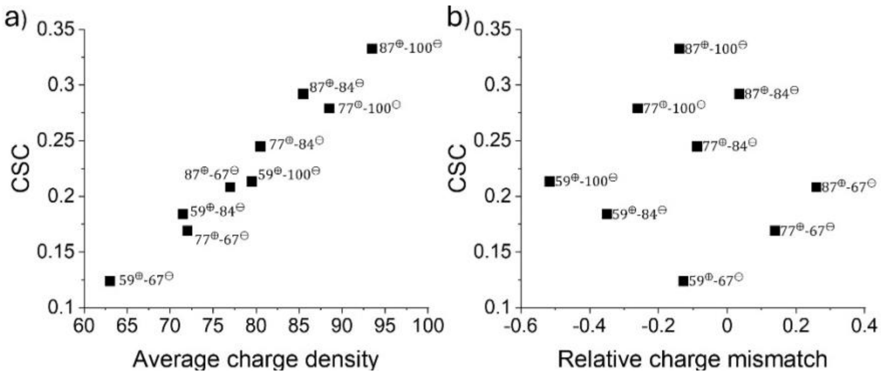

Figure S 56: Matrix of the derived critical salt concentration (CSC) from the binodal lines against the average charge density in a) and against the relative charge mismatch in b). The χ parameters for the non-electrostatic interactions are excluded from the calculations.

10.Photographs

Polymer synthesis

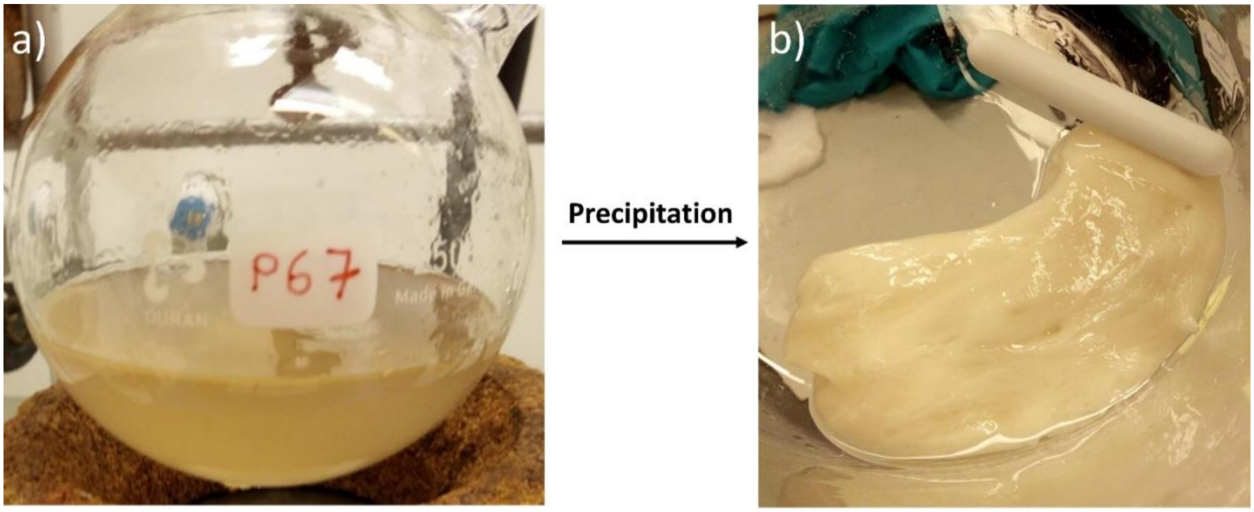

Figure S 57: Photographs of the a) crude polymer mixture of P(αBrCL) after termination, and b) the precipitated polymer.

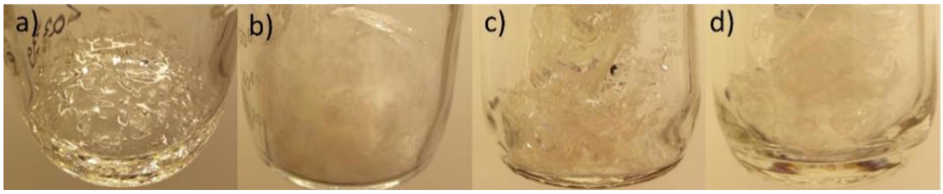

Figure S 58: Photographs of dried polyelectrolytes, a) P87$^{\oplus}$, b) P100$^{\ominus}$, c) P59$^{\oplus}$ d) P67$^{\ominus}$.

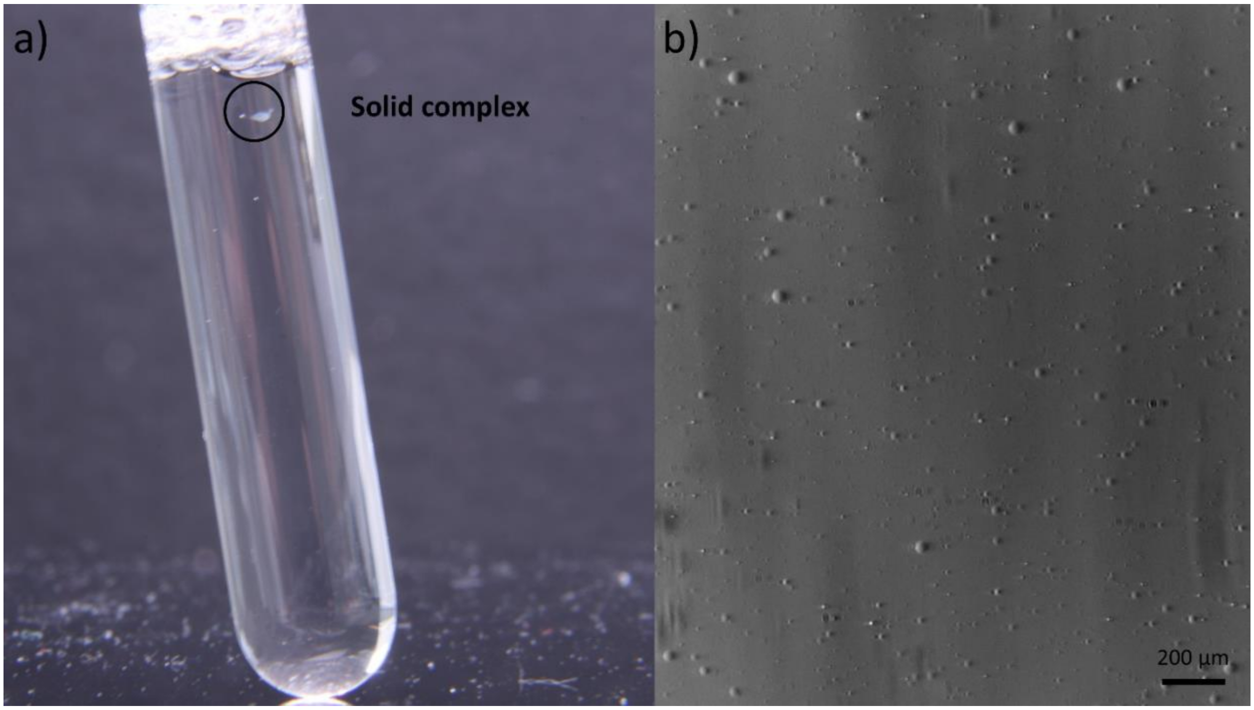

Figure S 59: a) Photograph of complex $59^{\oplus}$-$100^{\ominus}$ floating in a glass vial prepared with a KBr concentration of 125 mM. b) Microscope image of coacervate droplets of $87^{\oplus}$-$100^{\ominus}$ prepared with a KBr concentration of 1500 mM.

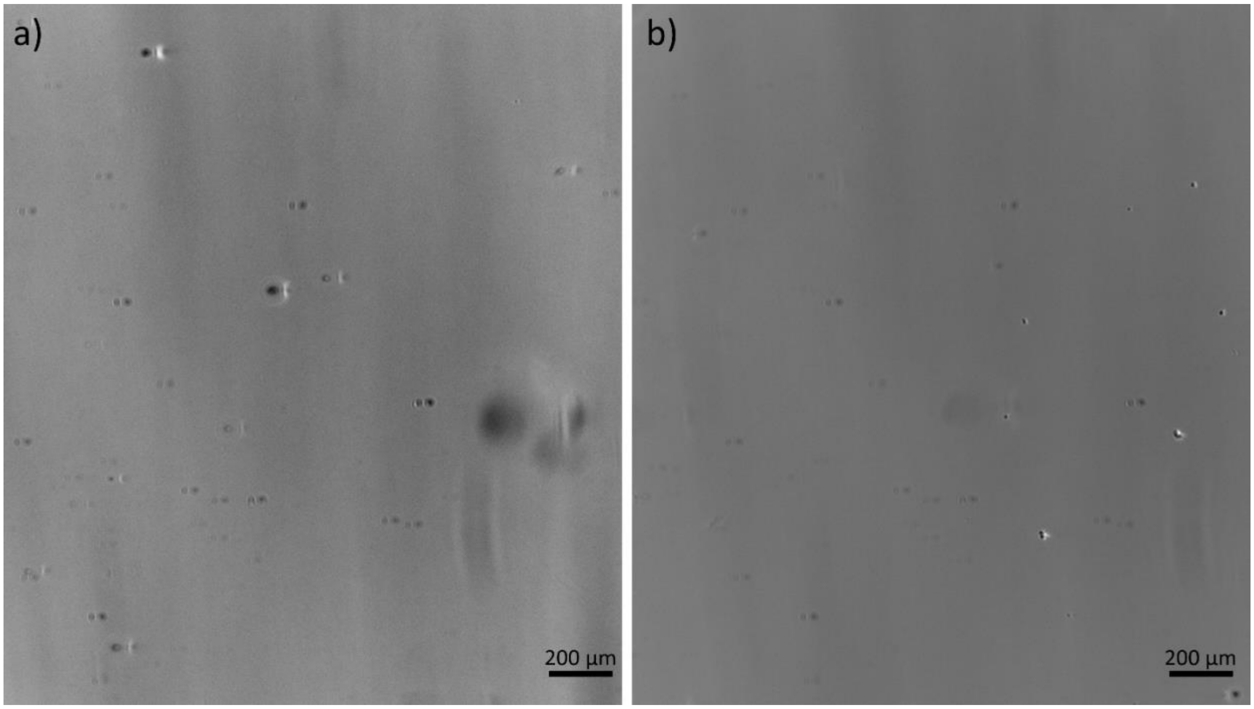

Figure S 60: Microscopic images of the complexes $87^{\oplus}$-$67^{\ominus}$ in a) and $87^{\oplus}$-$84^{\ominus}$ in b) close to the CSC at 1250 mM and 1500 mM KBr, respectively. No solid complex nor coacervate droplets could be detected at these specific salt concentrations.

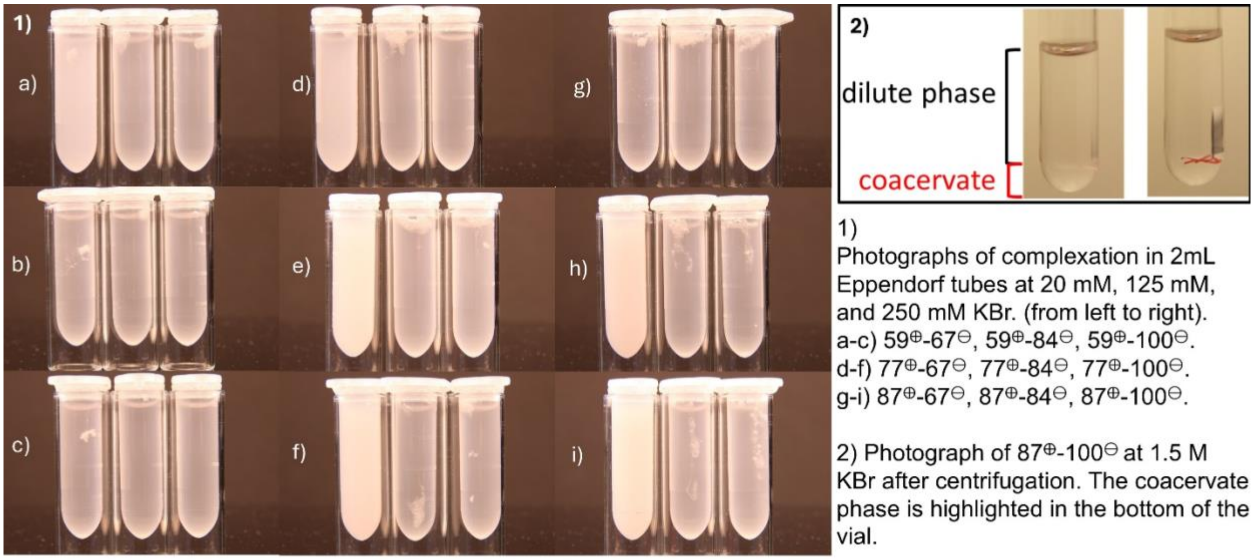

Figure S 61: Overview of (1) photographs of complexes at various salt concentrations and (2) photographs of a liquid-liquid phase separation of a PEC.

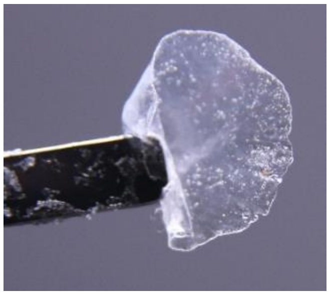

Figure S 62: Image of dried complex $59^{\oplus}$-$67^{\ominus}$ prepared with a KBr concentration of 125 mM.
